# Insights into Graphene Nanostructures, Fabrication Techniques, Mechanical, and Functional Behavior Characterization

**DOI:** 10.1002/smsc.202500272

**Published:** 2025-10-30

**Authors:** Ashfaqul Hoque Khadem, Camili Brignoni Diaz, Lihua Lou

**Affiliations:** ^1^ NanoBio Mechanics & Manufacturing Laboratory Department of Mechanical Engineering College of Engineering, Computing, and Applied Science Clemson University Clemson SC 29634 USA; ^2^ Department of Mechanical and Materials Engineering Florida International University Miami FL 33174 USA

**Keywords:** graphene, graphene composites, graphene nanostructures, mechanical characterization, nanomechanics

## Abstract

Graphene, a pioneering 2D carbon nanomaterial, has attracted significant attention owing to its exceptional structural, mechanical, thermal, and electrical performances. These intrinsic properties position it as a promising material platform for nanoelectromechanical systems, flexible electronics, and biomedical devices. Despite numerous existing reviews on graphene, a comprehensive assessment across graphene variants remains limited. Addressing this critical gap, this review provides an in‐depth overview of the structural configurations, physical properties, and application domains of key graphene forms—including monolayer, bilayer, few‐layer, and multilayer graphene, as well as functionalized derivatives. The review systematically discusses fabrication and synthesis strategies. Furthermore, it delves into state‐of‐the‐art methodologies for mechanical characterization, highlighting experimental and computational techniques, including in situ scanning electron microscopy and transmission electron microscopy, atomic force microscopy, nanoindentation, tensile testing, Raman spectroscopy, and multiscale simulations based on molecular dynamics, density functional theory, coarse‐grained modeling, and continuum mechanics. A comparative analysis of experimentally measured and computationally predicted mechanical properties is presented, elucidating existing discrepancies among methods. Collectively, this review aims to serve as a comprehensive reference for researchers at the intersection of nanomaterials, mechanics, and multifunctional material systems, offering a critical foundation for future research and the application of graphene nanostructures in next‐generation technologies.

## Graphical abstract

This review comprehensively explores the structural configurations, properties, and applications of graphene variants—including monolayer, multilayer, and functionalized forms (graphene oxide, rGO, composites). It discusses fabrication techniques (mechanical exfoliation, chemical vapor deposition, epitaxy) and advanced mechanical characterization methods, including experimental and computational (molecular dynamics, density functional theory) approaches, providing critical insights into discrepancies between predicted and measured mechanical properties.

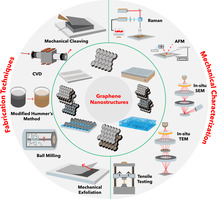

## 1 Introduction

Since the successful isolation of graphene in 2004, the field of 2D materials has experienced a transformative evolution in fundamental research and technological applications.^[^ ^1^ ^]^ Graphene's scientific appeal stems from its exceptional versatility and outstanding intrinsic properties. As a one‐atom‐thick planar crystal composed of sp^2^‐hybridized carbon atoms, graphene exhibits an ultra‐high thermal conductivity of ≈5000 W m^−1^ K^−1^,^[^ ^2^ ^]^ an electron mobility of up to 250 000 cm^2^ V^−1^ s^−1^ at room temperature,^[^ ^3^ ^]^ a theoretical surface area of 2630 m^2^ g^−1^,^[^ ^4^ ^]^ an in‐plane elastic modulus of ≈1 TPa,^[^ ^5^ ^]^ and electrical conductivity reaching 100 MS m^−1^.^[^ ^6^ ^]^ These exceptional physical, electrical, and mechanical properties have positioned graphene as a key candidate for a wide range of applications, including composite reinforcement,^[^ ^7^, ^8^ ^]^ field effect transistors (FETs),^[^ ^9^ ^]^ flexible electronics,^[^ ^10^ ^]^ supercapacitors,^[^ ^11^, ^12^ ^]^ and Joule heating devices.^[^ ^13^ ^]^

Graphene's 2D planar structure is characterized by a unique electronic configuration in which the conduction and valence bands converge at a single Dirac point, resulting in a zero bandgap semiconductor.^[^ ^14^ ^]^ While this contributes to its exceptional carrier mobility, it poses significant challenges for digital electronic applications, particularly where an energy bandgap is required to enable effective switching behavior. The absence of a bandgap leads to uncontrolled electron flow, elevated power dissipation, and limits the formation of efficient p–n junctions, which are essential for transistor operation.^[^ ^15^, ^16^ ^]^ To address these limitations, researchers have engineered a variety of graphene‐derived nanostructures that offer tunable electronic properties. These include graphene nanoribbons (GNRs),^[^ ^17^ ^]^ graphene drums,^[^ ^18^ ^]^ twisted bilayer graphene (TBLG),^[^ ^19^ ^]^ and multilayer graphene (MLG).^[^ ^20^ ^]^ Each of these architectures has demonstrated potential across diverse application domains, including FETs,^[^ ^9^ ^]^ semiconductor devices,^[^ ^21^ ^]^ and optoelectronics.^[^ ^22^ ^]^ Understanding and manipulating graphene nanostructures’ structure–property relationships is critical for optimizing their performance in advanced technologies. By precisely controlling parameters such as geometry, layer number, edge configuration, and defect concentration, researchers can tailor graphene's electrical, mechanical, and thermal properties for specific device requirements. This tunability enables the design of high‐performance systems in areas such as nanoelectronics, energy storage, sensing, and flexible electronics, where materials with finely tuned functionalities are essential for technological advancement.

Although numerous reviews have explored the mechanical properties of graphene nanostructures, the mechanical characterization techniques employed to assess these properties have not been systematically or thoroughly examined. For example, Sun et al.^[^ ^23^ ^]^ provided an overview of the mechanical behavior of graphene nanostructures but did not include key graphene derivatives such as graphene oxide (GO) or graphene‐based nanocomposites. Similarly, Papageorgiou et al.^[^ ^24^ ^]^ focused exclusively on graphene nanocomposites, without incorporating comparisons to other prominent graphene nanostructures or their corresponding computational modeling results. Akinwande et al.^[^ ^25^ ^]^ while discussing 2D materials, primarily addressed materials beyond graphene, and did not provide a detailed analysis specific to graphene nanostructures. Collectively, these studies do not comprehensively represent the current state‐of‐the‐art in experimental and computational mechanical characterization methods used across diverse graphene variants. This gap highlights the pressing need to organize and categorize existing literature in a more structured and comparative framework. Doing so would establish a clear roadmap for future research, enabling the systematic advancement of mechanical characterization techniques tailored to the wide variety of graphene‐based nanostructures.

Driven by the above‐listed gaps, this article aims to address existing gaps in literature by providing a comprehensive overview of graphene and its derivatives’ performances. It begins with a detailed review of various graphene variants, highlighting their structural configurations, key physical and mechanical properties, and application domains. The discussion then transitions to prominent synthesis techniques, such as mechanical exfoliation and chemical vapor deposition (CVD), commonly used in graphene preparation for mechanical evaluation. Another focus of the article is on mechanical characterization methods, with particular emphasis on Raman spectroscopy, atomic force microscopy (AFM), and related nanoscale measurement approaches. In addition, the review summarizes recent computational and simulation‐based studies, including molecular dynamics (MD), density functional theory (DFT), and finite element method (FEM), to provide complementary insights into the mechanical behavior of graphene nanostructures. The last section compares their mechanical property data obtained from experimental and computational techniques.

## 2 Overview of Graphene Nanostructures

**Figure 1:**
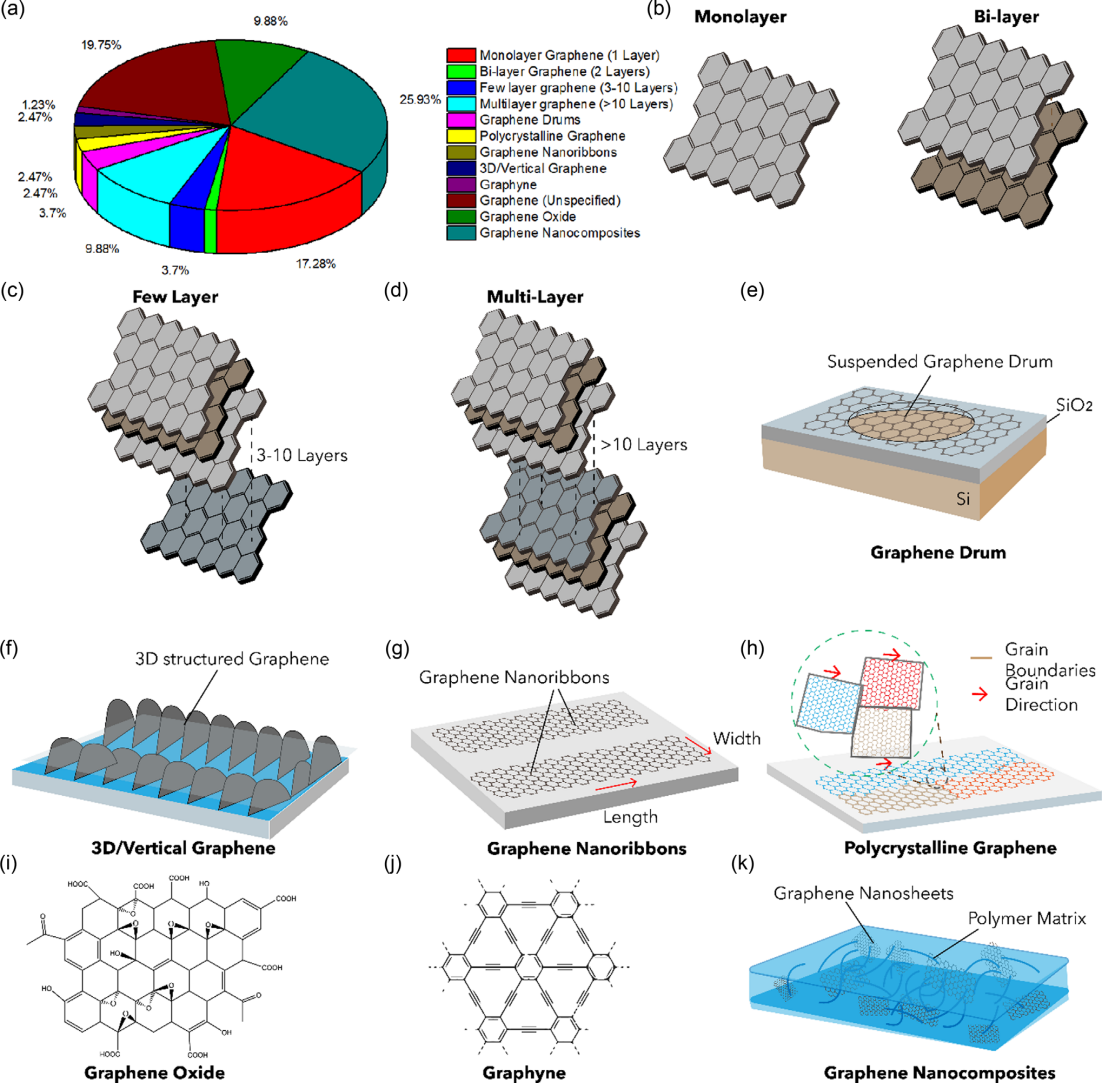
Schematic representation of various graphene nanostructures. a) Pie chart illustrating the distribution of graphene variants investigated in mechanical characterization studies. b) Schematic of MG and BLG, c) FLG, and d) MLG. e) Suspended graphene drums with membrane curvature. f) 3D or vertically oriented graphene architectures. g) GNRs with confined geometries. h) Polycrystalline graphene with GBs. i) GO containing oxygenated functional groups. j) γ*‐*Graphyne, a graphene allotrope with acetylenic linkages. k) Graphene nanocomposites.

Graphene exists in various nanostructured forms, differentiated by the number of layers, degree of functionalization, and intrinsic structural configurations. These characteristics can be precisely engineered to tailor graphene's performance for targeted applications by modifying it into distinct nanostructures such as graphene drums, GNRs, TBLG, and MLG. For example, graphene drums—suspended membranes with intrinsic curvature—exhibit tunable vibrational modes, making them highly attractive for nanoscale sensing applications.^[^ ^18^ ^]^ GNRs display quantum confinement effects, with their electronic properties strongly dependent on ribbon length and width.^[^ ^17^ ^]^ TBLG, when twisted at a specific “magic angle,” demonstrates unconventional superconductivity due to unique interlayer coupling mechanisms.^[^ ^19^ ^]^ Meanwhile, MLG exhibits enhanced mechanical robustness and long‐term durability, particularly when used as a reinforcement in composite matrices or as an active material in energy storage systems.^[^ ^20^ ^]^ Thus, the functional properties of graphene are closely governed by its layer‐dependent architecture and surface chemistry. **Figure** **1** illustrates representative graphene nanostructures reported in the literature. Figure 1a depicts pie chart distribution of graphene variants investigated in mechanical characterization studies. This section provides a comprehensive overview of these graphene variants, emphasizing their structural characteristics and electrical behavior.

### 2.1 Based on Number of Layers

#### 2.1.1 MG

**Figure 2:**
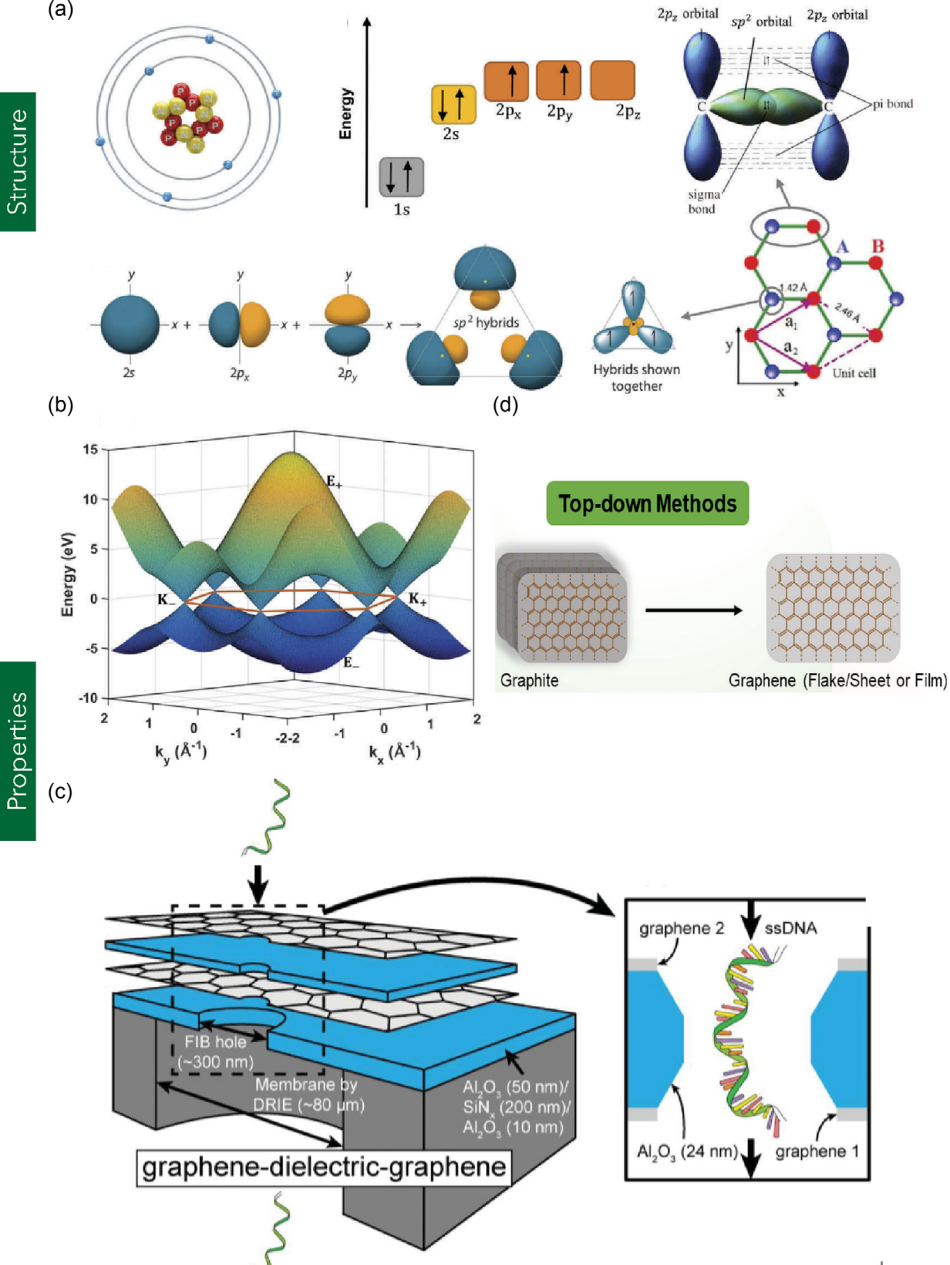
Fabrication routes and representative applications of MG. a) Schematic of carbon atomic structure, electronic energy levels, formation of sp^2^‐hybridized orbitals, and resulting graphene lattice. b) Electronic band structure of graphene calculated using the tight‐binding approximation. (a,b) are reproduced under the terms of CC‐BY license.^[^ ^26^ ^]^ Copyright 2018, The Authors, published by Taylor and Francis. c) Illustration of DNA translocation through an MG–Al_2_O_3_–MG nanodevice. Reproduced with permission.^[^ ^30^ ^]^ Copyright 2014, WILEY‐VCH Verlag GmbH. d) Top‐down exfoliation method for synthesizing graphene from bulk graphite. Reproduced with permission.^[^ ^389^ ^]^ Copyright 2021, Elsevier B.V.

Monolayer graphene (MG) refers to a single‐atom‐thick, 2D sheet of sp^2^‐hybridized carbon atoms arranged in a hexagonal crystal lattice composed of two interpenetrating triangular sublattices, with a carbon–carbon bond length of ≈0.141 nm (**Figure** **2**a,b).^[^ ^26^, ^27^ ^]^ Figure 1b shows the illustration of MG. Each carbon atom forms strong covalent bonds with three neighboring carbon atoms through the hybridized 2s, 2px, and 2py orbitals. The remaining unhybridized 2pz orbitals, oriented perpendicular to the graphene plane, overlap with adjacent 2pz orbitals to form delocalized π‐bonds across the lattice. This delocalized π‐electron network is responsible for graphene's exceptional electronic transport properties. At the Dirac point—where the conduction and valence bands intersect—the Fermi surface diminishes, enabling electron transitions with negligible energy input.^[^ ^28^ ^]^ As a result, charge carriers in graphene behave as massless Dirac fermions, giving rise to unique electronic characteristics that exhibit both metallic and semiconducting behavior, depending on the external conditions and perturbations applied.^[^ ^26^, ^29^ ^]^

The structural, electronic, and thermal properties of MG at the nanoscale (1–30 nm) are critical in determining the performance and functionality of graphene‐integrated nanodevices and nanosystems. For instance, a nanopore of ≈6 nm fabricated on MG within a graphene/Al_2_O_3_‐based nanodevice demonstrated the ability to detect both single‐stranded and double‐stranded DNA, with translocation speeds of 5.5 μs nt^−1^ and 0.4 μs bp^−1^, respectively (Figure 2c).^[^ ^30^ ^]^ The detection sensitivity in such platforms is governed by several factors, including the pore size and geometry (ranging from 3.5 to 525 nm), pore surface conditions, sheet thickness, and π–π interactions between MG and DNA nucleobases.^[^ ^31^ ^]^ These parameters not only regulate transport phenomena but are also intrinsically linked to the mechanical stability of suspended graphene sheets. This is because variations in pore size, sheet thickness, and interfacial interactions can influence the local stress distribution, fracture resistance, and overall structural robustness of the nanodevice. In terms of electronic behavior, Sordan et al.^[^ ^32^ ^]^ utilized the ambipolar conduction characteristics of MG, whereby the charge transport is mediated by either electrons (n‐type) or holes (p‐type), depending on the applied gate voltage. They engineered a single‐layer graphene field‐effect transistor exhibiting a charge neutrality point at 22.85 V. The Dirac point in the graphene electronic structure could be modulated within a ±5 V gate bias range, enabling switching between n‐type and p‐type behavior. Such gate‐induced electronic modulation has mechanical implications as well. Since electrostatic gating can introduce strain within the graphene lattice, altering its elastic response can potentially serve as a tool to engineer strain‐dependent mechanical properties in graphene devices. Furthermore, Evangeli et al.^[^ ^33^ ^]^ explored Joule heating and thermal dissipation in MG‐based nanodevices subjected to current densities as high as 1.25 × 10^8^ A cm^−2^. Due to MG's exceptional in‐plane thermal conductivity (≈5000 W m^−1^ K^−1^), rapid heat dissipation occurred across the MG–SiN interface, with the temperature rise limited to ≈11 K under high current flow. The interplay between thermal transport and mechanical integrity is crucial here. Since localized Joule heating can induce thermal stresses, modify defect dynamics, and accelerate mechanical degradation if not properly dissipated. Hence, thermal performance is directly associated with long‐term mechanical reliability.

MG serves as the fundamental structural unit of other carbon allotropes, including graphite, carbon nanotubes (CNTs), and fullerenes (e.g., C_60_). However, the bulk properties of these materials diverge significantly from those exhibited by MG at the nanoscale due to dimensionality and structural modifications. For example, graphite is formed by the vertical stacking of multiple MG layers into a 3D arrangement, where interlayer van der Waals interactions lead to a modified electronic band structure. Figure 2d depicts top–down fabrication of 2D MG from 3D structured graphite. In MG, charge carriers behave as massless Dirac fermions, exhibiting ultra‐high carrier mobility up to 200 000 cm^2^ V^−1^ s^−1^. In contrast, graphite exhibits significantly reduced mobility (≈3 000 cm^2^ V^−1^ s^−1^) due to interlayer coupling effects and altered electronic dispersion relations.^[^ ^34^, ^35^ ^]^ CNTs are synthesized by rolling a graphene sheet along a defined chiral vector, either through mechanical wrapping or molecular self‐assembly techniques. While pristine MG has a zero bandgap, CNTs exhibit finite bandgaps (ranging from 0.04 to 0.16 eV), arising from quantum confinement effects along their circumferential direction.^[^ ^36^, ^37^ ^]^ Additionally, 0D fullerenes (with diameters ≤ 1 nm) can be derived from MG via high‐energy beam‐induced atom removal processes.^[^ ^38^ ^]^ The resultant spherical carbon cages display tunable bandgaps (≈1.5–2 eV), primarily governed by geometric strain and distortion‐induced modulation of the π‐bonding network.^[^ ^38^, ^39^ ^]^ These examples collectively highlight those structural transformations of MG—through stacking, rolling, or irradiation—profoundly influence the resulting carbon nanostructures’ electronic and mechanical properties.

MG exhibits an exceptional theoretical specific surface area of ≈2630 m^2^ g^−1^^[^ ^40^ ^]^ and ultrahigh electron mobility reaching up to 200 000 cm^2^ V^−1^ s^−1^ at room temperature.^[^ ^3^ ^]^ Mechanically, MG demonstrates a remarkable Young's modulus of ≈1.1 TPa and an ultimate tensile strength of 130 GPa, indicating its outstanding stiffness and strength.^[^ ^41^ ^]^ Young's modulus, also known as the modulus of elasticity, is a fundamental mechanical property that measures a material's stiffness or its resistance to elastic deformation under tensile or compressive stress. Tensile strength, in contrast, often referred to as ultimate tensile strength (UTS), is the maximum amount of tensile stress a material can withstand before it breaks. In terms of transport properties, MG offers a high electrical conductivity of ≈6000 S cm^−1^^[^ ^42^ ^]^ and thermal conductivity up to 5000 W m^−1^ K^−1^, surpassing other carbon‐based nanomaterials such as single‐walled CNTs (SWCNTs).^[^ ^2^ ^]^ These exceptional properties make MG an attractive candidate for a broad spectrum of advanced applications, including FETs, nonvolatile memory devices, gas sensors, barrier coatings, and optoelectronic systems. For instance, the combination of near‐zero effective mass and high carrier mobility enables MG to function effectively in high‐frequency FETs. Both simulations and experimental studies have shown that MG‐based FETs exhibit symmetric ambipolar transport characteristics for hole and electron carriers, with a Dirac point positioned slightly above zero gate voltage.^[^ ^43^, ^44^ ^]^ In memory storage applications, MG‐integrated devices have demonstrated retention times of up to 24 h and stable performance over more than 3000 write–erase cycles without observable data degradation.^[^ ^45^ ^]^ Furthermore, MG's sensitivity to gas adsorption can induce a detectable Hall effect, enabling trace‐level detection of noble gases such as helium (He) and argon (Ar) with detection rates on the order of 10^3^  and 10^6^ atoms s^−1^, respectively.^[^ ^46^ ^]^ Additionally, MG's high optical transmittance (97.1% at 550 nm wavelength) and its ability to support surface plasmon‐polariton (SPP) interactions make it suitable for tunable photonic and optoelectronic devices. When integrated into dielectric stack configurations under a prism, the reflectivity of light can be modulated by inserting an MG layer and applying an external gate voltage (e.g., 3 V), thereby enabling dynamic control of optical intensity through electro‐optic modulation.^[^ ^47^, ^48^ ^]^

**Figure 3:**
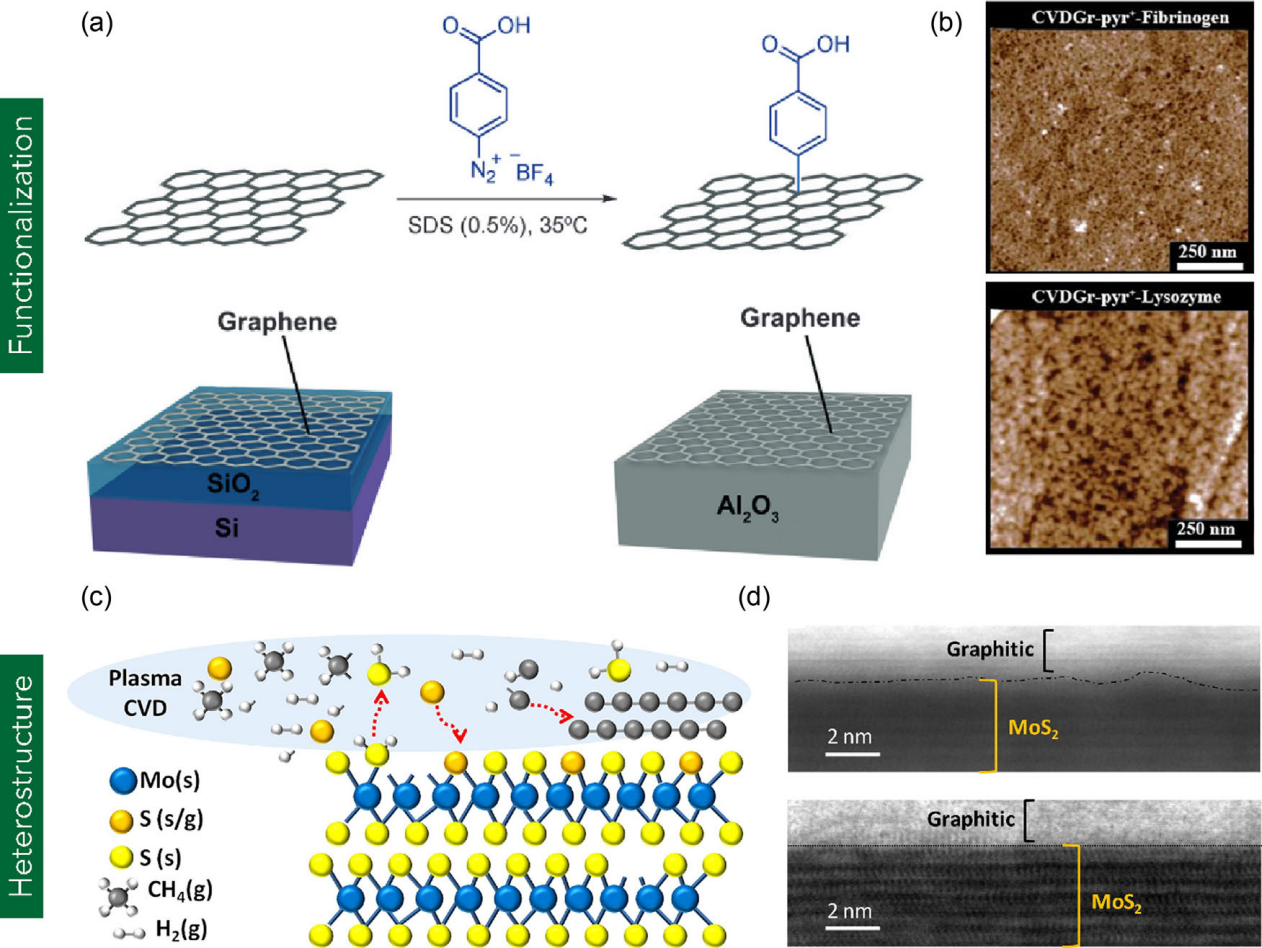
a) Covalent functionalization of MG with diazonium salts. Reproduced with permission.^[^ ^390^ ^]^ Copyright 2015, Wiley‐VCH Verlag GmbH. b) AFM topography of Pyr^+^‐functionalized MG after incubation with fibrinogen and lysozyme proteins. Reproduced with permission.^[^ ^55^ ^]^ Copyright 2016, Elsevier B.V. c) Schematic representation of atomic structure in a graphene–MoS_2_ van der Waals heterostructure. d) High‐resolution TEM cross‐sectional image of the graphene–MoS_2_ heterostructure. (c,d) are reproduced under terms of the CC‐BY license.^[^ ^67^ ^]^ Copyright 2021, The Authors, published by Elsevier B.V.

Due to the perpendicular orientation of delocalized π‐electrons in its electronic structure, MG exhibits exceptional electrical conductivity while remaining chemically inert to many gases and organic vapors.^[^ ^49^ ^]^ However, several intrinsic limitations—such as its insolubility in polar solvents, tendency to agglomerate via strong π–π interactions, and the absence of an intrinsic band gap—hinder its practical utility in solution‐based processing and semiconducting applications. To overcome these challenges, surface functionalization has emerged as a key strategy to enhance dispersibility, suppress aggregation, and introduce or tune the band gap of MG. For instance, Sun et al.^[^ ^50^ ^]^ achieved covalent functionalization of MG using 4‐bromophenyl groups, which resulted in a 70% increase in solubility in dimethylformamide compared with pristine MG. Similarly, Englert et al.^[^ ^51^ ^]^ functionalized MG with 4‐tert‐butylbenzene diazonium salts, effectively preventing agglomeration and yielding stable, aggregation‐free dispersions. In another study, Denis et al.^[^ ^52^ ^]^ demonstrated that functionalization with nitrene radicals could modulate the electronic structure of MG, inducing tunable band gaps of 0.67, 2.19, and 4.56 eV depending on the number of NH functional groups introduced. Surface functionalization of MG can be broadly classified into covalent and noncovalent approaches, involving interactions with foreign chemical species such as ions, molecules, or charge carriers. **Figure** **3**a illustrates the covalent functionalization of MG with diazonium salts. Covalent methods involve the formation of chemical bonds that typically rehybridize sp^2^ carbon atoms into sp^3^ configurations, which can compromise electrical conductivity. For example, Mishyn et al.^[^ ^53^ ^]^ employed 4‐[(triisopropylsilyl)ethynyl]benzene diazonium tetrafluoroborate (TIPS‐Eth‐ArN_2_^+^) to covalently functionalize MG, achieving an electron mobility of 1698 ± 536 cm^2^ V^−1^ s^−1^ and enabling selective detection of azidomethylferrocene for diagnostic applications. Although effective, such covalent functionalization partially disrupts the π‐conjugated network, thereby reducing the intrinsic conductivity of graphene.^[^ ^54^ ^]^ To preserve the native electronic properties while imparting additional functionalities, noncovalent functionalization is often preferred. For example, Navarro et al.^[^ ^55^ ^]^ modified CVD‐grown MG using two pyrene‐based derivatives—trimethyl‐(2‐oxo‐2‐pyren‐1‐yl‐ethyl)‐ammonium bromide and sodium (2‐oxo‐2‐pyren‐1‐yl‐ethyl)‐sulfonate. These derivatives interact with the graphene surface via π–π stacking, while their charged moieties (–NH_3_^+^ and –SO_3_^−^) introduce hydrophilicity without disrupting the sp^2^ carbon lattice. Figure 3b illustrates the AFM topography images of Pyr^+^‐functionalized MG. This non‐covalently functionalized MG exhibited selective protein adsorption, enabling electrostatic interaction‐based binding of fibrinogen and lysozyme, thereby demonstrating potential for biointerface and biosensing applications.

The properties of graphene can be significantly altered by ambient environmental factors. For example, humidity plays a pivotal role in modulating graphene's electronic properties. Water molecules adsorbed onto the graphene surface can act as electron acceptors, inducing p‐type doping and shifting the Fermi level toward positive gate voltages. This interaction is notably influenced by the underlying substrate, layer number, and exposure time. For instance, water adsorption can lead to a decrease in carrier mobility and an increase in surface roughness, impacting device performance.^[^ ^56^ ^]^ In addition, the presence of various gases in the atmosphere can significantly affect graphene's electronic characteristics. Oxygen and NO_2_ are known to absorb onto graphene, leading to hole doping and altering its conductivity. Conversely, ammonia can donate electrons, resulting in n‐type doping. These gas‐induced doping effects are crucial for applications such as gas sensors, but must be carefully controlled to maintain consistent device behavior.^[^ ^57^ ^]^ Temperature variations influence the carrier mobility in graphene. Elevated temperatures can enhance phonon scattering, leading to reduced mobility. Additionally, thermal expansion mismatches between graphene and its substrate can induce strain and wrinkles, further affecting electronic properties. Understanding and mitigating these temperature‐induced effects are essential for the reliable operation of graphene‐based devices.^[^ ^58^ ^]^

Recently, MG has been synthetically integrated with other atomically thin 2D materials to form layered assemblies known as van der Waals (vdW) heterostructures. These heterostructures leverage the absence of dangling bonds at the interfaces, enabling clean and tunable stacking of dissimilar 2D materials without lattice matching constraints. Commonly used materials for constructing MG‐based vdW heterostructures include hexagonal boron nitride (h‐BN),^[^ ^59^ ^]^ transition metal dichalcogenides (TMDs) (e.g., MoS_2_,^[^ ^60^ ^]^ WS_2_,^[^ ^61^ ^]^ MoSe_2_,^[^ ^62^ ^]^ WSe_2_,^[^ ^63^ ^]^ NbS_2_,^[^ ^59^ ^]^ and NbSe_2_^[^ ^59^ ^]^), and metal oxides (e.g., ZnO,^[^ ^59^ ^]^ WO_3_,^[^ ^64^ ^]^ and V_2_O_5_^[^ ^64^ ^]^). The fabrication of MG‐based vdW heterostructures can be achieved via several methods, including dry transfer (mechanical stacking),^[^ ^64^ ^]^ wet transfer, and direct CVD using a layer‐by‐layer (l‐b‐l) growth approach. In the dry transfer method, mechanically exfoliated or CVD‐grown layers are sequentially stacked using viscoelastic stamps. For example, Gupta et al.^[^ ^65^ ^]^ mechanically stacked h‐BN onto MG to fabricate a photodetector capable of harvesting light with an external quantum efficiency of ≈30%. The resulting heterostructure exhibited broadband photodetection capabilities, with sensitivity extending from 405 to 2000 nm.^[^ ^65^ ^]^ In the wet transfer technique, the underlying substrate of a CVD‐grown 2D layer is chemically etched, and the freestanding film is transferred onto another 2D material. Xu et al.^[^ ^66^ ^]^ employed this method to fabricate a MG/MoS_2_ heterostructure. They chemically etched the Cu foil supporting the graphene layer using FeCl_3_, and the resulting graphene film was transferred onto a CVD‐grown MoS_2_ substrate. The resulting MG/MoS_2_ heterostructure demonstrated ultrafast charge transfer dynamics (<100 fs) and a probe wavelength response at 660 nm, indicating strong potential for high‐speed photodetector applications. Alternatively, l‐b‐l direct growth techniques involve sequential CVD synthesis of 2D materials on one another. For example, Muñoz et al.^[^ ^67^ ^]^ directly grew MG onto a mechanically exfoliated MoS_2_ flake using plasma‐assisted CVD (Figure 3c,d). The resulting graphene–MoS_2_ heterostructure exhibited excellent electronic performance, including low contact resistance (<4 μΩ), high carrier mobility, on/off current ratios in the range of 10^6^–10^7^, and reduced threshold voltage, highlighting its suitability for high‐performance FET applications.^[^ ^67^ ^]^ In addition to the development of heterostructures and hybrid systems, future research on MG should emphasize its fundamental quantum and plasmonic behaviors. Key areas of interest include Dirac fermion transport, quantum Hall effects, and plasmon–exciton coupling, which are critical for advancing its applications in quantum electronic devices.

#### 2.1.2 BLG

**Figure 4:**
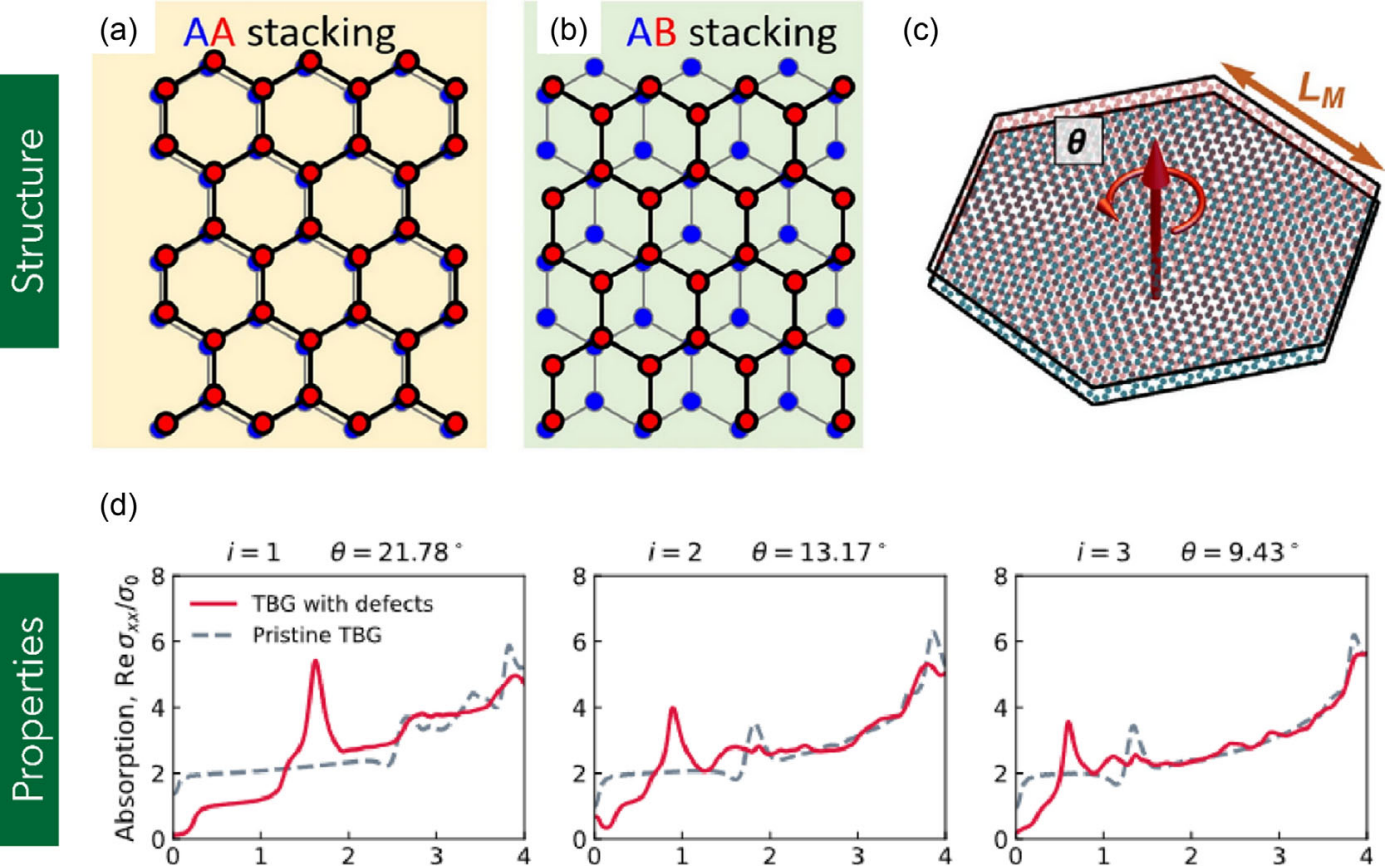
a,b) Atomic configurations of AA‐ and AB‐stacked BLG. Reproduced under terms of the CC‐BY license.^[^ ^391^ ^]^ Copyright 2016, The authors, published by Nature Portfolio. c) Schematic illustration of TBLG with a twist angle of *θ* = 3.9° and the corresponding moiré superlattice period *L*_M_. Reproduced under terms of the CC‐BY license.^[^ ^392^ ^]^ Copyright 2022, The authors, published by Nature Portfolio. d) Optical absorption spectra of TBLG at various twist angles (*i* = 1, 2, 3). Reproduced under terms of the CC‐BY license.^[^ ^80^ ^]^ Copyright 2023, The Authors, published by IOP Publishing.

Bi‐layer graphene (BLG), in contrast to MG, comprises two graphene sheets arranged in distinct stacking configurations, including AA, AB (Bernal), or twisted orientations characterized by a relative rotation angle (*θ* > 0) between the layers.^[^ ^68^ ^]^ In AA stacking, carbon atoms in both layers are aligned directly above one another, maintaining full atomic registry.^[^ ^69^ ^]^ In contrast, AB (Bernal) stacking involves a lateral shift of one layer such that half of the atoms in the top layer align with the centers of the hexagons in the bottom layer, resulting in a staggered configuration (**Figure** **4**a–c).^[^ ^69^ ^]^ The stacking arrangement and interlayer twist angle significantly influence the structural, electronic, and thermodynamic properties of BLG. Structurally, AA‐stacked BLG exhibits a mean interlayer spacing of ≈3.393 Å, whereas AB‐stacked BLG shows a slightly larger spacing of 3.405 Å.^[^ ^70^, ^71^ ^]^ The reduced spacing in AA‐stacking is attributed to its symmetric atomic alignment, which enhances interlayer van der Waals interactions. From an electronic perspective, DFT calculations indicate that AA‐stacked BLG possesses an intrinsic bandgap of 0.492 eV, which reduces significantly to 0.021 eV under an external electric field of 1 V nm^−1^.^[^ ^72^, ^73^ ^]^ Conversely, AB‐stacked BLG exhibits a tunable bandgap ranging from 0 to 0.27 eV under similar field conditions, reflecting its field‐dependent band structure modulation.^[^ ^72^ ^]^ Electrical conductivity is also affected by stacking type and interlayer spacing. At 300 K, AA‐stacked BLG demonstrates high electrical conductivity—23.6 × 10^4^ S m^−1^ at 3.55 Å spacing, which increases to 31.8 × 10^4^ S m^−1^ as the spacing expands to 4.00 Å.^[^ ^71^ ^]^ In contrast, AB‐stacked BLG displays a decreasing trend in conductivity with increasing interlayer distance: conductivity drops from 14.02 × 10^4^ S m^−1^ at 3.35 Å to 9.71 × 10^3^ S m^−1^ at 4.00 Å.^[^ ^71^ ^]^ Thermodynamically, AB‐stacked BLG is more stable than its AA counterpart, possessing a total energy that is 0.039 eV per atom lower, as confirmed by first‐principles calculations.^[^ ^73^, ^74^ ^]^ This enhanced stability, combined with its tunable bandgap and favorable electrical properties, makes AB‐stacked BLG a more suitable candidate for practical electronic and optoelectronic applications.

**Figure 5:**
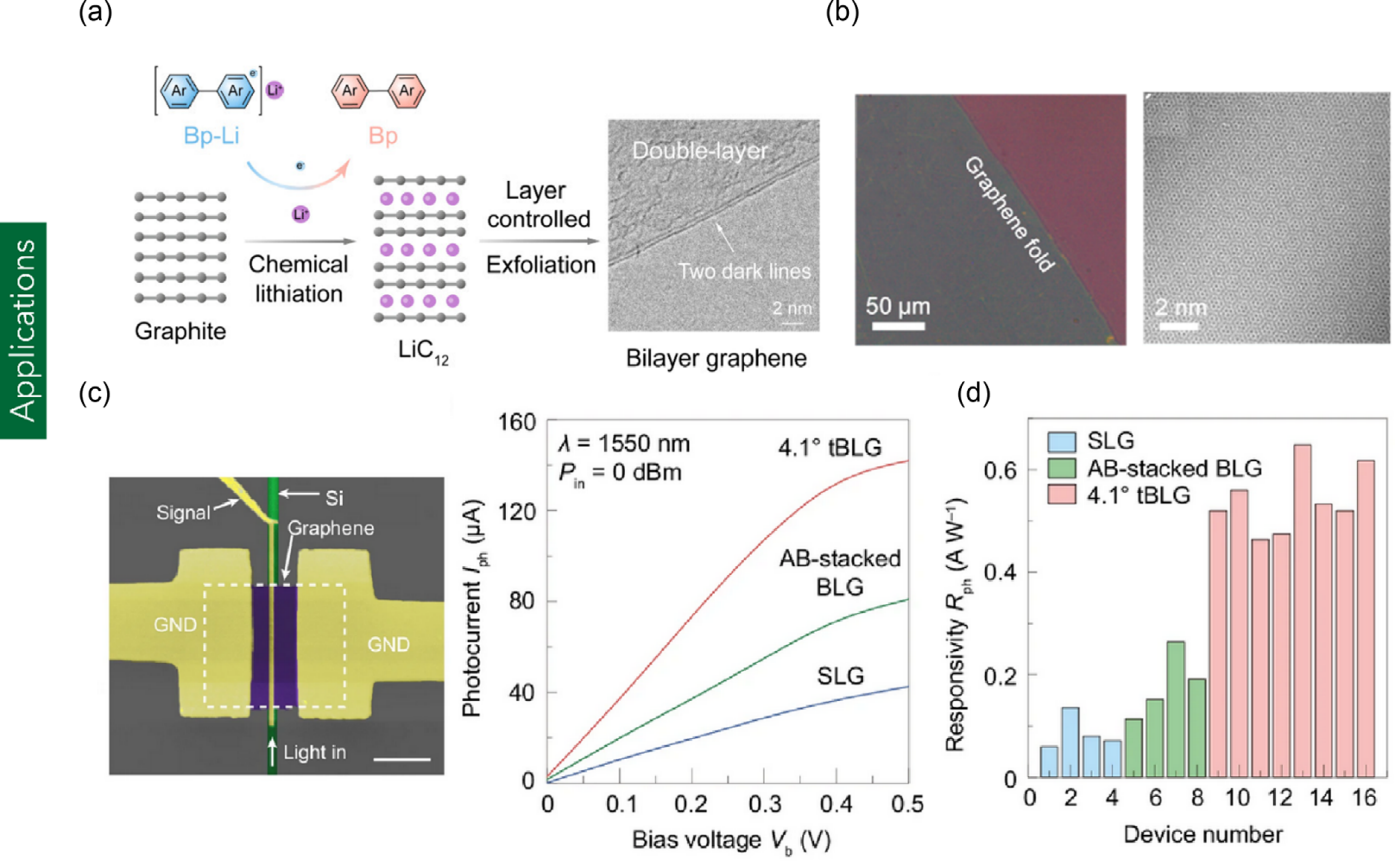
a) Schematic of the chemical lithiation‐assisted synthesis method for BLG. Reproduced with permission.^[^ ^237^ ^]^ Copyright 2024, American Chemical Society. b) Optical and TEM images of folded BLG illustrating structural continuity and interlayer stacking. Reproduced with permission.^[^ ^236^ ^]^ Copyright 2017, American Chemical Society. c) SEM image of a waveguide‐integrated TBLG photodetector (*θ* = 4.1°) and comparison of current–voltage (I–V) characteristics with AB‐stacked BLG and single‐layer graphene (SLG) photodetectors. d) Statistical performance comparison of TBLG (*θ* = 4.1°), AB‐stacked BLG, and SLG‐based photodetectors, including responsivity and bandwidth metrics. Reproduced under terms of the CC‐BY license.^[^ ^83^ ^]^ Copyright 2024, The authors, published by Nature Portfolio.

In addition to AA and AB stacked BLG, TBLG has garnered significant attention due to its unique electronic, optical, and superconducting properties, which are highly sensitive to the twist angle between the two graphene layers. TBLG refers to a bilayer configuration in which one graphene sheet is rotated relative to the other by a twist angle ranging from 0° to 30°, thereby breaking the symmetry found in conventional AB stacking and giving rise to moiré superlattices.^[^ ^75^ ^]^ The interlayer contact conductance of TBLG is strongly influenced by the twist angle. At 0° (perfect alignment), ICC is approximately four orders of magnitude higher than that observed at a 30° twist, owing to stronger interlayer electronic coupling and reduced decoupling effects between the misaligned layers (Figure 4d).^[^ ^76^, ^77^ ^]^ Additionally, the charge neutrality points voltage (*V*_cnp_), defined as the gate voltage at which no free charge carriers (electrons or holes) exist in the system, has also been shown to vary with twist angle. Kim et al.^[^ ^78^ ^]^ reported that *V*_cnp_ is ≈20.9 V for TBLG with twist angles between 0° and 9° and decreases to ≈16.9 V for twist angles between 9° and 30°, indicating a ≈20% reduction due to reduced carrier localization and band structure modifications at higher twist angles.^[^ ^79^ ^]^ Optical properties of TBLG also exhibit pronounced twist‐angle dependence. Natalin et al.^[^ ^80^ ^]^ investigated the optical absorption characteristics of pristine and defected TBLG systems at various twist angles. They observed that the absorption peak energies decreased systematically with twist angle: from 2.7 eV at 21.78°, to 1.8 eV at 13.17°, and to 1.3 eV at 9.43°. These changes are attributed to twist‐induced modulation of interlayer coupling and the emergence of moiré patterns, which significantly alter the joint density of states and transition probabilities (**Figure** **5**a,b). Remarkably, TBLG exhibits unconventional superconductivity near a so‐called “magic angle” of ≈1.1°, as first demonstrated by Cao et al. in 2018.^[^ ^81^ ^]^ At this angle, the electronic bands become flat, enhancing electron correlation effects that result in superconductivity with a critical temperature of 1.7 K. Subsequent studies by Yankowitz et al.^[^ ^82^ ^]^ further explored the tunability of superconducting behavior by modulating both twist angle and hydrostatic pressure. For instance, superconductivity was still observed at a slightly higher twist angle of 1.14°, although the critical temperature decreased to 0.4 K. Furthermore, applying a hydrostatic pressure of 2.21 GPa to TBLG with a twist angle of 1.27° enabled superconductivity at an elevated temperature of 3 K, demonstrating the interplay between geometric and external perturbations in controlling superconducting phases.

BLG has been extensively employed in the fabrication of advanced nanoelectronic and optoelectronic devices, including photodetectors and FETs, owing to its tunable band structure and superior carrier transport properties. For instance, Wu et al.^[^ ^83^ ^]^ developed a photodetector based on TBL with a controlled twist angle of 4.1°. At this angle, the formation of van Hove singularities in the electronic density of states significantly enhanced optical absorption. The resulting TBLG‐based photodetector exhibited a high responsivity of 0.65 A W^−1^ at telecom wavelengths (1550 nm), outperforming devices based on monolayer graphene and AB‐stacked BLG. Moreover, the device demonstrated a 3‐dB bandwidth exceeding 65 GHz and supported data transmission rates up to 50 Gbit s^−1^, highlighting its potential for high‐speed photonic and telecommunication applications (Figure 5c,d). In the realm of FETs, Icking et al.^[^ ^84^ ^]^ utilized an AB‐stacked BLG and h‐BN heterostructure to fabricate high‐performance graphene‐based transistors. These devices exhibited a field‐tunable bandgap and an inverse subthreshold slope as low as 250 μV dec^−1^ at cryogenic temperatures (0.1 K), approaching the Boltzmann thermionic limit of 20 μV dec^−1^. The transistors also demonstrated excellent switching performance, with on/off current ratios in the range of 10^4^ to 10^5^ and maintained a low drain current (≈10^−8^ A) at minimal source‐drain voltages, thereby confirming their suitability for low‐power, high‐sensitivity nanoelectronic applications.

The future research directions of BLG include in‐depth investigation of magic‐angle TBG to understand its emergent superconductivity and Mott insulating behavior, which hold significant potential for quantum computing and superconducting device applications. Additionally, further exploration of quantum transport phenomena in BLG under high magnetic fields—such as quantum Hall effects and broken symmetry states—will be critical for advancing its use in quantum electronic systems.

#### 2.1.3 FLG

Few‐layer graphene (FLG) refers to a stacked arrangement of graphene nanosheets comprising ≈3 to 10 atomic layers (Figure 1c), with total thicknesses ranging from less than 0.7 to ≈3.0 nm.^[^ ^85^, ^86^ ^]^ FLG preserves the characteristic hexagonal lattice of sp^2^‐hybridized carbon atoms found in monolayer graphene, with an interlayer spacing of ≈0.335 nm—comparable to that of bulk graphite.^[^ ^86^ ^]^ Common structural features in FLG include nanoscale wrinkles (often attributed to nanoparticle‐induced substrate roughness), spherical inclusions resulting from residual thermal stresses during rapid cooling, and edge reconstructions such as folding‐induced multilayer formation (e.g., four‐layer overlaps).^[^ ^87^ ^]^ Raman spectroscopic analysis of FLG typically reveals key vibrational modes: a G band near 1580 cm^−1^, indicative of in‐plane C–C bond stretching; a D band around 1352 cm^−1^, which reflects defect‐induced breathing modes; and a broadened 2D band at ≈2700 cm^−1^. The broadened and less symmetric 2D band confirms the presence of multiple graphene layers with weak interlayer electronic coupling.^[^ ^87^ ^]^ Despite the presence of structural defects and surface termination groups, FLG maintains high electrical conductivity (≈3.73 × 10^4^ S m^−1^), which exhibits notable dependence on temperature.^[^ ^88^ ^]^ Mechanically, FLG demonstrates robust performance comparable to monolayer graphene. Simulations have shown that the Young's modulus for one to five layers remains close to 1 TPa, consistent with that of MG.^[^ ^89^ ^]^ Additionally, the elastic modulus of FLG exhibits a near‐linear dependence on temperature in the range of 300–700 K, indicating thermal stability in mechanical response.^[^ ^89^ ^]^

**Figure 6:**
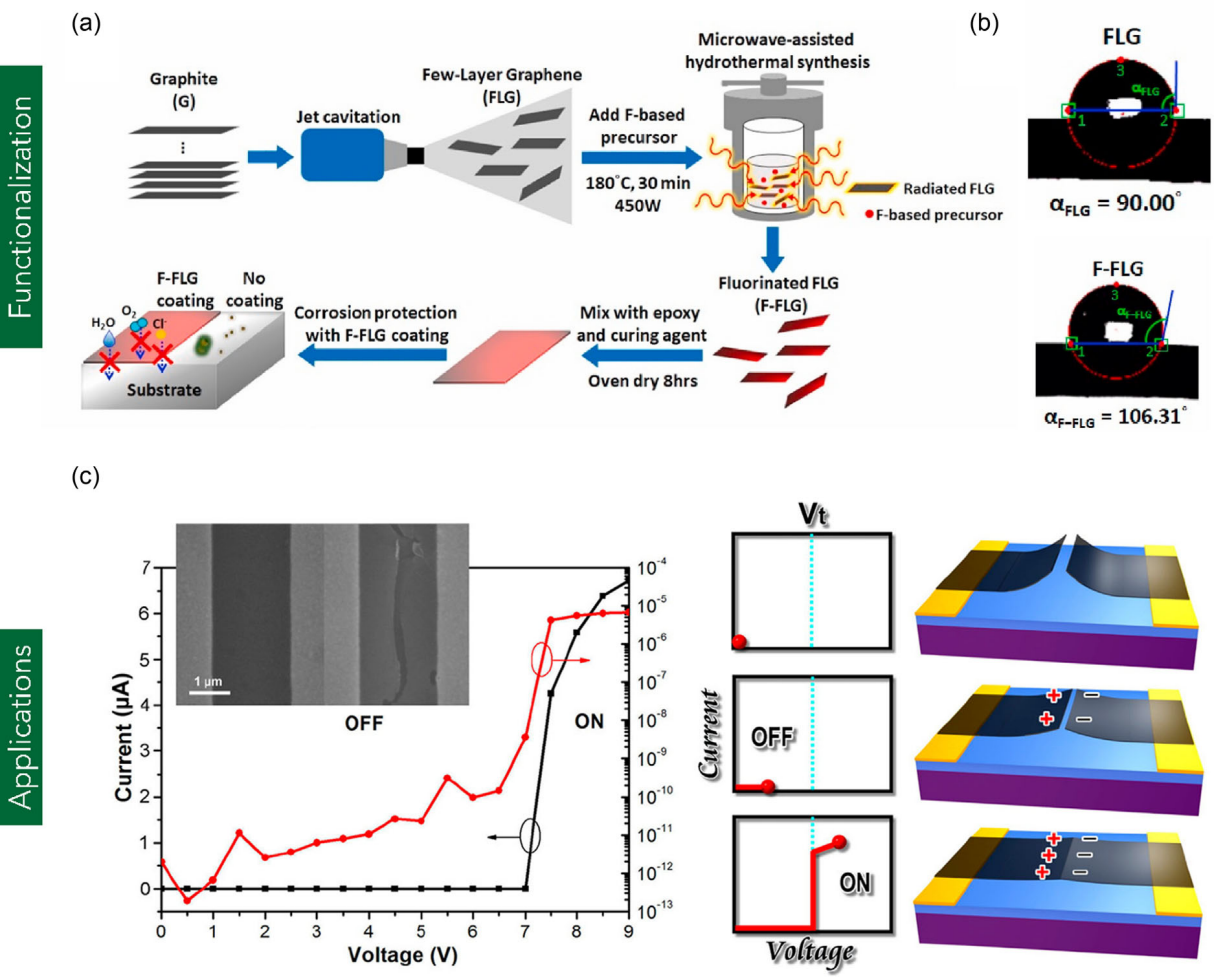
a,b) Schematic illustration of the fabrication process for functionalized FLG‐based composites and the corresponding water contact angle measurements, indicating surface wettability. (a,b) are reproduced with permission.^[^ ^92^ ^]^ Copyright 2023, Elsevier Ltd. c) I–V characteristics of FLG ribbon‐based NEMS devices. Reproduced with permission.^[^ ^93^ ^]^ Copyright 2013, American Chemical Society.

Surface functionalization is a widely adopted strategy for modifying the structural, electrical, and interfacial properties of FLG, enabling enhanced processability and functionality for targeted applications. For example, Nebogatikova et al.^[^ ^90^ ^]^ employed hydrofluoric acid treatment on FLG sheets with a thickness of ≈5 nm to tailor their surface morphology and electrical characteristics. Post‐treatment analysis revealed the formation of a corrugated, nanoshell‐like surface structure with a periodicity of 80–100 nm. However, this surface modification significantly disrupted the π‐electron delocalization network, resulting in a sharp increase in sheet resistivity to 10^11^ Ω sq^−1^, indicating a loss of electrical conductivity due to structural distortion. In contrast, Das et al.^[^ ^91^ ^]^ demonstrated a noncovalent functionalization approach using a triphenylene‐based aromatic stabilizer to improve the dispersion of FLG in aqueous media. This method facilitated the fabrication of uniform, conductive polyvinyl alcohol (PVA)/FLG nanocomposites at a low FLG content. Notably, the composite films achieved an electrical conductivity of 1.84 × 10^−4^ S m^−1^ at 3 vol% FLG—close to the percolation threshold of 0.26 vol%—highlighting the efficiency of noncovalent interactions in maintaining FLG's conductive network while enhancing compatibility with polymer matrices. Additionally, Hsia et al.^[^ ^92^ ^]^ utilized an environmentally friendly jet cavitation method to hydrothermally dope FLG with fluorine (F) atoms (**Figure** **6**a,b). When the F‐doped FLG was incorporated into an epoxy resin matrix and applied as a protective coating on cold‐rolled steel, a substantial increase in surface hydrophobicity was observed, with the water contact angle rising from 78.89° to 106.3°. The resulting coating also demonstrated enhanced anticorrosion performance, reducing the corrosion rate to 8.279 × 10^−3^ μm py^−1^—significantly lower than that of the uncoated steel substrate.

FLG has garnered significant attention for its versatility in various applications, including FETs, nanoelectromechanical systems (NEMS), photovoltaic anodes, and supercapacitor electrodes. For instance, Wei et al.^[^ ^93^ ^]^ employed CVD‐grown FLG to fabricate FETs exhibiting high carrier mobilities ranging from 300 to 1200 cm^2^ V^−1^ s^−1^ and an on/off current ratio of 10^3^ at a drain‐source voltage (*V*_ds_) of 0.5 V. Furthermore, the study demonstrated the integration of FLG into NEM switches, which achieved an exceptional on/off ratio of 10^9^, indicating superior switching performance (Figure 6c). In the context of photovoltaic applications, Wang et al.^[^ ^94^ ^]^ utilized FLG (6–10 layers) as an anode material in place of conventional indium tin oxide (ITO) in organic solar cells. The FLG‐based device exhibited a power conversion efficiency (PCE) of 1.71%, corresponding to ≈55.2% of the efficiency achieved by its indium tin oxide (ITO)‐based counterpart (PCE = 3.10%). This reduction in efficiency was primarily attributed to the relatively high contact resistance of FLG. In energy storage applications, Eskusson et al.^[^ ^95^ ^]^ synthesized Fe_3_O_4_–graphene nanocomposites using an ultrasonic irradiation method with FLG (two to five layers) as the conductive matrix. The resulting electrodes demonstrated a maximum energy density of 9.4 Wh kg^−1^ and a power density of 41.1 kW kg^−1^ when tested in Cs_2_SO_4_ and Rb_2_SO_4_ aqueous electrolytes, highlighting FLG's potential for high‐performance SC systems.

The future research directions of FLG include investigating layer‐dependent electronic structure and bandgap modulation, as well as elucidating the influence of stacking order on its electrical, magnetic, and optical properties.

#### 2.1.4 MLG

**Figure 7:**
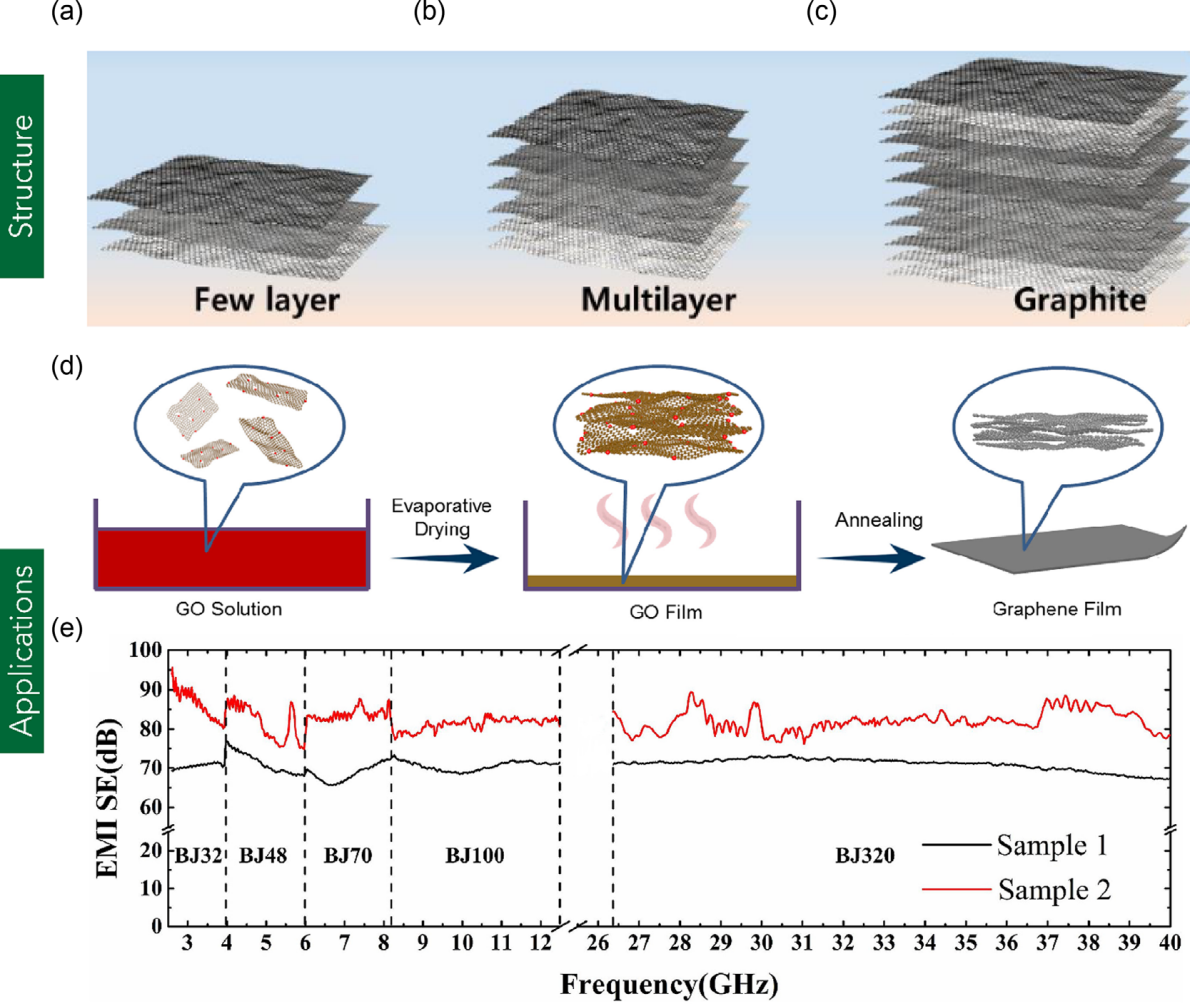
a–c) Structural comparison of FLG, MLG, and bulk graphite. Reproduced under terms of the CC‐BY license.^[^ ^393^ ^]^ Copyright 2022, The Authors, published by MDPI. d) Schematic of the fabrication process for MLG films involving high‐temperature thermal annealing and compression rolling. e) EMI shielding effectiveness of the MLG films over a frequency range of 2.6–40 GHz. (d–e) are reproduced with permission.^[^ ^105^ ^]^ Copyright 2019, Elsevier Ltd.

In contrast to FLG, MLG consists of platelet‐shaped graphene sheets comprising more than 10 atomic layers (Figure 1d).^[^ ^96^ ^]^ Due to its structural resemblance to ultrathin graphite, MLG is often referred to as graphene nanoplatelets (GNPs) or graphite nanoplatelets. Typically, MLG exhibits a surface area ranging from 50 to 750 m^2^ g^−1^ and a thickness spanning from 5 to 500 nm.^[^ ^97^, ^98^ ^]^ Owing to its relatively large lateral dimensions (often exceeding 100 nm), MLG possesses distinct optical and mechanical characteristics when compared to MG, as illustrated in **Figure** **7**a–c. Demetriou et al.^[^ ^99^ ^]^ reported that MLG exhibits nonlinear optical behaviors, including saturable absorption, characterized by an increase in transmittance at laser intensities around 4 MW cm^−2^, which is advantageous for applications in mode‐locking and Q‐switching laser systems. Furthermore, MLG also demonstrates two‐photon absorption, where transmittance decreases at higher intensities (above 5 MW cm^−2^), resulting in an optical limiting effect that is beneficial for the protection of sensitive optical sensors. From a mechanical standpoint, Gavallas et al.^[^ ^100^ ^]^ determined the in‐plane Young's modulus and planar shear stiffness of MLG to be 1080 GPa and 450 GPa, respectively. The high Young's modulus is attributed to the robust sp^2^‐hybridized carbon–carbon (C–C) covalent bonding within the hexagonal lattice structure. Conversely, the comparatively lower shear stiffness is primarily associated with the presence of double vacancy defects—regions where multiple carbon atoms are absent—leading to localized voids and a reduction in overall structural integrity.

MLG can be functionalized using various methods, including ozonation, solvothermal processing, irradiation techniques, and plasma treatment, with plasma‐based approaches being the most widely adopted due to their efficiency and solvent‐free nature. Among these, oxygen plasma treatment has emerged as a prominent technique for enhancing the surface reactivity of MLG. For instance, Keeley et al.^[^ ^101^ ^]^ applied oxygen plasma treatment to functionalize MLG (>10 layers) for electrochemical detection of biologically relevant molecules such as dopamine, nicotinamide adenine dinucleotide (NADH), ascorbic acid (C_6_H_8_O_6_), and uric acid. Post‐treatment, the MLG electrodes exhibited significantly enhanced peak currents for all analytes, demonstrating improved electrocatalytic activity and sensitivity. While oxygen plasma treatment offers a direct, solvent‐less route for functionalization, it can also be integrated with other exfoliation methods such as liquid phase exfoliation (LPE), electrochemical exfoliation, or thermal reduction to improve functional group incorporation and exfoliation efficiency. For example, Vega et al.^[^ ^102^ ^]^ employed a combined oxygen plasma–LPE strategy to functionalize graphite flakes and subsequently delaminate them into MLG sheets with fewer than 20 layers. This hybrid approach enhanced both the hydrophilicity and interlayer spacing of MLG, increasing from 0.345 to 0.347 nm, as a result of the incorporated oxygen functionalities.

MLG has been extensively utilized in diverse applications, including polymer composites, gas sensing, and electromagnetic interference (EMI) shielding. For example, Shen et al.^[^ ^103^ ^]^ developed MLG/epoxy composites via a solution mixing technique and evaluated their performance as thermal interface materials for electronic applications. The resulting composite was lightweight and exhibited a thermal conductivity of 1.5 W m^−1^ K^−1^ with only 2.8 vol% MLG loading, demonstrating its effectiveness in enhancing thermal transport. Lou et al.^[^ ^104^ ^]^ reinforced polyacrylonitrile/polyaniline (PAN/PANI) dual polymer system with MLG and iron oxide quantum dots (FeQDs) using a facile electrospinning process for EMI shielding applications. The ≈50 μm thick composite films achieved an electrical conductivity of 3 500 000 S m^−1^ and an EMI shielding effectiveness of 54 dB at low frequency and 170 dB in the X‐band (8–12 GHz), surpassing conventional metals. In another study, Fan et al.^[^ ^105^ ^]^ produced MLG films by thermally annealing GO at high temperatures (1300 and 2200 °C), followed by compression rolling. The resulting films exhibited high electrical conductivity and excellent EMI shielding effectiveness. Specifically, shielding values reached 70 dB for films with a thickness of 27 μm and up to 80 dB at 54 μm, over a broad frequency range of 2.6–40 GHz (Figure 7d–e), highlighting the potential of MLG films for advanced shielding applications. In the domain of gas sensing, Pena et al.^[^ ^106^ ^]^ fabricated MLG‐based sensors optimized for the detection of hazardous gases such as nitrogen dioxide (NO_2_), carbon monoxide (CO), and ammonia (NH_3_). These sensors employed ultraviolet (UV) photoactivation to enhance sensitivity and response kinetics. The devices demonstrated excellent performance, with a detection limit for NO_2_ as low as 30 parts per billion (ppb) and significantly improved responses to reducing gases like CO and NH_3_, making them suitable for real‐time environmental monitoring. Further research on MLG could focus on understanding interlayer coupling and anisotropic electronic transport to elucidate mechanisms of quantum tunneling and resonant charge transport across stacked layers.

### 2.2 Based on Patterned/Derived Structure

#### 2.2.1 Graphene Nanoribbons (GNRs)

**Figure 8:**
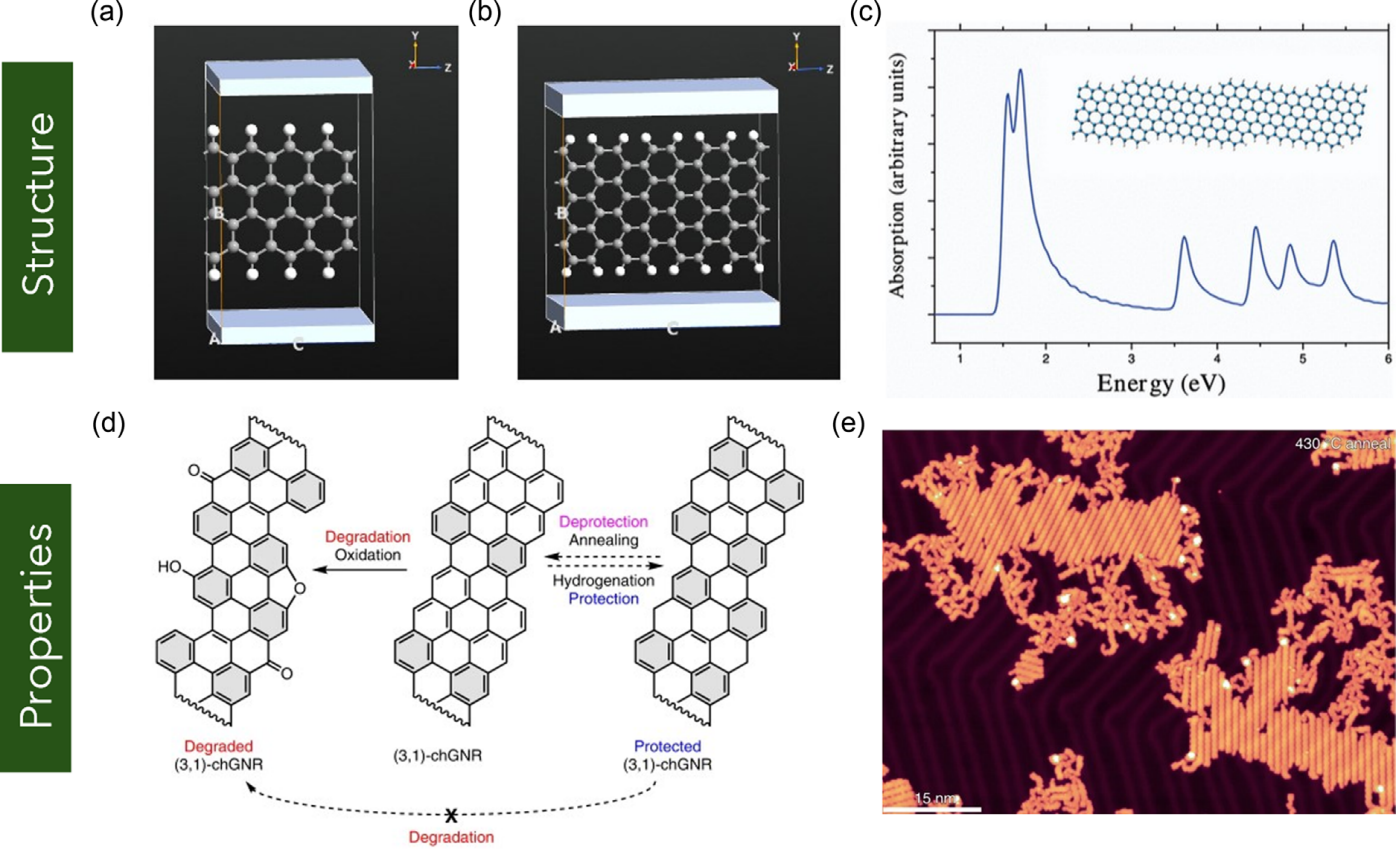
a,b) Atomic structures of GNRs with 8‐atom‐wide zigzag and armchair edge configurations, respectively. Reproduced under terms of the CC‐BY license.^[^ ^394^ ^]^ Copyright 2024, The Authors, published by MDPI. c) Normalized optical absorption spectra of GNRs calculated using the HSE/6‐31 G* hybrid functional level of theory. Reproduced with permission.^[^ ^109^ ^]^ Copyright 2006, American Chemical Society. d) Schematic representation of the edge hydrogenation mechanism for zigzag GNRs. e) STM images of GNRs post‐hydrogenation. (d,e) are reproduced under terms of the CC‐BY license.^[^ ^114^ ^]^ Copyright 2022, The Authors, published by Nature Portfolio.

GNRs are quasi‐1D nanostructures derived from narrow strips of MG, typically fabricated by unzipping or patterning graphene sheets (Figure 1g). Owing to their reduced dimensionality and high aspect ratio, GNRs exhibit unique electronic and magnetic properties influenced by their edge configuration and width. Two principal edge terminations—armchair and zigzag—govern the electronic behavior of GNRs.^[^ ^107^ ^]^ Armchair‐edged GNRs are predominantly semiconducting, with their bandgap exhibiting oscillatory behavior as a function of ribbon width, while zigzag‐edged GNRs tend to be metallic and may display spin‐polarized edge states, giving rise to edge‐localized magnetism (**Figure** **8**a,b).^[^ ^108^ ^]^ The electronic properties of GNRs, particularly bandgap, are strongly dependent on their width due to quantum confinement effects. Narrow GNRs with widths below 2 nm can exhibit bandgaps exceeding 1.0 eV, making them comparable to conventional semiconductors such as silicon and gallium arsenide (GaAs). For intermediate widths of 2–3 nm, the bandgap decreases to ≈0.67–0.70 eV, aligning with the bandgaps of materials like germanium (Ge) and indium nitride (InN) (Figure 8c). As the width increases further, GNRs (≈80 nm wide) display a significantly reduced bandgap (≈0.05 eV), approaching the semi‐metallic characteristics of pristine graphene.^[^ ^109^ ^]^ This width‐dependent bandgap modulation arises from quantum confinement, wherein narrower ribbons exhibit enhanced confinement, resulting in larger bandgaps and altered electronic structure.^[^ ^110^ ^]^ However, theoretical calculations provide a separate view. Owing to GNR's unique structure, the bandgap is influenced by geometry‐induced electron‐electron interactions. Yang et al.^[^ ^111^ ^]^ utilized the many‐body GW approximation for calculating the bandgap of GNRs and showed that the resulting values are larger than those predicted by previous DFT calculations. For arm‐chaired GNRs (AGNRs), a family‐dependent oscillatory trend was observed where bandgaps ranged from 0.5 to 3.0 eV for a width ranging from 0.4 to 2.4 nm. In addition, the bandgaps of the three families of AGNRs, classified by the number of carbon–carbon dimer lines (n = 3*p* *+* 1, 3*p*, or 3*p* *+ *2), maintain a qualitative hierarchy of *E*_g_^3*p*+1^ > *E*_g_^3*p*^ > E_g_^3*p*+2^. In contrast, the zigzag GNRs (ZGNRs) showed a GW correction of 0.8–1.5 eV, revealing that the bandgap at the zone boundary remained width‐insensitive due to the extreme localization of edge states. This high localization of edge states results in a larger self‐energy correction for these states. The effective modulus of GNR is highly dependent upon the ribbon width and edge atoms. For example, Lu et al.^[^ ^112^ ^]^ explained the edge‐effect‐induced stiffening or softening in GNRs using the following Equation (1).

(1) $$E_{\text{Effective}}=E_{\text{Bulk}}+\frac{2E_{\text{Edge}}}{W}$$

where $E_{\text{Effective}}$ is the width‐dependent Young's modulus of the GNR, $E_{\text{Bulk}}$ is the in‐plane modulus of defect‐free MG, *W* is the ribbon width, and $E_{\text{Edge}}$ is the edge modulus, indicating the elastic contribution from edge atoms. As the ribbon width (W) decreases, the edge‐to‐area ratio increases, making the second term ($\frac{2E_{\text{Edge}}}{W}$) significant. For wide ribbons (*W* > 10 nm), the edge contribution becomes negligible. However, in ultra‐narrow ribbons (<5 nm), edge reconstruction and passivation (e.g., hydrogen termination) can either increase or decrease the overall modulus, depending on the sign of $E_{\text{Edge}}$.

While MG exhibits relatively inert chemical behavior due to its delocalized π‐electron system and lack of reactive surface sites, GNRs are more chemically reactive and structurally unstable. However, the reactivity of GNRs is highly dependent on their edge structure and the surrounding chemical environment. In an oxygen‐rich atmosphere, the armchair edge configuration is the most thermodynamically stable, suggesting that GNRs will be less reactive and preferentially form armchair edges under these conditions. Conversely, in environments dominated by water or ammonia, the most stable configuration shifts to the zigzag edge (for water) or (for ammonia), indicating a higher propensity for ZGNRs to form and persist due to the effective passivation of their dangling bonds by OH/NH_2_ groups. Furthermore, nonaromatic edge configurations, which often exhibit metallic or magnetic properties due to partially occupied edge states, are generally less stable and more reactive.^[^ ^113^ ^]^ This instability arises primarily from the presence of unsaturated carbon atoms at the edges, which are significantly more reactive than the basal plane atoms of MG. Notably, zigzag‐edged GNRs are particularly susceptible to oxidative degradation and can begin to decompose even under low oxygen partial pressures (≈10^−5^ mbar).^[^ ^114^ ^]^ Although quantum confinement enables GNRs to display tunable electronic properties, it also enhances edge‐related effects, further diminishing their thermodynamic stability. To mitigate this issue, chemical modifications such as hydrogen passivation and ketone functionalization have been proposed. Studies have shown that hydrogen‐passivated GNRs with widths ranging from 5 to 80 nm can achieve cohesive energies up to 7.7 eV atom^−1^—comparable to that of pristine MG—and retain ≈90% structural integrity after 24 min of atmospheric exposure.^[^ ^109^ ^]^ In addition, Lawrence et al.^[^ ^114^ ^]^ demonstrated that edge functionalization with ketone groups using 2,2′‐dibromo‐10 H,10′H‐(9,9′‐bianthracenylidene)‐10,10′‐dione (k‐DBBA) significantly enhanced the oxidative stability of zigzag‐edged GNRs. This functionalization not only passivated the reactive edge sites but also increased the bandgap from 0.67 to 2.1 eV, thereby inhibiting oxidation and improving overall environmental stability (Figure 8d,e).

GNRs have been employed in various advanced device applications, including FETs, plasmonic gas sensors, and biosensing platforms, owing to their tunable electronic structure, high surface area, and edge‐state functionalities. For instance, Hashmi et al.^[^ ^115^ ^]^ utilized armchair‐edged GNRs to fabricate high‐performance FETs, specifically a GNRFET‐based triple‐cascade operational transconductance amplifier. This device demonstrated a substantial 33.88% increase in DC gain (29 dB), an 8.56‐fold improvement in gain bandwidth (215 MHz), and a 5.85‐fold enhancement in slew rate (29 939 V μs^−1^) compared with conventional complementary metal–oxide–semiconductor (CMOS)‐based designs. Furthermore, the GNRFET achieved a transconductance of 14.94 μS—over eight times that of standard CMOS—and reduced delay to 18.2 ps. Despite a slightly higher power consumption (3.86 μW), the energy‐delay product was significantly lower (1.278 × 10^−27^ W s^2^), underscoring the potential of GNRFETs for fast, low‐power electronics. Khaliji et al.^[^ ^116^ ^]^ explored the optoelectronic properties of GNRs, developing plasmonic gas sensors. The interaction of plasmonic fields with gas molecules resulted in detectable changes in optical extinction spectra, with extinction dips as small as 0.1% at low gas concentrations. Applying a 20 V bias across a 5 nm oxide layer amplified the local electrostatic field, enhancing sensor sensitivity and response time. In biosensing applications, Hasler et al.^[^ ^117^ ^]^ employed click‐chemistry‐based functionalization to integrate GNRs into biosensor platforms. GNRs provided a chemically stable and high‐surface‐area interface with enhanced probe density compared to conventional linker molecules. When incorporated into reduced graphene oxide (RGO)‐based FETs, the GNRs imparted dual‐mode sensing capabilities, enabling optical (via induced photoluminescence) and electrical detection, improving overall biosensor performance and versatility.

Future directions include developing novel on‐surface coupling reactions and improving CVD conditions to produce longer, high‐quality GNRs to expand the range of structures that can be synthesized without decomposition. Besides, future efforts should focus on improving the stability of sensitive GNRs through edge passivation (e.g., with ethylene or iodine) and heteroatom doping.^[^ ^118^, ^119^ ^]^ Additionally, exploring quantum transport phenomena and spin/valley physics in GNRs remains critical for advancing their applications in quantum and spintronic devices.

#### 2.2.2 Graphene Drums

**Figure 9:**
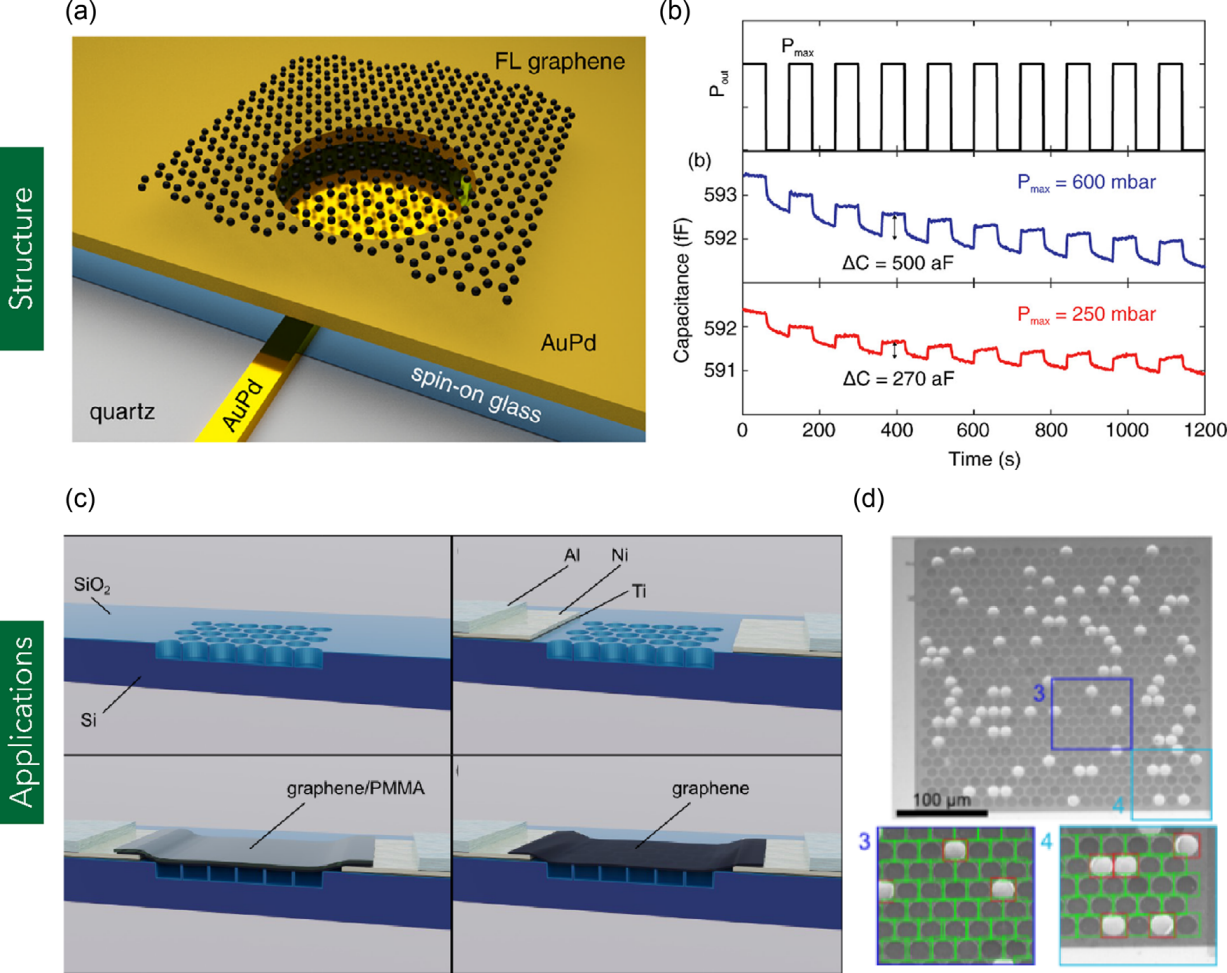
a) Schematic illustration of a graphene drum suspended over a metallic cavity, forming a nanocapacitor structure. b) Capacitance response of the graphene‐based nanodevice as a function of pressure variation from vacuum to a maximum of 600 mbar. (a,b) are reproduced with permission.^[^ ^395^ ^]^ Copyright 2017, American Chemical Society. c) Schematic depicting the integration of graphene drums into a gas sensor device. d) SEM images of an array of graphene drums, highlighting intact membranes (green) and damaged or partially ruptured membranes (red). (c,d) are reproduced under the terms of CC‐BY license.^[^ ^129^ ^]^ Copyright 2024, The Authors, published by American Chemical Society.

Graphene drums are nanomechanical resonators composed of graphene sheets suspended over circular or trench‐like cavities, analogous to a drumhead stretched over a frame (**Figure** **9**a,b). These structures are typically fabricated by transferring or exfoliating MG or MLG onto substrates pre‐patterned with etched circular cavities.^[^ ^120^ ^]^ Figure 1e illustrates the schematic of a graphene drum. Although MG is generally preferred due to its low mass and superior mechanical response, MLG has also been successfully employed for drum fabrication. During fabrication, gas molecules can become trapped between the graphene membrane and the underlying substrate. As these molecules diffuse into the cavities, they induce a pressure differential that causes the graphene sheet to bulge, forming the characteristic drum‐like geometry.^[^ ^121^ ^]^ The typical dimensions of these cavities range from 3 to 5 μm in diameter, with depths of ≈250 nm.^[^ ^122^ ^]^ Post‐processing treatments such as thermal annealing can modulate the suspended graphene membrane's out‐of‐plane deformation and in‐plane tensile strain. These treatments enable precise control of mechanical strain, reaching values up to 1.3%.^[^ ^123^ ^]^ Graphene drums exhibit exceptionally high resonance frequencies—often higher than 1 THz—making them ideal candidates for integration into NEMS. Their high sensitivity enables detection of minute mass changes with precision as fine as 354 nm Pa^−1^, facilitating applications in ultra‐sensitive mass and pressure sensing.^[^ ^124^ ^]^

When graphene is suspended over etched cavities or fabricated trenches, van der Waals forces between the graphene edges and the cavity walls can lead to partial adhesion, significantly influencing the effective boundary conditions. Instead of idealized clamped or simply supported edges assumed in mechanical models, real graphene membranes often experience sidewall sticking, which modifies stress distribution, resonance frequencies, and deflection profiles. Several studies have been conducted to investigate graphene drum adhesion to sidewall. For instance, Zong et al.^[^ ^125^ ^]^ reported that graphene membranes adhere to vertical SiO_2_ trench walls due to strong van der Waals interactions, with an adhesion energy of 151 ± 28 mJ m^−2^.^[^ ^125^ ^]^ In contrast, Koenig et al.^[^ ^56^ ^]^ found an adhesion energy of 0.45 ± 0.02 J m^−2^ for MG and 0.31 ± 0.03 J m^−2^ for FLG samples. Such a discrepancy arises from differences in experimental conditions, measurement techniques, and interfacial interactions. Zong et al.^[^ ^125^ ^]^ used dry nanoparticles as wedges in high vacuum, potentially underestimating adhesion due to trapped air and limited conformality, while Koenig et al.^[^ ^56^ ^]^ employed a pressurized gas environment that allowed graphene to conform intimately to the SiO_2_ substrate, enhancing van der Waals interactions. Additionally, the latter method accounts for full membrane elasticity and gas diffusion effects, resulting in higher, more realistic adhesion values closer to thermodynamic predictions.

Graphene drums, suspended over microcavities, exhibit ultrahigh resonance frequencies, reaching up to 1.176 THz in their pristine form.^[^ ^126^ ^]^ These resonance frequencies are highly sensitive to surface adsorption phenomena, whereby the attachment of foreign species—such as coronene, biphenyl, or fullerene molecules—induces measurable frequency shifts. This drift in resonance is directly correlated with the mass, geometry, and interaction strength of the adsorbed species, allowing graphene drums to function as highly precise nanomechanical mass sensors capable of detecting minute mass variations with exceptional sensitivity.^[^ ^127^ ^]^ The vibrational modes of the ultrathin graphene membrane are significantly influenced by the physical characteristics of the adsorbates, including molecular diameter, surface functionalization, and binding affinity. In addition to their resonance‐based sensing capabilities, graphene drums also demonstrate remarkable mechanical robustness under compressive loading. For example, Zhao et al.^[^ ^122^ ^]^ integrated graphene drums with carbon aerogels to fabricate a broadband dual‐wave attenuation system. These composite structures exhibited superior compressive strength, sustaining pressures up to 150 kPa at 80% compressive strain, while maintaining structural integrity and fatigue resistance over 1000 loading cycles.

Graphene drums have been employed in various micro‐ and nanoscale device applications, including acoustic absorption, pressure sensing, and microelectromechanical systems (MEMS)‐based microphone technologies, owing to their ultrathin structure, high mechanical sensitivity, and excellent resonance characteristics. For example, Pang et al.^[^ ^128^ ^]^ utilized graphene drums for cellular acoustic absorption applications. The ultrathin suspended membranes demonstrated a high sound absorption coefficient ranging from 0.8 to 0.9 across a broad frequency range of 200–6000 Hz. A 30 mm‐thick absorber configuration achieved a noise reduction coefficient of 0.58. Additionally, 20 nm‐thick graphene drums exhibited an out‐of‐plane resonance amplitude of 14.59 μm at 200 Hz, confirming their suitability for broadband acoustic absorption. Lkuas et al.^[^ ^129^ ^]^ developed piezoresistive pressure sensors based on graphene drums for NEMS applications. The fabricated sensors, with membrane diameters ranging from 3.4 to 12 μm, displayed pressure sensitivities between 0.5 and 3.0 × 10^−6^ mbar^−1^. The devices demonstrated maximum sensitivity in the 850–950 mbar range, with gauge factors of –0.46 and –0.61 under tensile and compressive strain, respectively, reflecting their responsiveness to external pressure variations. In microphone applications, Baglioni et al.^[^ ^130^ ^]^ employed graphene drums as highly sensitive mechanical transducers. The resulting devices achieved a mechanical sensitivity of 2000 nm Pa^−1^—two orders of magnitude greater than conventional MEMS microphones (≈1.3 nm Pa^−1^). Moreover, the graphene drum‐based microphone exhibited a limit of detection (LOD) of 15 dB sound pressure level (SPL) and a signal‐to‐noise ratio (SNR) in the range of 80–95 dB, comparable to commercial MEMS microphone systems (Figure 9c,d).^[^ ^130^ ^]^

The future research directions for graphene drums include investigating ultrahigh‐frequency mechanical resonances (in the GHz to THz range) and exploring their behavior near the quantum limit, with potential applications in quantum sensing, signal transduction, and fundamental studies of nanomechanical systems.

### 2.3 Based on Structure and Functionalization

#### 2.3.1 PG

**Figure 10:**
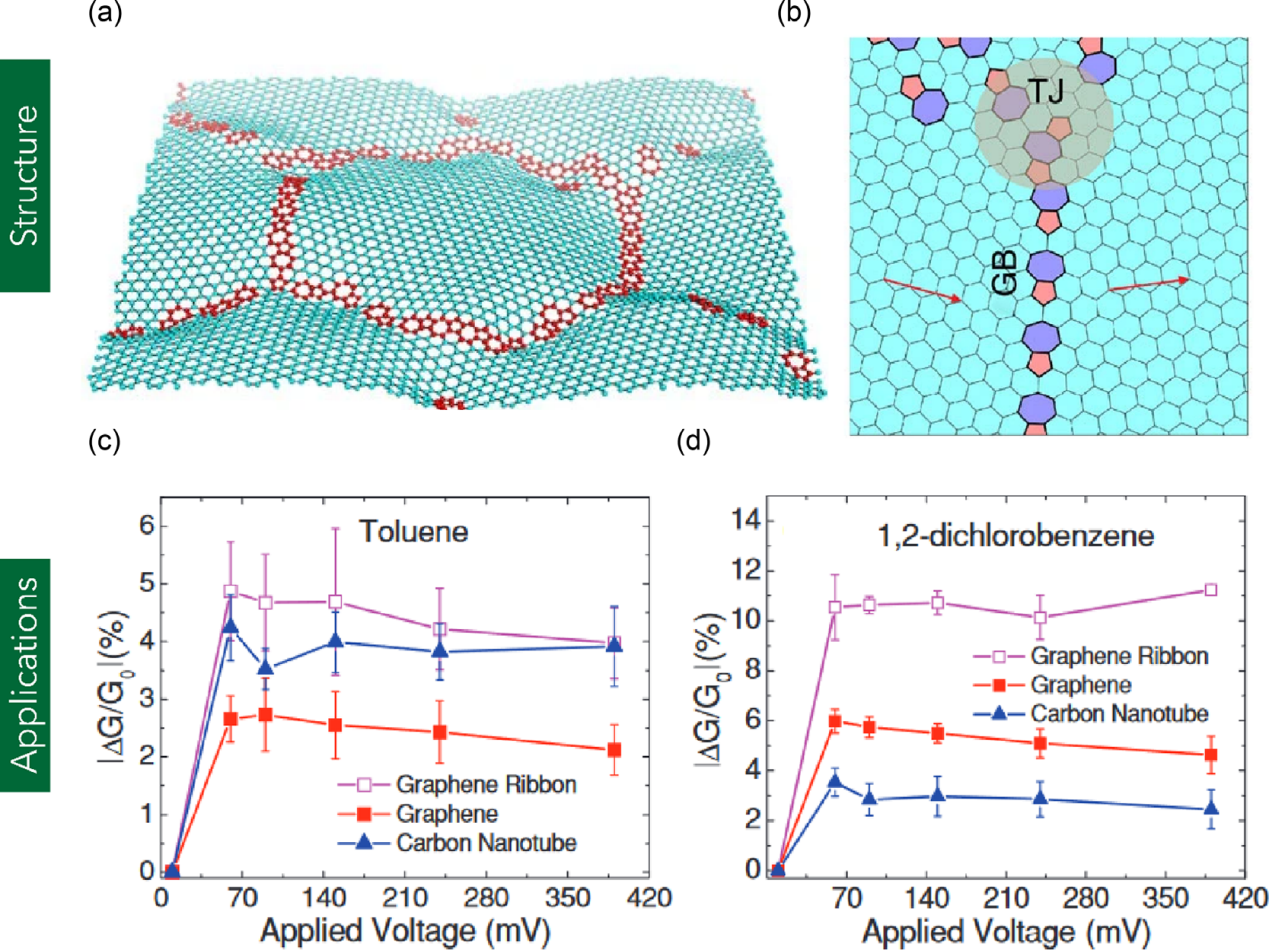
a) Schematic representation of PG. b) Enlarged schematic view of the grain boundary region. (a,b) are reproduced under the terms of the CC‐BY license.^[^ ^305^ ^]^ Copyright 2016, The Authors, published by Nature Portfolio. c,d) Sensitivity response (ΔG/G_0_) of PG nanoribbons, pristine graphene, and CNTs toward toluene and 1,2‐dichlorobenzene as a function of applied voltage. (c,d) are reproduced with permission.^[^ ^140^ ^]^ Copyright 2012, WILEY‐VCH Verlag GmbH.

Polycrystalline graphene (PG) consists of multiple single‐crystalline graphene domains (grains) interconnected by grain boundaries (GBs), which serve as topological defects—most commonly composed of pentagon‐heptagon (5–7) ring pairs (**Figure** **10**a,b).^[^ ^131^ ^]^ Figure 1h illustrates the schematic of PG. Due to the structural discontinuities and defect states at GBs, PG exhibits markedly different properties compared to MG. Electrically, PG demonstrates significantly lower charge carrier mobility relative to pristine MG. While MG can achieve carrier mobilities exceeding 10 000 cm^2^ V^−1^ s^−1^, the mobility in PG is often reduced to a range of 1 000–5 000 cm^2^ V^−1^ s^−1^.^[^ ^132^ ^]^ This reduction is primarily attributed to charge scattering and localization effects at the GBs, where the electronic states form p–n junction‐like barriers that impede carrier transport and reduce the effective carrier density.^[^ ^133^ ^]^ Mechanically, PG retains a high in‐plane elastic modulus of ≈1 TPa, comparable to that of MG. However, its fracture strength, which is the stress at which a material fails or breaks into two or more pieces, is typically reduced by up to 40%, primarily due to stress concentration and mechanical mismatch across the GBs.^[^ ^134^ ^]^ Thermally, PG also exhibits diminished performance compared with MG. While MG can reach thermal conductivities as high as 5000 W m^−1^ K^−1^, PG shows lower values in the range of 1000–2500 W m^−1^ K^−1^, with thermal transport strongly dependent on grain size and boundary density.^[^ ^135^ ^]^ The reduction is predominantly caused by enhanced phonon scattering at grain boundaries, which disrupts the propagation of heat‐carrying phonons and limits effective thermal conduction. PG demonstrates GB‐dependent mechanical properties. According to the Hall–Petch relation (Equation 2),^[^ ^136^ ^]^ the fracture strength of PG should reduce with an increase in GB size.

(2) $$\sigma_{f}=\sigma_{o}+\frac{k}{\sqrt{\rho_{\text{GB}}}}$$

where $\sigma_{f}$ is the fracture strength, $\sigma_{o}$ represents the lattice friction stress, *k* is the material‐dependent strengthening constant, and $\rho_{\text{GB}}$ is the average grain size of PG. However, an opposite trend was observed for PG as reported by Sha et al. (Equation 3).^[^ ^137^ ^]^

(3)

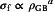

where *α* is the scaling exponent obtained from MD simulations. This power‐law indicates that as grain size decreases, the breaking strength also decreases, opposite to the traditional Hall–Petch effect.

PG has been extensively explored for use in optoelectronic and sensing applications, including photodetectors, FETs, and chemiresistive sensors, owing to its tunable electronic properties and large‐area processability. For instance, Li et al.^[^ ^138^ ^]^ employed a plasma‐assisted CVD technique to synthesize PG directly on Si substrates at a relatively low temperature of 700 °C, eliminating the need for metal catalysts and subsequent transfer steps. The resulting graphene/Si Schottky junction was utilized as a photodetector, demonstrating a responsivity of 52 mA W^−1^ and a detection limit of ≈1.4 × 10^10^ cm Hz^1/2^ W^−1^ under 1550 nm illumination. The direct growth approach enabled efficient device integration without additional post‐processing. In the context of FET applications, Jiménez et al.^[^ ^139^ ^]^ investigated the influence of grain boundaries in PG using multi‐scale computational modeling. Their study revealed that grain boundaries introduced electronic disorder, resulting in a degradation of both maximum and cutoff frequencies, thereby adversely affecting transconductance. However, the simulations also indicated improved current saturation behavior in the presence of grain boundaries, albeit with minimal intrinsic gain, highlighting the complex trade‐offs between structural disorder and device performance. PG has also shown promise in chemiresistive sensing platforms. Salehi–Khojin et al.^[^ ^140^ ^]^ utilized CVD‐grown PG on Si/SiO_2_ substrates to fabricate chemiresistive sensors for detecting volatile organic compounds (VOCs) such as toluene and 1,2‐dichlorobenzene. The PG sensors, integrated with metal electrodes, exhibited significant conductance modulation (ΔG/G_0_), with responses of ≈50% for 1,2‐dichlorobenzene and 3%–4% for toluene. Furthermore, the voltage threshold for detection exceeded 50 mV, demonstrating superior sensitivity and selectivity compared with monolayer graphene and CNT‐based sensors (Figure 10c,d).

PG can be functionalized through various methods, including ozone treatment, plasma‐assisted hydrogenation, and wet chemical modification, enabling tunability of its electronic and mechanical properties for device‐specific applications. For example, Seifert et al.^[^ ^141^ ^]^ performed dual‐step functionalization of CVD‐grown PG using ozone exposure followed by plasma‐based hydrogenation. Initially, PG samples were exposed to ozone at a flow rate of 280 sccm and a temperature of 70 °C, followed by hydrogen plasma treatment conducted at 0.9 bar with an acceleration voltage of 275 V. This approach introduced a controlled defect density (10^−4^ to 10^−3^), which led to a reduction in field‐effect mobility and increased electrical breakdown susceptibility, demonstrating the influence of defect engineering on transport properties. Bissert et al.^[^ ^142^ ^]^ investigated strain modulation in PG subjected to wet chemical functionalization. CVD‐grown PG films were immersed in nitrobenzene diazonium tetrafluoroborate (NBD) solutions with concentrations ranging from 2 to 40 nM. The surface functionalization introduced p‐type doping characteristics, which led to a decrease in the Raman ID/IG ratio and a G‐band shift of 10.9 cm^−1^ after 5 min of exposure, indicating successful surface modification and strain enhancement. The ID/IG ratio typically indicates the intensity ratio of the D band to the G band in the Raman spectrum of graphene nanostructures. It is used to quantify the number of defects or disorder relative to the graphitic order. While a low ID/IG ratio indicates high crystallinity and good‐quality graphene, a high ratio means more defects or disorder in the nanostructure.^[^ ^143^, ^144^ ^]^ In addition to experimental studies, computational investigations have provided insights into the mechanical implications of PG functionalization. Elapolu et al.^[^ ^145^ ^]^ employed MD simulations to assess the mechanical and fracture behavior of hydrogen‐functionalized PG. Their results revealed that pristine PG exhibited the highest elastic modulus (853.3 ± 0.9 GPa), while hydrogenation led to a notable reduction in stiffness due to structural mismatch and stress concentration at the grain boundaries.

#### 2.3.2 Graphyne

**Figure 11:**
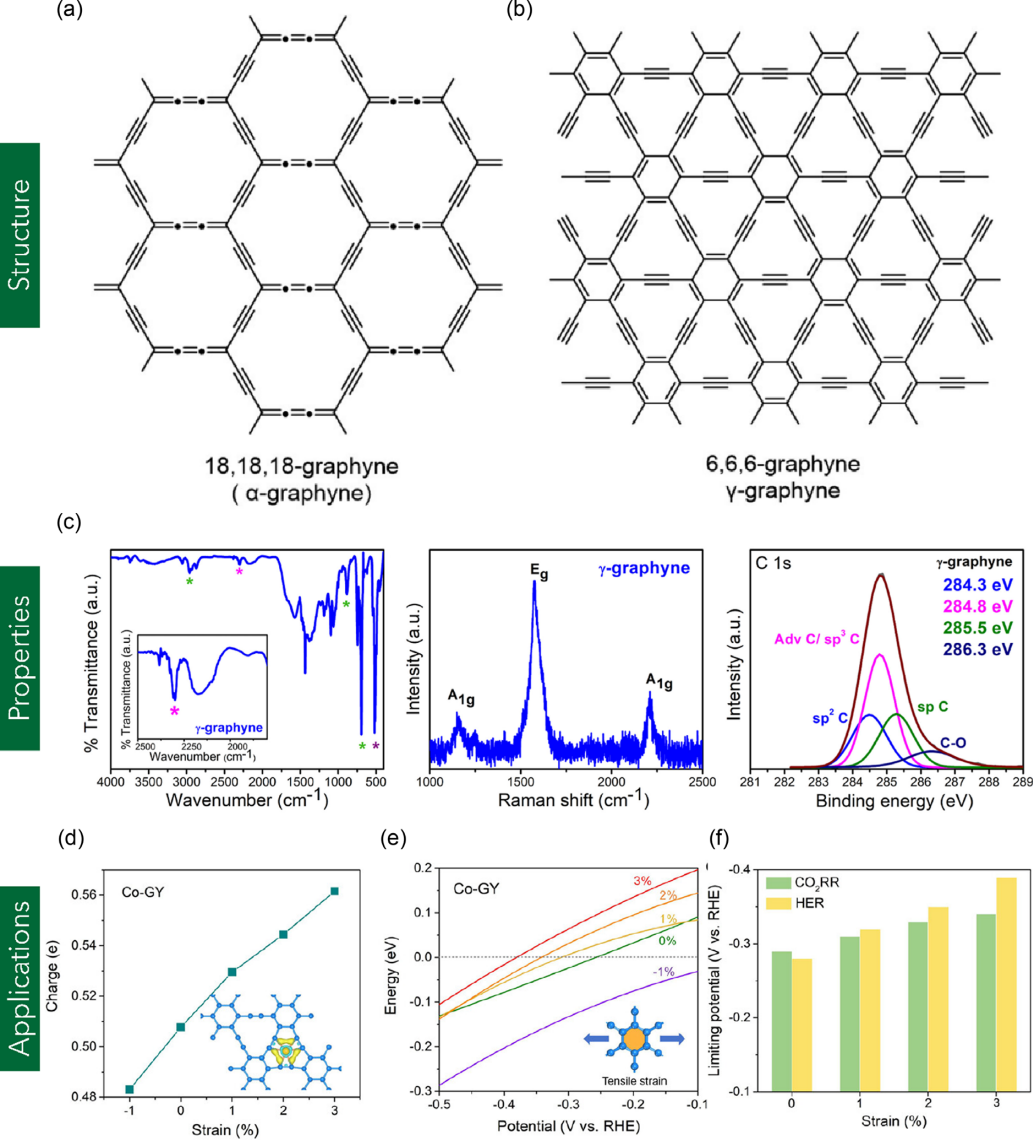
a,b) Atomic structures of α‐graphyne and γ‐graphyne. Reproduced under terms of the CC‐BY license.^[^ ^396^ ^]^ Copyright 2022, The Authors, published by MDPI. c) Characterization of γ‐graphyne synthesized via Sonogashira cross‐coupling, including Raman, FTIR, and XPS spectra. (c) is reproduced with permission.^[^ ^245^ ^]^ Copyright 2022, Elsevier Ltd. d) Total charge analysis of Co‐supported γ‐graphyne catalysts under varying biaxial strain. e) Comparative performance of γ‐graphyne‐based catalysts for CO_2_ reduction reaction (CO_2_RR) and hydrogen evolution reaction (HER). f) Limiting potentials of γ‐graphyne‐supported catalysts as a function of applied tensile strain (0%–3%). (d,f) are reproduced with permission.^[^ ^154^ ^]^ Copyright 2024, American Chemical Society.

Graphyne represents a novel class of 2D carbon allotropes characterized by a combination of sp and sp^2^ hybridized carbon atoms. Figure 1j shows the chemical structure of graphyne. Unlike graphene, which is composed entirely of sp^2^‐hybridized carbon atoms, graphyne incorporates acetylenic linkages (–C≡C–) into the carbon lattice by replacing selected C—C bonds with linear carbon chains, resulting in a diverse set of structural configurations (**Figure** **11**a,b).^[^ ^140^ ^]^ This new family of carbon materials was first theoretically proposed by Baughman et al. in the late 1980s.^[^ ^146^, ^147^ ^]^ Graphyne exhibits semiconducting behavior with a predicted bandgap of ≈0.52 eV and a binding energy of 7.95 eV atom^−1^, ≈90% of graphite's, suggesting potential thermodynamic stability if synthesized experimentally.^[^ ^146^ ^]^ The unique electronic structure arising from mixed hybridization states and acetylenic connectivity offers tunable electronic properties, making graphyne a promising candidate for future nanoelectronic and optoelectronic applications. Figure 11c shows the Raman, Fourier transform infrared spectroscopy (FTIR), and XPS spectra of γ‐graphyne. Mechanically, graphyne possesses a lower theoretical Young's modulus (532.5 GPa) compared with graphene (≈1 TPa), primarily due to the weaker sp–sp^2^ hybridized acetylene bonds compared to the all‐sp^2^ bonding network in graphene.^[^ ^148^ ^]^ Its ultimate tensile strength has been calculated as 48.2 GPa at 8.19% strain along the reclined‐chair direction and 107.5 GPa at 13.24% strain along the zigzag direction. Additionally, graphyne demonstrates an interlayer adhesion energy of 223.5 mJ m^−2^, an interlayer spacing of 3.20 Å, and a bending stiffness of 2.69 × 10^−19^ J (1.68 eV), values that are comparable to those of graphene and suitable for flexible device applications.^[^ ^149^ ^]^ Despite its relatively lower mechanical and thermodynamic stability compared with graphene, the graphyne family exhibits significant potential in gas separation, catalysis, and energy storage applications, largely due to its inherent porosity, anisotropic bonding, and tunable physicochemical properties.

Functionalization of γ‐graphyne has been extensively investigated through computational modeling and experimental approaches to tailor its electronic, chemical, and mechanical properties. Using DFT, Kim et al.^[^ ^150^ ^]^ explored the effects of transition metal (TM) doping on the structural, magnetic, and electronic properties of γ‐graphyne. The study revealed that most transition metals form stable chemical bonds with the acetylenic linkages of the graphyne lattice, except for group XII metals (which possess fully occupied d^10^ orbitals), which exhibit weak interactions. TM doping induced n‐type electronic behavior in γ‐graphyne due to the high electronegativity and charge transfer capabilities of the dopants, thereby significantly altering its electronic structure and enhancing its potential for functional device applications. Experimentally, Bolding et al.^[^ ^151^ ^]^ demonstrated edge functionalization of γ‐graphyne using two distinct chemical strategies: 1) Sonogashira cross‐coupling via brominated edge functionalities, and 2) terminal alkyne capping. These functionalizations improved both the mechanical exfoliation of γ‐graphyne (achieving delamination within 30 s under sonication) and its dispersibility in nonpolar organic solvents such as hexane and heptane. Additionally, the modified γ‐graphyne exhibited a reduced exothermic decomposition temperature (≈200 °C), indicating enhanced thermal stability. In a separate study, Xiong et al.^[^ ^152^ ^]^ employed surface functionalization of γ‐graphyne using click chemistry. γ‐Graphyne was first synthesized via an aromatic nucleophilic substitution reaction and subsequently functionalized through copper‐catalyzed azide–alkyne cycloaddition reactions with azidoferrocene and trimethylsilyl azide. This reaction formed 1,2,3‐triazole linkages on the surface, yielding a highly porous structure with a BET surface area of 788.05 m^2^ g^−1^ and a pore size distribution ranging from 0.6 to 1.3 nm, making it promising for gas storage and catalytic applications.

Although γ‐graphyne exhibits significant application potential due to its unique electronic structure, porosity, and tunable properties, most investigations to date have been limited to theoretical and computational studies. These studies have primarily explored its utility in gas separation and catalysis using advanced simulation techniques such as DFT and MD. For instance, Azizi et al.^[^ ^153^ ^]^ evaluated the gas separation performance of a model graphyne‐3 membrane for mixtures containing H_2_, CO_2_, CH_4_, and C_2_H_6_ using a combination of DFT and MD simulations. Their findings revealed that graphyne‐3 exhibited an exceptionally high H_2_ permeance of 10^7^ gas permeation units, indicating excellent suitability for hydrogen separation. Furthermore, the membrane demonstrated superior H_2_/CH_4_ selectivity, with values reaching 11.25 and 8.14 for rigid and deformable graphyne‐3 configurations, respectively, underscoring the material's potential for selective gas filtration. In a separate study, Liu et al.^[^ ^154^ ^]^ explored the catalytic performance of γ‐graphyne‐supported cobalt (Co) systems for the electrochemical reduction of CO_2_. Utilizing constant‐potential DFT calculations and MD simulations, they reported a low limiting potential of –0.29 V for the Co/γ‐graphyne catalyst, which outperformed comparable Ir‐ and Rh‐supported γ‐graphyne systems. Moreover, catalytic selectivity was further enhanced through strain engineering. Applying a 3% biaxial strain weakened the binding energy of hydrogen intermediates (*H), effectively suppressing the competing hydrogen evolution reaction (HER) and promoting CO_2_ reduction (Figure 11df). The future research directions for γ‐graphyne include the development of scalable and controlled synthesis methods to enable its integration into applications requiring semiconducting, optoelectronic, and catalytic functionalities. In addition, Figure 1f illustrates the scheme of vertical/3D graphene.

#### 2.3.3 GO

**Figure 12:**
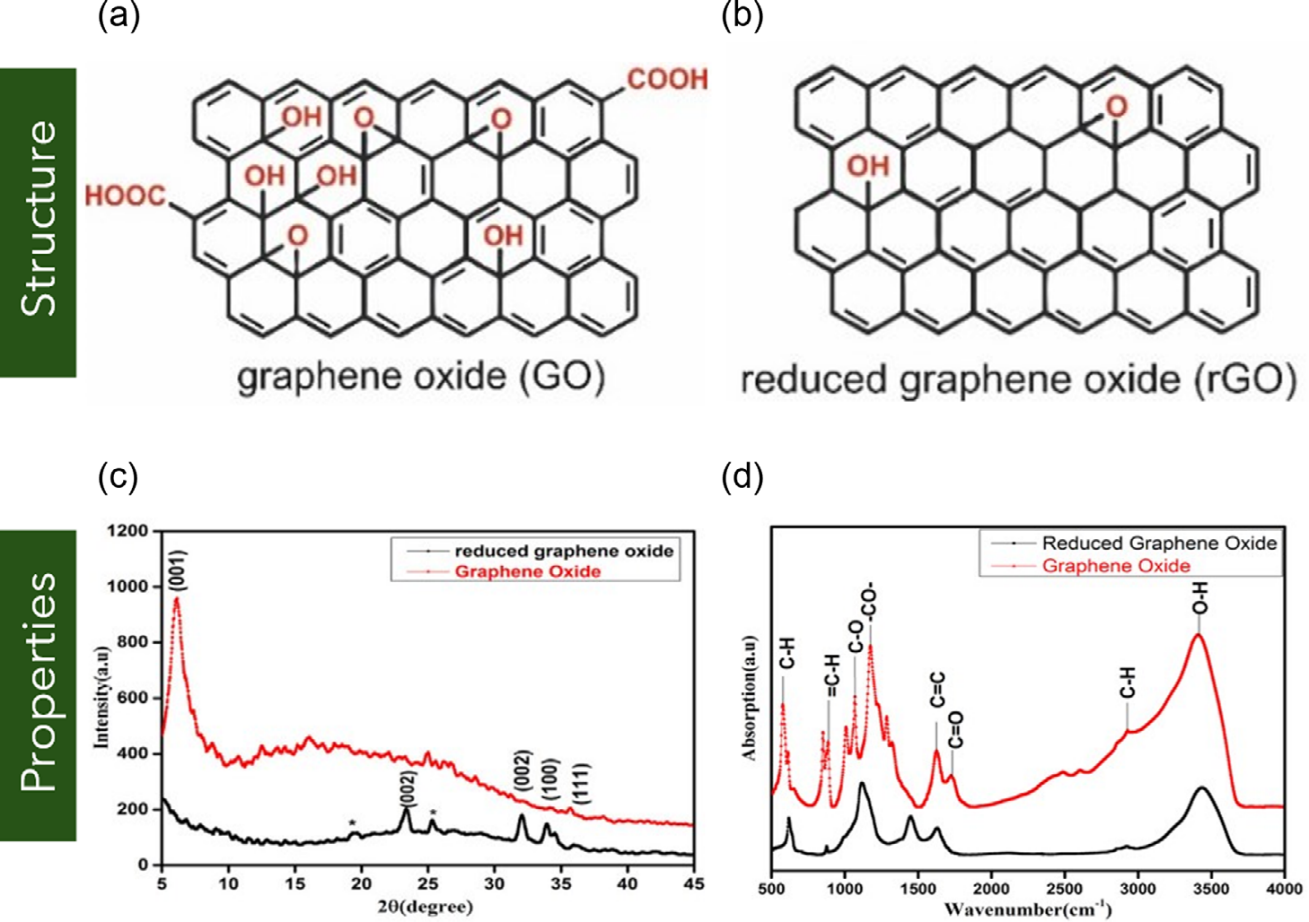
a,b) Schematic representations of GO and RGO. Reproduced under terms of the CC‐BY license.^[^ ^397^ ^]^ Copyright 2024, The Authors, published by MDPI. c,d) Comparative XRD and FTIR spectra of GO and RGO. Reproduced with permission.^[^ ^398^ ^]^ Copyright 2017, WILEY‐VCH Verlag GmbH.

GO is one of the most widely studied derivatives of graphene, formed by the oxidation of monolayer graphene—an atomically thin sheet of sp^2^‐hybridized carbon atoms arranged in a 2D honeycomb lattice.^[^ ^155^ ^]^ Figure 1i depicts the chemical structure of GO. Unlike pristine MG, which consists exclusively of sp^2^‐bonded carbon atoms, GO incorporates a variety of oxygen‐containing functional groups, including hydroxyl (—OH), carboxyl (—COOH), epoxy (—C—O—C—), and carbonyl (—C=O) groups, distributed across both its basal planes and edges.^[^ ^156^ ^]^ The representative molecular structures of GO and its reduced form, RGO, are illustrated in **Figure** **12**a,b. The introduction of these oxygen functionalities imparts hydrophilic characteristics to GO, significantly enhancing its dispersibility in aqueous and organic solvents—a property not typically observed in hydrophobic MG.^[^ ^157^ ^]^ Structurally, GO exhibits a heterogeneous morphology comprising both sp^2^‐hybridized graphitic domains and sp^3^‐hybridized carbon atoms covalently bonded to oxygen species. Hydroxyl and epoxy groups are predominantly located on the basal plane, whereas carboxyl and carbonyl groups are primarily situated along the sheet edges.^[^ ^155^, ^156^ ^]^ This non‐uniform distribution of functional groups results in a partially disordered structure, characterized by coexisting ordered (graphitic) and disordered (oxidized) regions. Consequently, GO possesses a greater interlayer spacing and thickness relative to MG, with a monolayer GO sheet typically measuring ≈1.1–1.4 nm in thickness, compared with 0.34 nm for pristine graphene.^[^ ^158^ ^]^ Figure 12c,d presents comparative X‐ray diffraction (XRD) and FTIR spectra of GO and RGO, further confirming the structural and chemical differences between the two forms.

GO exhibits a distinct set of chemical, electrical, mechanical, and thermal properties due to the presence of oxygen‐containing functional groups on its surface and edges. Chemically, GO typically contains 20–30 wt% oxygen, which significantly enhances its solubility and dispersion in polar solvents, thereby enabling solution‐based processing techniques not feasible with pristine graphene.^[^ ^159^ ^]^ The carbon‐to‐oxygen (C/O) atomic ratio in GO can be tuned by varying the degree of oxidation, typically ranging from 2:1 to 8:1, depending on synthesis conditions. Electrically, GO behaves as an insulator due to the disruption of its conjugated π‐electron system by sp^3^‐hybridized carbon–oxygen bonds. It exhibits a high sheet resistance on the order of 10^12^ Ω sq^−1^. However, partial reduction of the oxygen functionalities restores electrical conductivity by re‐establishing sp^2^ domains. RGO can achieve electrical conductivities in the range of 1–2 × 10^3^ S m^−1^, depending on the extent of reduction and restoration of the conjugated network.^[^ ^160^ ^]^ Mechanically, monolayer GO exhibits a tensile strength of ≈120–150 MPa and a Young's modulus in the range of 200–250 GPa—values that are significantly lower than those of monolayer graphene, which has a Young's modulus of ≈1 TPa and exceptional tensile strength.^[^ ^161^ ^]^ Nonetheless, GO's functional groups allow for strong interfacial interactions with polymer matrices, making it an effective reinforcement material in composite systems. Thermally, GO begins to decompose at relatively low temperatures (150–200 °C), with the breakdown of oxygen functionalities releasing gases such as CO and CO_2_. Complete thermal reduction of GO typically requires treatment above 500 °C under inert or reducing atmospheres. At elevated temperatures (≈1000 °C), the oxygen content is substantially removed, yielding a C/O ratio as high as 14:1 and significantly enhancing electrical conductivity due to the restoration of extended sp^2^ domains.^[^ ^162^, ^163^ ^]^ With the addition of oxygen species in the structure, elastic modulus of GO can decrease as a function of oxygen content. For GO, Hou et al.^[^ ^164^ ^]^ derived an analytical relationship (Equation 4) between stress, strain, and oxygen functionalization as

(4) $$\Sigma \;=\;\left(E\;-\;K_{1}\varphi \right)\;\eta \;+\;\left(D\;+\;K_{2}\varphi \right)\;\eta^{2}$$

here, the $K_{1}$, $K_{2}$ are the correction factors associated with the oxygen content, *φ* is the relative content of epoxy groups, *E* is the Young's modulus, *D* is the third‐order nonlinear elastic modulus of MG, Σ is the second Piola–Kirchhoff stress, and *η* is the Lagrangian strain. This relation indicates that the Young's modulus decreases linearly with oxygen content.

GO has been extensively explored in diverse applications, including energy storage, sensing, and biomedical technologies, due to its tunable surface chemistry, high surface area, and excellent dispersibility. In the context of energy storage, Ambrosi et al.^[^ ^165^ ^]^ synthesized GO via electrochemical exfoliation and evaluated its performance in supercapacitor systems. The electrochemical properties of GO were examined in different electrolyte media. GO prepared in Na_2_SO_4_ exhibited the highest specific capacitance of 106 F g^−1^ at a current density of 0.1 A g^−1^, outperforming GO synthesized in LiClO_4_ (78 F g^−1^) and H_2_SO_4_ (21 F g^−1^). Additionally, the electrochemically derived GO demonstrated a high heterogeneous electron transfer rate constant (k_0__obs) of 6.1 × 10^−3^ cm s^−1^, underscoring its suitability for energy storage applications. For sensing applications, Robinson et al.^[^ ^166^ ^]^ developed a molecular sensor based on GO to detect hydrogen cyanide (HCN). The GO‐based sensors exhibited exceptional sensitivity, capable of detecting molecular adsorbates at parts‐per‐billion (ppb) concentrations. A key advantage of GO sensors over single‐walled CNT (SWCNT) sensors was their significantly reduced (by 10−100 fold) 1/f noise,^[^ ^167^, ^168^ ^]^ which enhances signal stability and accuracy. Moreover, the sensor's response characteristics could be tuned by modulating the reduction level of GO. Extended exposure to hydrazine (N_2_H_4_) vapor decreased the oxygen defect density by restoring sp^2^ domains, leading to faster, reversible sensor responses and reduced slow, irreversible interactions associated with high‐energy defect sites.

GO can be chemically and electrochemically functionalized through the incorporation of metal atoms (e.g., Ni, Fe) and heteroatoms (e.g., N, O) to modulate its electronic, structural, and transport properties. These doping strategies enable precise tuning of GO's conductivity, carrier mobility, and chemical reactivity for various electronic and catalytic applications. For instance, Chae et al.^[^ ^169^ ^]^ demonstrated the electrochemical doping of GO with nickel (Ni) atoms. The intercalated Ni introduced p‐type doping compensation, which significantly altered the electronic behavior of GO‐based FETs. Specifically, electron mobility decreased from 1.40 to 0.12 cm^2^ V^−1^ s^−1^, and the transfer characteristics shifted from ambipolar to unipolar p‐type. Additionally, the sheet resistance of the doped GO films was reduced by ≈50%, indicating enhanced conductivity resulting from Ni incorporation. Such changes in carrier concentration and bonding states can influence load transfer efficiency and defect evolution under stress. Enhanced conductivity often correlates with improved sp^2^ network restoration, which stabilizes the lattice and increases in‐plane stiffness. In another study, Li et al.^[^ ^170^ ^]^ developed a scalable chemical route for synthesizing nitrogen‐doped GO (N‐GO) via thermal annealing under an NH_3_ atmosphere. Nitrogen incorporation was initiated at temperatures as low as 300 °C, with a maximum doping level of ≈5 at.% N achieved at 500 °C. The N‐doping process was also accompanied by simultaneous thermal reduction, as evidenced by a significant decrease in oxygen content from ≈28% in as‐prepared GO to ≈2% after annealing at 1100 °C in NH_3_. This dual effect of doping and reduction enabled the formation of conductive, nitrogen‐rich graphene oxide suitable for electronic and catalytic applications. The reduction in oxygen content not only improves conductivity but also eliminates mechanically weak epoxide and hydroxyl groups, leading to improved structural integrity and higher elastic modulus. Nitrogen incorporation further introduces localized strain and modified bonding configurations, which can enhance interfacial adhesion in composites and contribute to improved toughness and fracture resistance.

### 2.4 Based on Composite System

#### 2.4.1 Graphene Nanocomposites

**Figure 13:**
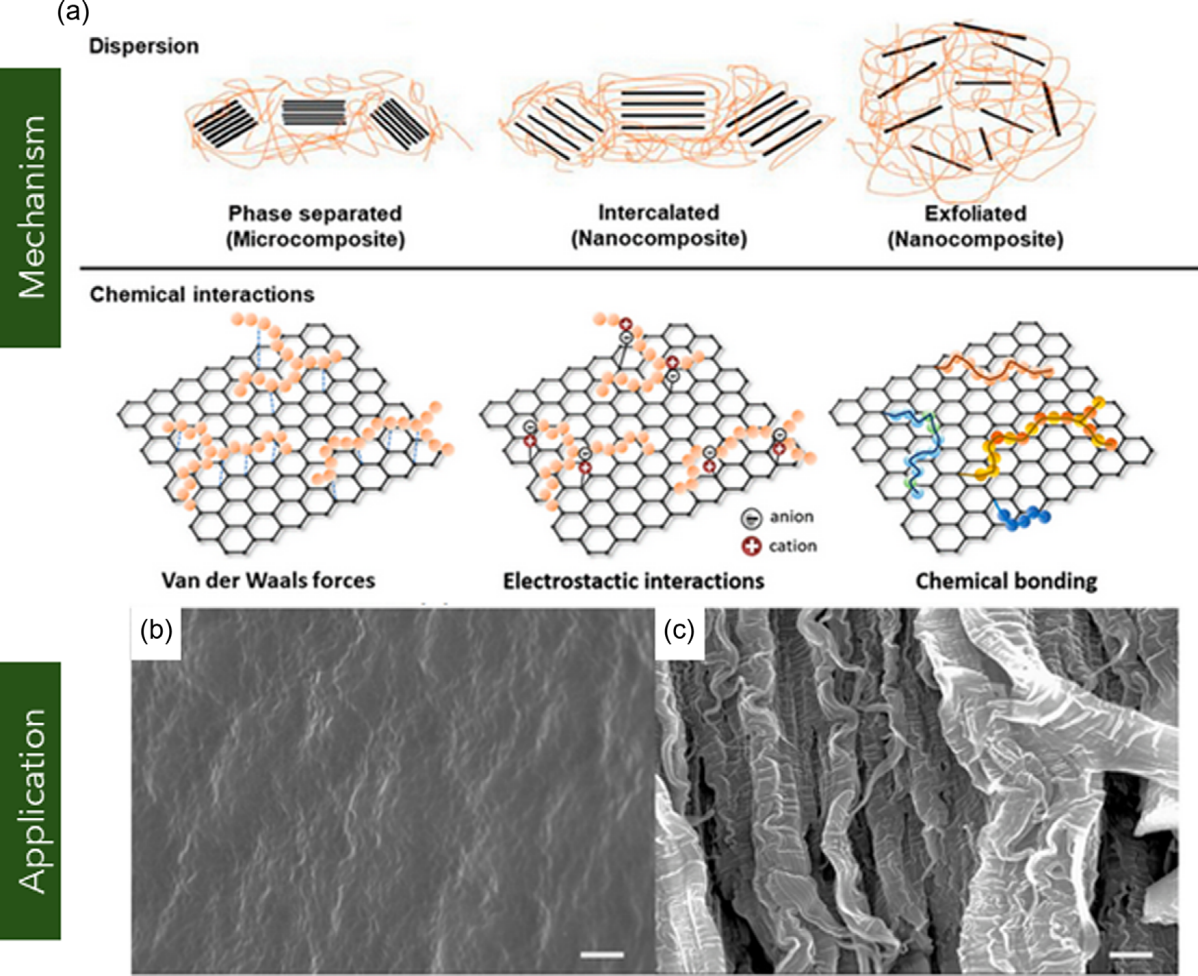
a) Diagram illustrating the dispersion mechanisms and interfacial interactions between graphene sheets and polymer chains in the formation of graphene‐based nanocomposites. Reproduced under the CC‐BY license.^[^ ^399^ ^]^ Copyright 2020, The Authors, published by MDPI. Reproduced with permission.^[^ ^400^ ^]^ Copyright 2018, Elsevier Ltd. b,c) SEM images of heptane‐functionalized GNPs/LLDPE nanocomposites, showing morphology and dispersion of HGN within the polymer matrix. Reproduced with permission.^[^ ^180^ ^]^ Copyright 2020, Elsevier Ltd.

Graphene nanocomposites represent a class of advanced hybrid materials that incorporate graphene or its derivatives into various host matrices, including polymer‐based composites,^[^ ^171^ ^]^ metal matrix composites (MMCs),^[^ ^172^ ^]^ ceramic matrix composites (CMCs),^[^ ^173^ ^]^ or C–C composites.^[^ ^174^ ^]^ Figure 1k illustrates the schematic of graphene nanocomposites. These nanocomposites are structurally characterized by the uniform dispersion of graphene sheets or platelets within the matrix phase, which significantly enhances the composite's mechanical strength, thermal conductivity, and electrical performance.^[^ ^175^ ^]^ **Figure** **13**a illustrates the interaction mechanisms between graphene and polymer chains. The specific enhancements depend heavily on the type of graphene variant employed—such as GO, rGO, or chemically functionalized graphene—each offering distinct interfacial compatibility and functional properties. For instance, Cheng‐an et al.^[^ ^176^ ^]^ incorporated 20 wt% GO into a PVA matrix, resulting in a fivefold improvement in tensile strength compared with neat PVA, achieving a value of 59.6 MPa. In another example, Pitiphattharabun et al.^[^ ^176^ ^]^ synthesized RGO/ZnO nanocomposites via a hydrothermal method for the electrochemical detection of acetylcholine. The integration of RGO significantly broadened the sensor's detection range from 0.1 to 1000 μM and improved its sensitivity, achieving values between 1.56 × 10^−2^ and 5.90 × 10^−5^ μA μM^−1^ mm^−2^. Additionally, Chen et al.^[^ ^177^ ^]^ developed a Nb_2_CT_*x*_/N‐doped RGO (NrGO) hybrid composite using a wet chemical synthesis route. The incorporation of functionalized graphene notably enhanced the electrocatalytic activity of the composite, enabling the sensitive and selective detection of Fluoxetine (FLX) over a wide linear concentration range of 1.0–10 μM with a low LOD of 0.34 μM.

Graphene nanocomposites exhibit remarkable versatility and have been applied across a wide range of fields, including energy storage, catalysis, and gas sensing, owing to their exceptional electrical conductivity, high surface area, and tunable interfacial properties. In the domain of energy storage, Sardana et al.^[^ ^178^ ^]^ fabricated a flexible supercapacitor by assembling nitrogen‐doped graphene and PANI onto a carbon cloth substrate. The resulting PANI/N‐doped graphene hydrogel‐coated composite exhibited a high specific capacitance of 808.7 F g^−1^, along with a power density of 0.44 kW kg^−1^ and an energy density of 13.63 Wh kg^−1^, demonstrating excellent electrochemical performance for wearable energy storage devices. For catalytic applications, Mayani et al.^[^ ^179^ ^]^ developed a strontium ferrite/graphene (SRG) nanocomposite via hydrothermal synthesis for the degradation of organic dyes such as Eosin‐Y and Orange (II) in aqueous media. The SRG catalyst demonstrated high degradation efficiencies of 96.2% and 98.7% for Eosin‐Y and Orange (II), respectively, under mild conditions. Additionally, the material exhibited recyclability, supporting its potential for practical wastewater treatment applications. In structural composites, Song et al.^[^ ^180^ ^]^ prepared a heptane‐functionalized graphene nanoplatelet (HGN)‐reinforced linear low‐density polyethylene (LLDPE) nanocomposite using a ball milling process. At a loading of 5 wt% HGN, the composite exhibited significant improvements in mechanical properties, including tensile strength (25.3 ± 1.5 MPa), yield strength (9.9 ± 0.9 MPa), and Young's modulus (189.4 ± 4.6 MPa), highlighting its suitability for high‐strength polymer applications. The morphology of the composite, as visualized via scanning electron microscope (SEM), is shown in Figure 13b,c. For gas sensing, Seekaew et al.^[^ ^181^ ^]^ synthesized a Ti_3_C_2_T_*x*_ MXene/graphene oxide (GO)/CuO/ZnO hybrid nanocomposite via a hydrothermal method for ammonia (NH_3_) detection at room temperature. The sensor exhibited a high response of 96% at 200 ppm NH_3_ and an ultralow detection limit of 4.1 ppm. Moreover, the nanocomposite demonstrated excellent selectivity toward various VOCs, including methanol, ethanol, and acetone, indicating its promise for environmental monitoring and safety applications.

MG exhibits a zero‐bandgap, which limits its direct application in digital electronics. To overcome this limitation, various graphene‐based nanostructures have been explored, each introducing mechanisms to open a tunable bandgap. For instance, AB‐stacked BLG can exhibit a band gap up to 250 meV under a perpendicular electric field due to inversion symmetry breaking,^[^ ^182^ ^]^ allowing electronic modulation while retaining high carrier mobility. With increasing layer number, bandgap tunability diminishes because of interlayer screening; however, stacking‐dependent electronic states in few‐layer graphene (FLG, 3 layers) can still provide a moderate band gap of ≈0.23 eV.^[^ ^183^ ^]^

PG, although like MG in principle, contains grain boundaries that locally disrupt the π‐electron network. These defects induce quantum confinement and transport gaps, producing a tunable band gap up to 1.04 eV,^[^ ^184^ ^]^ effectively enabling semiconducting behavior in otherwise metallic graphene. GO achieves bandgap opening through chemical functionalization. Oxygen‐containing groups break π‐conjugation, with the band gap ranging from 2 to 0.02 eV, depending on the reduction level.^[^ ^185^ ^]^ Hence, the reduction level allows controlled transition from insulating to semiconducting states.

Other carbon allotropes, such as graphyne, inherently possess a bandgap (0.094–0.226 eV) owing to the combination of sp‐ and sp^2^‐hybridized carbon atoms,^[^ ^186^ ^]^ providing a structurally distinct route to electronic modulation. On the other hand, GNRs demonstrate an optical band gap ranging from 1.09 to 1.21 eV,^[^ ^187^ ^]^ where quantum confinement along the ribbon width and edge topology (armchair vs. zigzag) inversely modulate the band gap. As a result, it enables precise control of electronic properties at the nanoscale.

Overall, these graphene‐based nanostructures mitigate the intrinsic zero bandgap of MG through mechanisms including electric‐field‐induced symmetry breaking, defect‐induced quantum confinement, chemical functionalization, hybridized bonding networks, and geometric confinement for this reason by enabling tunable semiconducting behavior suitable for electronic and optoelectronic applications.

This section provides an overview of the structural, physical, thermal, and electrical properties of various graphene nanostructures and their corresponding composites, along with their diverse applications. The subsequent section will focus on their fabrication methods and processing techniques.

## 3 Synthesis of Graphene Nanostructures

**Figure 14:**
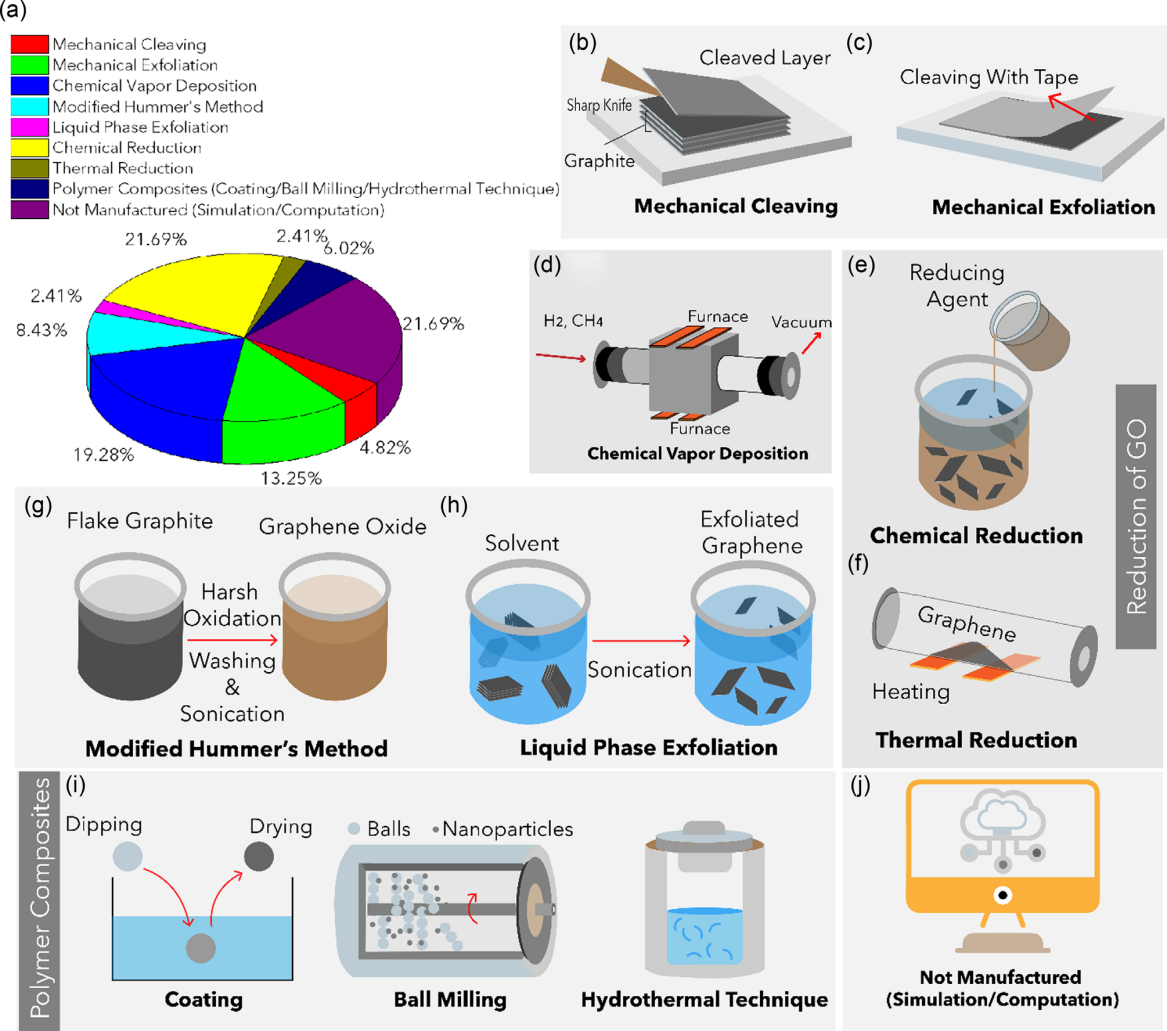
a) Pie chart illustrates the distribution of fabrication techniques employed for synthesizing graphene variants used in mechanical property evaluation. b) Micromechanical cleavage. c) Mechanical exfoliation. d) CVD. e) Chemical reduction of GO. f) Thermal reduction of GO. g) Modified Hummers’ method. h) LPE. i) Polymer composites. j) Computational modeling and simulation approaches.

To enable the multiscale structural and mechanical characterization of graphene nanostructures, various fabrication techniques are employed, including micromechanical cleavage, mechanical exfoliation, LPE, and CVD. Each method yields graphene with distinct structural and chemical characteristics, such as variations in defect density, layer thickness, crystallinity, and surface functionalization. For instance, CVD‐grown graphene typically exhibits low defect densities and high crystallinity, whereas LPE‐ or oxidation/reduction‐derived graphene often contains a higher concentration of structural defects and surface irregularities. Consequently, the fabrication method significantly influences the mechanical performance of the resulting graphene variants. **Figure** **14** provides an overview of the fabrication techniques used for preparing graphene nanostructures. Figure 14a depicts the pie chart of fabrication techniques employed for synthesizing graphene variants used in mechanical property evaluation.

### 3.1 Mechanical Cleaving and Exfoliation

Graphene layers can be isolated from bulk graphite through mechanical cleaving, a process that applies macroscopic forces along graphite's weakly bonded basal planes. This method exploits the intrinsic anisotropy of graphite, wherein the strong in‐plane sp^2^ covalent bonds (≈16 eV atom^−1^) contrast sharply with the relatively weak interlayer vdW interactions (≈0.3 eV atom^−1^).^[^ ^188^ ^]^ Due to this disparity, cleaving along the basal planes is significantly easier than across them. Tools such as blades or even fingernails can disrupt these weak interlayer forces, separating graphene layers. Figure 14b,c illustrates the scheme of mechanical cleaving and exfoliation. However, mechanical cleaving generally yields relatively thick multilayer graphene fragments, typically comprising three to five layers. In contrast, mechanical exfoliation is specifically designed to isolate MG or FLG with minimal disruption to the basal plane, enabling the production of high‐quality graphene with low defect densities.^[^ ^189^ ^]^ The pioneering demonstration of this method was reported in 2004, wherein MG was successfully extracted from HOPG using the now well‐known “Scotch tape” method.^[^ ^190^ ^]^ In this approach, a mesa of HOPG was first cleaved from the bulk material, followed by repeated exfoliation using adhesive tape to isolate ultrathin graphene layers. Despite its ability to produce pristine graphene flakes, the technique is inherently time‐consuming, relies on the applied normal force during exfoliation, and is not scalable—limiting its applicability to fundamental research and laboratory‐scale studies.

To address the labor‐intensive nature of conventional mechanical cleaving followed by manual exfoliation, Jayasena et al.^[^ ^191^ ^]^ developed a lathe‐assisted exfoliation technique for producing graphene flakes from mechanically cleaved HOPG. In this method, HOPG was embedded in a pyramid‐shaped epoxy mold, and an ultrasharp diamond wedge—mounted on an ultrasonic oscillation system—was precisely aligned with the HOPG block. The wedge acted as a peeling tool, facilitating the controlled removal of graphene layers. This approach yielded graphene flakes with thicknesses ranging from 10 to 20 nm.^[^ ^191^ ^]^ In subsequent work, Jayasena et al.^[^ ^189^ ^]^ employed MD simulations to estimate the exfoliation forces associated with this wedge‐based method. At a temperature of 300 K, the exfoliation force peaked at ≈13.5 eV Å^−1^ for single‐layer graphene and 15 eV Å^−1^ for triple‐layer cleaving. During steady‐state exfoliation, the force plateaued at ≈1 eV Å^−1^, corresponding closely to the energy required to overcome van der Waals interactions between layers.^[^ ^189^ ^]^ In addition to the wedge‐based strategy, mechanical exfoliation has also been achieved using a three‐roll mill setup. For instance, Chen et al.^[^ ^192^ ^]^ employed polyvinyl chloride (PVC) as an adhesive coating on the rollers. As the rollers compressed and sheared the graphite, they mimicked the function of adhesive tape, facilitating the exfoliation of graphene nanosheets through applied normal forces. While these methods offer potential for scalable graphene production, they necessitate subsequent purification steps to remove residual adhesive and isolate high‐purity graphene sheets.

The mechanism of mechanical exfoliation is fundamentally governed by the interplay between interlayer vdW interactions and the application of external shear or normal forces. In graphite, the vdW binding energy is ≈43 meV atom^−1^, translating to an exfoliation energy of ≈0.3 J m^−2^. As reported by Jacob et al.^[^ ^193^ ^]^ the feasibility of exfoliating a 2D material is primarily determined by its interlayer adhesion strength (*w*). The vdW interaction energy between adjacent layers can be expressed as shown in Equation (5).

(5) $$w=-\frac{A}{12\pi D^{2}}$$

where *D* is the interlayer distance and *A* is the Hamaker constant, which is dependent on the vdW forces and atomic density. While the corresponding effective vdW force ($f_{\text{ab}}$) is described by Equation (6).

(6) $$f_{\text{ab}}=-\frac{A}{6\pi D^{3}}$$

The cohesion energy reflects the energy required to overcome these interlayer forces, which depends on the equilibrium spacing between graphene layers. However, the effective cohesive force during exfoliation is pathway‐dependent, influenced by interfacial separation and shear loading. The critical peeling force required to delaminate adjacent graphene layers can be approximated using Equation (7).

(7) $$f_{\text{peel off}}=f_{\text{ab}}\frac{D}{2d}$$

where *d* is the distance between upper and lower layer and *f*_peel off_ is the per unit area force required to separate during the mechanical exfoliation. Notably, the peeling force is substantially higher than the effective vdW forces, enabling successful layer separation. To quantify exfoliation energetics, several computational studies have been performed. For instance, Kabengele et al.^[^ ^194^ ^]^ employed DFT to compute exfoliation energy of 18.5 meV Å^−1^ for graphene, underscoring the influence of polarized bonds in enhancing interlayer adhesion strength.

**Figure 15:**
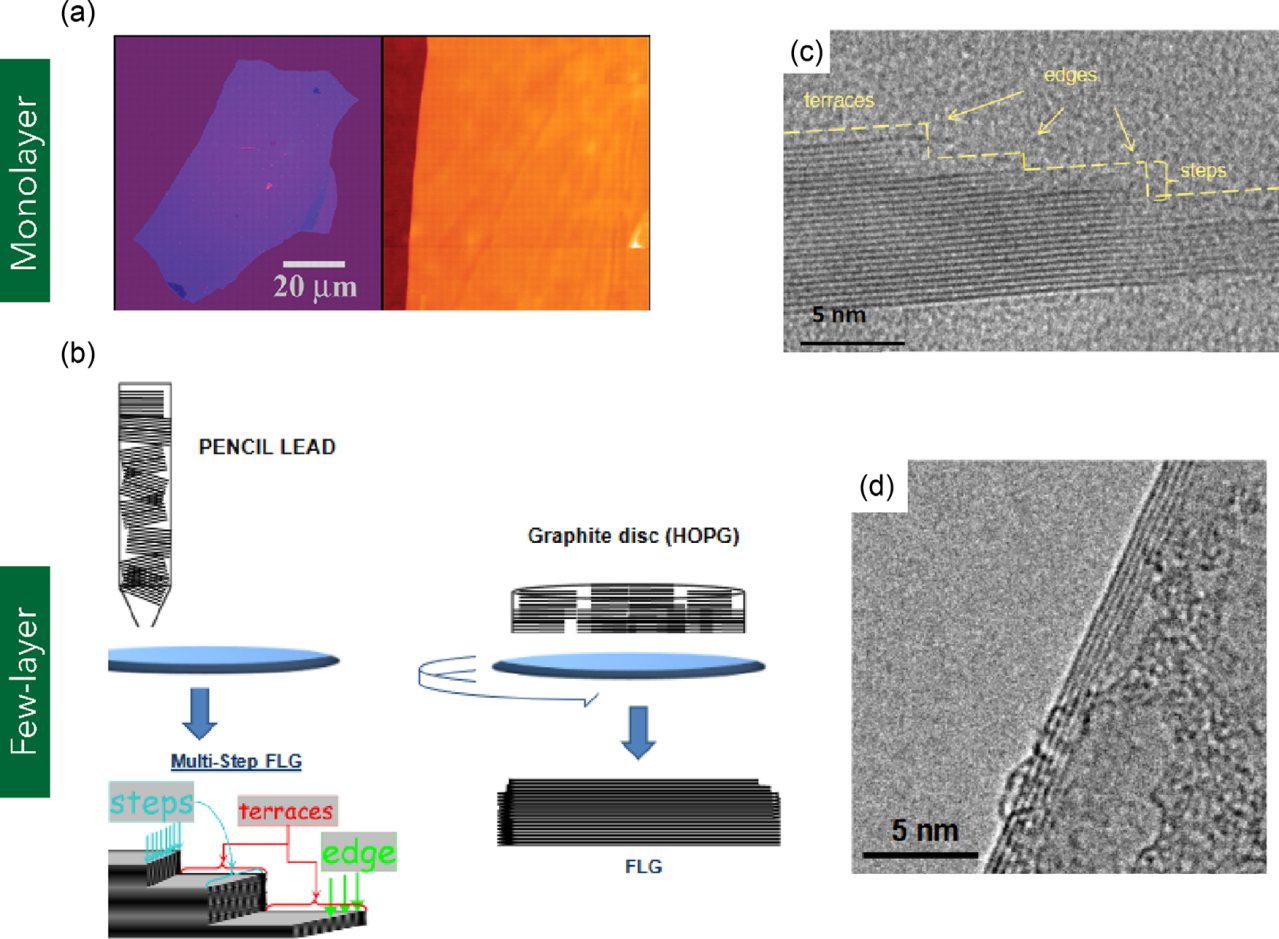
a) Optical micrograph of the first isolated MLG flake alongside corresponding AFM characterization. Reproduced with permission.^[^ ^29^ ^]^ Copyright 2002, Science. b) Schematic representation of the mechanical exfoliation process used to produce FLG from a graphite disc. c,d) TEM images of exfoliated FLG, showing a multilayer morphology comprising three to seven layers. (c,d) are reproduced under terms of the CC‐BY license.^[^ ^196^ ^]^ Copyright 2019, The Authors, published by MDPI.

Using such a technique, several graphene nanostructures have been synthesized. Taking MG as an example, the first successful isolation of MG was achieved via mechanical exfoliation—commonly referred to as the “Scotch tape method”—which involved repeated peeling of highly ordered pyrolytic graphite (HOPG) to yield single‐, bi‐, tri‐, and few‐layer graphene flakes with lateral dimensions of up to ≈10 μm (**Figure** **15**a).^[^ ^29^, ^191^ ^]^ Despite a relatively low yield (<10%)^[^ ^34^, ^195^ ^]^ and the presence of adhesive contaminants, this method remains widely used due to its simplicity and ability to produce structurally pristine graphene with high reproducibility. Pirzado et al.^[^ ^196^ ^]^ utilized a mechanical exfoliation technique, involving a glass surface‐assisted ablation process to delaminate FLG from HOPG, yielding FLG with fewer than 10 layers. Figure 15b shows that the exfoliation process of FLG. TEM images (Figure 15c,d) confirmed the multilayer structure, while Raman spectroscopy revealed a lower D/G intensity ratio of 0.13, indicating reduced defect density and higher carbon purity.^[^ ^196^ ^]^

In terms of graphene drum fabrication, it typically involves a two‐step process: 1) preparation of a substrate with patterned microcavities and 2) transfer of pre‐fabricated mono‐ or multilayer graphene onto the cavities, followed by stabilization treatments. The most commonly employed methods for producing graphene drums are CVD and mechanical exfoliation. For example, Yin et al.^[^ ^124^ ^]^ developed graphene drums using a modified mechanical exfoliation approach combined with thermal annealing (ranging from 50 to 150 °C). Initially, micrometer‐scale holes were patterned onto a SiO_2_/Si(100) substrate via lithography techniques. Exfoliated graphene nanosheets were then transferred onto these cavities and subjected to annealing, resulting in drum‐like suspended membranes. The fabricated graphene drums exhibited a maximum tensile strain of 1.3% and adopted a parabolic curvature with a central deflection of 57.4 nm across a span of 4 μm.

### 3.2 CVD

CVD is a widely utilized technique for synthesizing thin films and surface coatings, including monolayer and few‐layer graphene, through the thermal decomposition of gaseous precursors on heated metal substrates (Figure 14d). In a standard CVD configuration, a metal substrate—commonly Cu, Ni, or iron (Fe)—is placed within a high‐temperature furnace and exposed to controlled flows of reactive gases such as CH_4_ and hydrogen (H_2_).^[^ ^197^ ^]^ Hydrocarbon gases undergo pyrolysis at elevated temperatures, typically above 1000 K, releasing carbon atoms that adsorb onto the substrate surface. These carbon species nucleate and assemble into graphene domains, eventually forming continuous films. The substrate plays a dual role: serving as a deposition platform and acting as a catalytic interface to facilitate carbon atom rearrangement and graphene crystallization.^[^ ^198^ ^]^ Key processing parameters—including temperature, precursor gas composition and flow rate, pressure, and substrate type—critically influence film thickness, uniformity, defect density, and overall material quality.^[^ ^175^ ^]^ The choice of substrate is particularly significant in modulating the nucleation and growth dynamics. CVD growth is typically carried out on TMs that lower the energy barrier for graphene formation via catalytic decomposition of CH_4_. Among these, Cu is frequently favored due to its exceptionally low carbon solubility (≈7.4 ± 0.5 atomic ppm at 1293 K),^[^ ^199^ ^]^ which promotes surface‐mediated growth and enables the synthesis of high‐quality MG. In contrast, Ni—although widely used due to its strong catalytic activity—tends to produce multilayer graphene because of its higher carbon solubility and subsequent bulk precipitation effects during cooling.^[^ ^200^, ^201^, ^202^ ^]^

**Figure 16:**
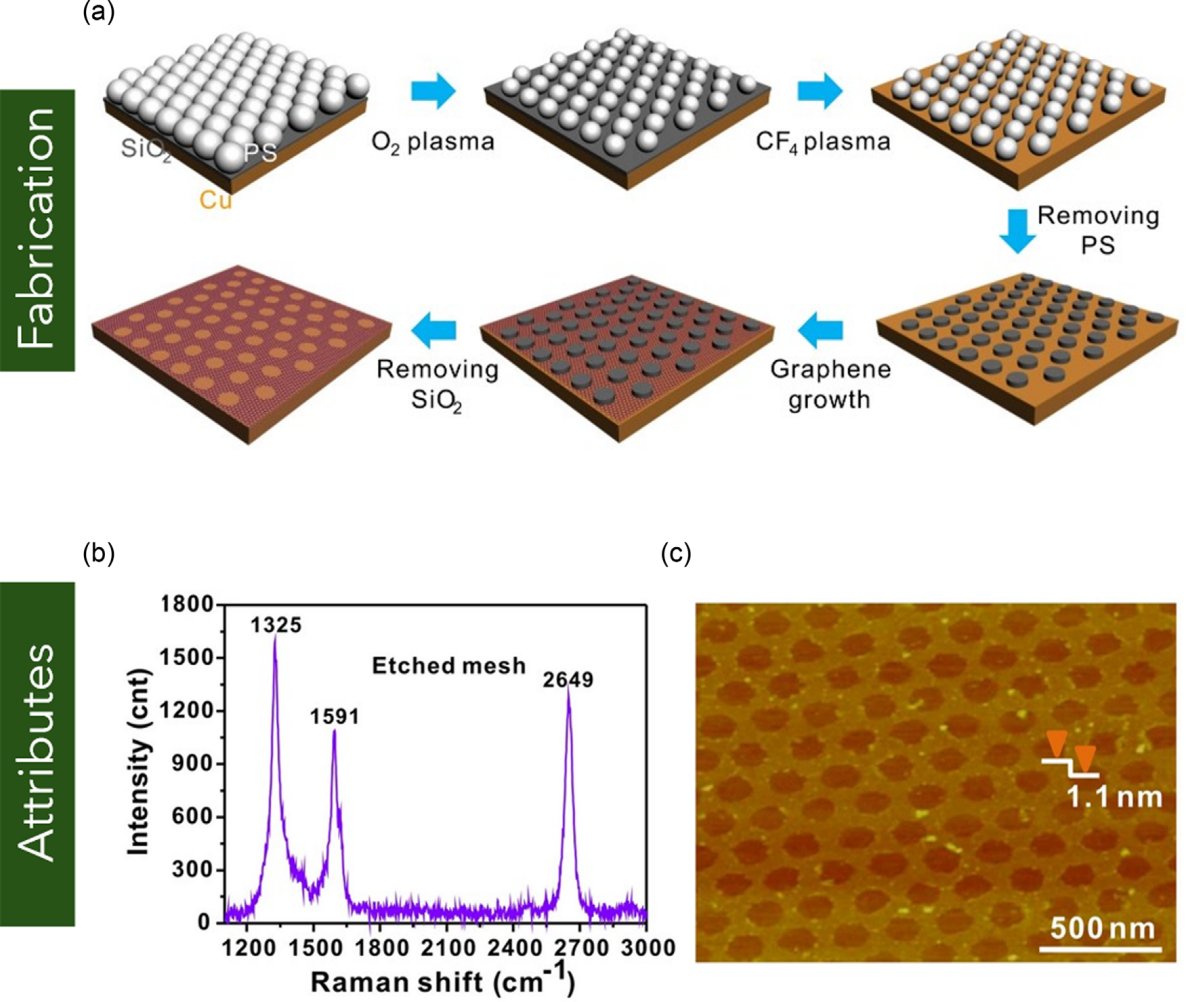
a) Schematic representation of the direct CVD growth of GNM on PS‐modified Cu foil using nanosphere lithography. b) Raman spectra of the GNM following transfer onto a SiO_2_/Si substrate, confirming characteristic graphene features. c) AFM height profile of the as‐grown GNM, indicating a uniform thickness of ≈1.1 nm, consistent with MG. Reproduced under the terms of the CC‐BY license.^[^ ^197^ ^]^ Copyright 2013, The Authors, published by Nature Portfolio.

Graphene growth on polycrystalline Ni substrates is primarily governed by the material's intrinsic carbon solubility and the precipitation behavior during post‐deposition cooling. At elevated temperatures ranging from 900 to 1000 °C, Ni exhibits relatively high carbon solubility, enabling the dissolution of carbon atoms into the bulk lattice when exposed to hydrocarbon precursors such as CH_4_.^[^ ^203^ ^]^ This leads to the formation of a solid solution phase. Upon cooling, the solubility of carbon in Ni decreases exponentially, prompting carbon segregation to the surface and subsequent precipitation as graphene layers. The cooling rate plays a critical role in determining the resulting graphene morphology. Intermediate cooling rates (≈10 °C s^−1^) are optimal for controlled carbon segregation, often yielding FLG with uniform coverage. In contrast, faster cooling rates (≈20 °C s^−1^) reduce the time available for surface diffusion and reorganization, thereby promoting multilayer graphene (MLG) formation.^[^ ^204^ ^]^ Among Ni crystal orientations, the (111) facet—with a hexagonal lattice constant of 2.49 Å closely matching that of graphene (2.46 Å)—facilitates epitaxial alignment, favoring monolayer formation and improved structural coherence.^[^ ^205^ ^]^ However, polycrystalline Ni films are susceptible to grain boundary effects, where discontinuities in crystallographic orientation may act as preferential sites for multilayer nucleation, compromising film uniformity. In contrast, Cu substrates support a surface‐mediated growth mechanism due to their extremely low carbon solubility (≈0.001 atomic% at 1000 °C).^[^ ^206^ ^]^ Graphene synthesis on Cu occurs via catalytic decomposition of CH_4_ at high temperatures, producing active carbon species that adsorb on the surface and nucleate into MG.^[^ ^207^ ^]^ This process is kinetically self‐limiting: Once a monolayer fully covers the Cu surface, further adsorption and growth are suppressed, thereby preventing the formation of additional layers. The quality and uniformity of the graphene are highly sensitive to processing parameters such as CH_4_ partial pressure, growth temperature, and substrate crystallinity. Specifically, lower precursor pressures and high‐temperature conditions favor larger grain sizes and reduced nucleation densities. Furthermore, the grain size of the Cu substrate significantly influences the graphene domain size, as larger Cu grains reduce the density of grain boundaries, which in turn enhances carrier mobility and other electronic transport properties.^[^ ^198^ ^]^ Beyond conventional Cu and Ni foils, functionalized or patterned substrates have been explored to produce engineered graphene architectures. For instance, Wang et al.^[^ ^197^ ^]^ introduced a novel direct‐growth strategy for synthesizing graphene nanomeshes (GNMs) on patterned Cu foils using nanosphere lithography (**Figure** **16**a). In their approach, polystyrene (PS) nanospheres were deposited onto the Cu surface to create a templated pattern prior to low‐pressure CVD. The resulting GNMs exhibited a thickness of ≈1.1 nm, as measured by atomic force microscopy (AFM), indicative of a monolayer structure (Figure 16b). Raman spectroscopy revealed an ID/IG ratio in the range of 0.6 to 0.9—lower than that observed in post‐etching GNMs—indicating fewer structural defects and smoother edges, thereby confirming the high quality of the directly grown GNM (Figure 16c).^[^ ^197^ ^]^

Compared with epitaxial growth of graphene, CVD remains the most widely adopted technique for graphene synthesis. For example, Yang et al.^[^ ^208^ ^]^ employed a two‐step CVD approach to selectively grow BLG with Bernal (AB) stacking. In this process, an MG film was first synthesized on a copper (Cu) substrate, followed by a second CVD growth cycle at 950 °C to deposit the second graphene layer, resulting in well‐ordered BLG with controlled stacking orientation. In contrast, Li et al.^[^ ^209^ ^]^ synthesized FLG via a CVD process using a CH_4_/H_2_/N_2_ gas mixture under varying temperatures from 960 to 1020 °C. Optimal growth conditions at 1020 °C produced high‐quality FLG with 4–10 layers and a low D/G ratio of 0.14, signifying minimal structural defects.

**Figure 17:**
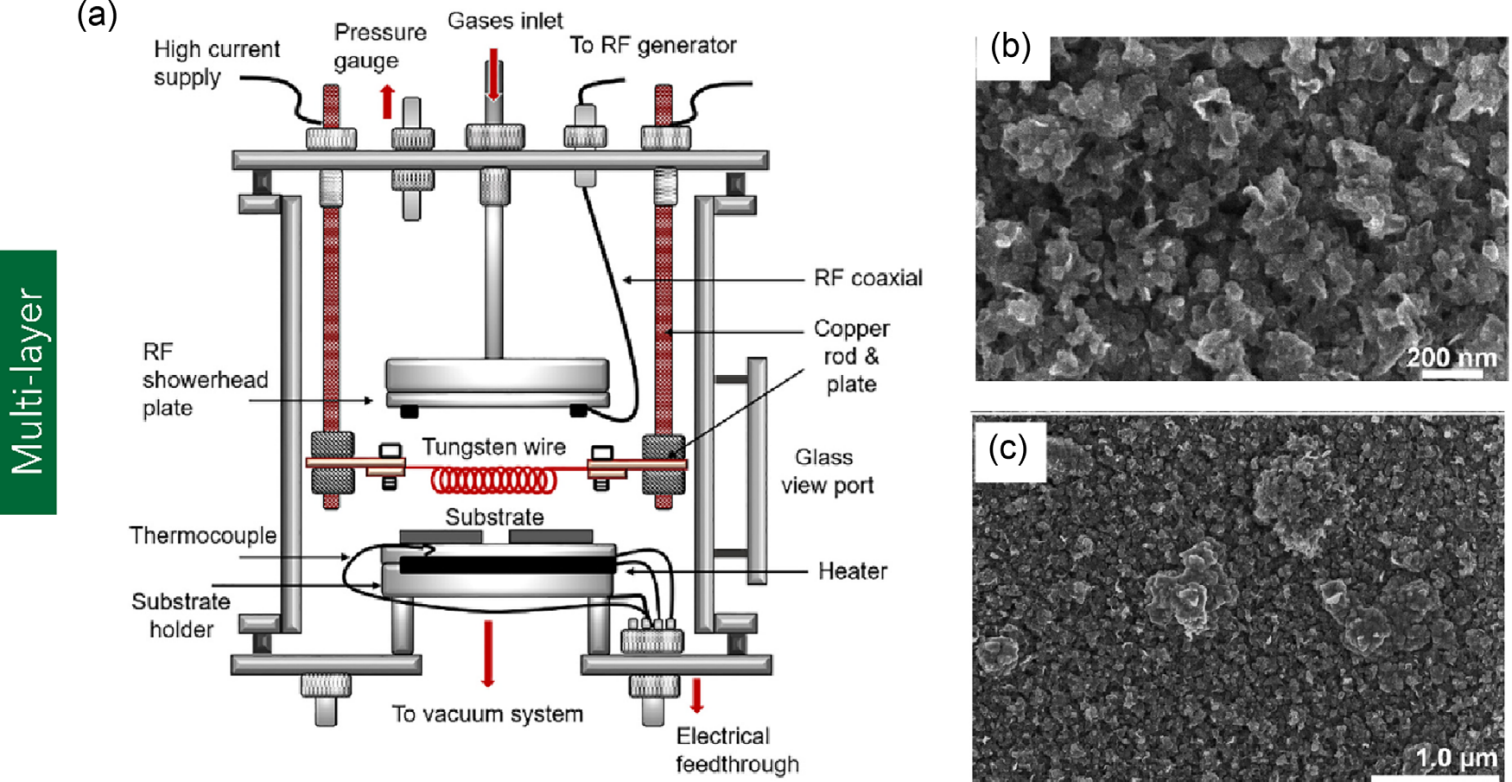
a) Schematic representation of the HWCVD system used for MLG synthesis. b,c) SEM images of MLG synthesized via HWCVD system. (b,c) are reproduced with permission.^[^ ^210^ ^]^ Copyright 2021, Elsevier B.V.

**Figure 18:**
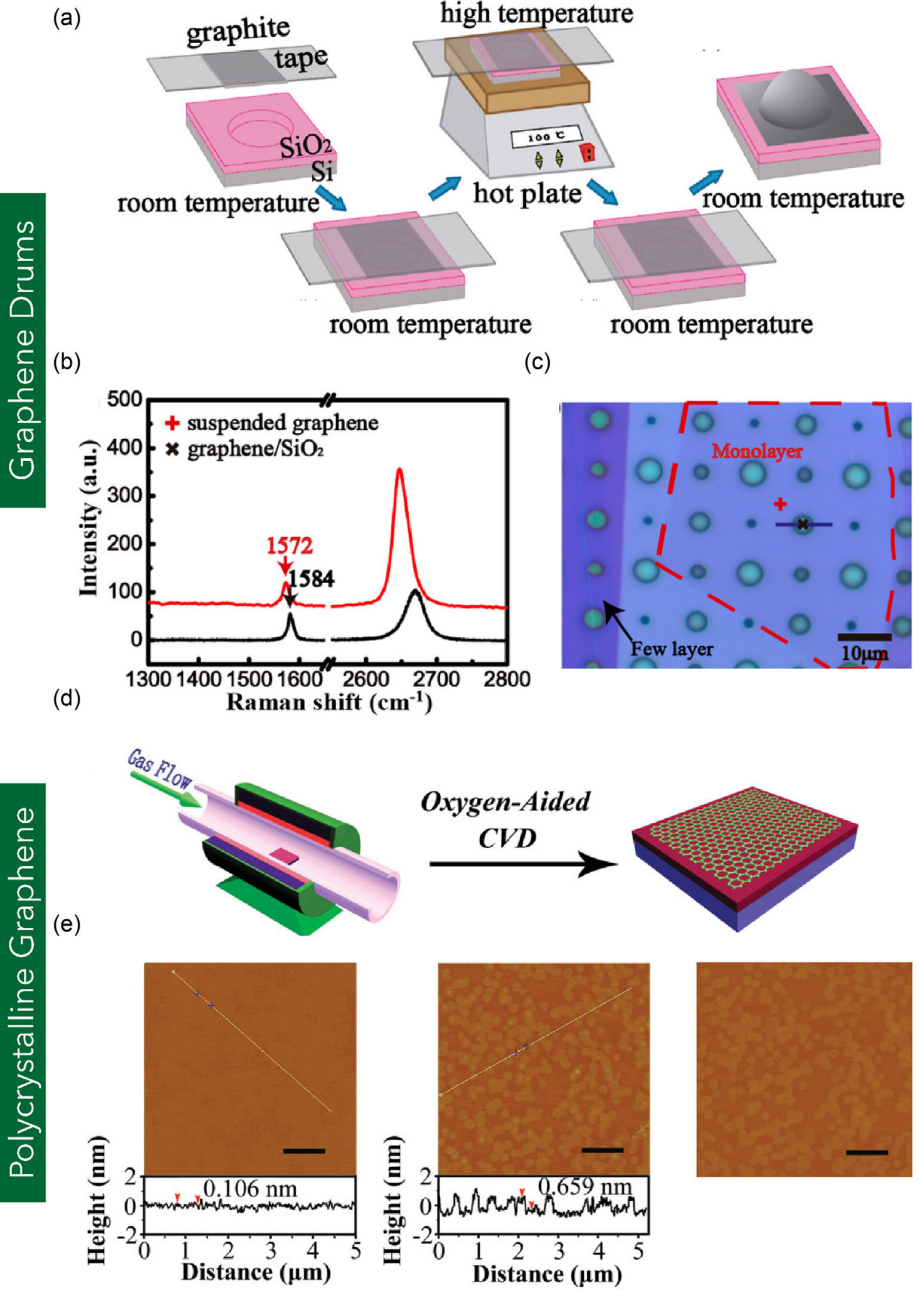
a) Schematic representation of the fabrication process for graphene drums on SiO_2_/Si(100) substrates. b,c) Optical microscopy images of MG and FLG, along with Raman spectroscopy comparison between MG and suspended graphene membranes on SiO_2_/Si(100) substrates. (a–c) are reproduced with permission.^[^ ^124^ ^]^ Copyright 2018, American Chemical Society. d) Illustration of the oxygen‐assisted CVD process used to synthesize PG on SiO_2_ substrates. e) AFM Z‐height images comparing the surface profile of the bare SiO_2_ substrate and the CVD‐grown PG. (d,e) are reproduced with permission.^[^ ^211^ ^]^ Copyright 2011, American Chemical Society.

In another study, Anuar et al.^[^ ^210^ ^]^ employed a hot‐wire CVD (HWCVD) approach to grow MLG on silicon and quartz substrates. By modulating the argon plasma treatment time during the deposition process, the group effectively controlled the thickness and quality of the resulting MLG films. The fabricated MLG exhibited optical transmittance in the range of 40%–70%, with corresponding sheet resistances of 718.3 Ω sq^−1^ for thinner films (10–15 layers) and 965.6 Ω sq^−1^ for thicker films (>15 layers), as shown in **Figure** **17**a–c. Sarafraz et al.^[^ ^126^ ^]^ employed CVD and a wet transfer process to fabricate graphene drums on Si/SiO_2_ substrates. Circular holes with diameters ranging from 60 to 1000 μm were etched into the substrate using reactive ion etching. MLG grown via Mo‐catalyzed CVD on a poly(methyl methacrylate) (PMMA) support was transferred onto the substrates, and the PMMA layer was removed using acetone. The resulting suspended drums exhibited non‐uniform but extremely low stress distribution (<0.0013%). The spatially averaged radial and azimuthal stress values were measured at 3.76 and 2.80 MPa, respectively, with a nominal stress of 7.05 MPa (**Figure** **18**a–c).^[^ ^126^ ^]^

CVD remains the primary technique for synthesizing PG. For example, Chen et al.^[^ ^211^ ^]^ employed an oxygen‐assisted CVD method to grow PG on SiO_2_/Si and quartz substrates at a reaction temperature of 1100 °C. The gas mixture used during growth consisted of methane (CH_4_, 14 sccm), hydrogen (H_2_, 50 sccm), and argon (Ar, 65 sccm). Prior to growth, the substrates were pre‐annealed at 800 °C to activate nucleation sites, which subsequently guided the formation of grain boundaries during graphene growth. The resulting PG exhibited a uniform thickness of ≈0.659 nm on the Si substrate, with clearly defined nucleation‐induced grain boundary patterns (Figure 18d,e). The CVD growth temperature plays a critical role in determining the crystalline grain size of the resulting PG. Paul et al.^[^ ^212^ ^]^ demonstrated the influence of temperature by utilizing ethanol vapor‐assisted CVD to grow PG on copper substrates. By varying the growth temperature between 900 and 1000 °C, they observed that the catalytic decomposition of ethanol modulated surface kinetics, thereby influencing grain boundary formation. The resulting graphene crystals exhibited grain sizes of ≈2 nm at 900 °C and 3 nm at 1000 °C, highlighting the temperature‐dependent nature of crystalline domain development in PG.

Recent advancements in CVD techniques have focused on precisely engineering to enable the synthesis of large‐area, single‐crystalline graphene nanostructures. One notable strategy involves vapor‐trapping configurations, establishing a quasi‐static reaction zone within a confined quartz enclosure. This modified setup alters the gas‐phase dynamics by restricting convective flow and promoting diffusion‐limited transport, thereby reducing the nucleation density on the substrate surface.^[^ ^213^ ^]^ Such an environment favors the anisotropic growth of graphene domains, resulting in distinctive flower‐like morphologies characterized by six‐lobed, single‐crystalline structures with bilayer graphene cores.^[^ ^214^ ^]^ SEM imaging reveals domain sizes reaching up to ≈100 μm. Raman spectroscopy further confirms the monolayer nature of the lobes, exhibiting characteristic I_2_D/I_G intensity ratios and a narrow full‐width at half‐maximum (FWHM) of ≈33 cm^−1^ for the 2D band, consistent with high‐quality MG.^[^ ^215^ ^]^ The growth kinetics under vapor‐trapped conditions are governed by diffusion‐limited aggregation mechanisms and anisotropic edge propagation, which are highly sensitive to CH_4_ concentration and the local carbon flux within the gas phase. These tailored growth regimes suppress random nucleation while enabling directional crystal expansion, ultimately improving the structural integrity and uniformity of the graphene. Such innovations significantly enhance the structural and electronic properties of the resulting graphene, making it particularly suitable for high‐performance electronic applications. For example, FETs fabricated using these high‐quality graphene domains have demonstrated carrier mobilities exceeding 4200 cm^2^ V^−1^ s^−1^ under optimized conditions,^[^ ^216^ ^]^ highlighting the promise of advanced CVD methods in scalable graphene electronics.

In addition to achieving controlled growth of wafer‐scale, single‐crystalline 2D films, future research directions for CVD‐fabricated graphene and other 2D materials include: 1) the direct synthesis of vertical and lateral van der Waals (vdW) heterostructures through sequential or co‐growth of stacked 2D layers (e.g., graphene/MoS_2_);^[^ ^217^, ^218^ ^]^ 2) the incorporation of dopants such as nitrogen (N), boron (B), and phosphorus (P) to modulate electrical, optical, and catalytic properties;^[^ ^219^ ^]^ and 3) integration with CMOS platforms by embedding 2D materials directly into established semiconductor fabrication workflows.

### 3.3 Oxidation and Reduction

**Figure 19:**
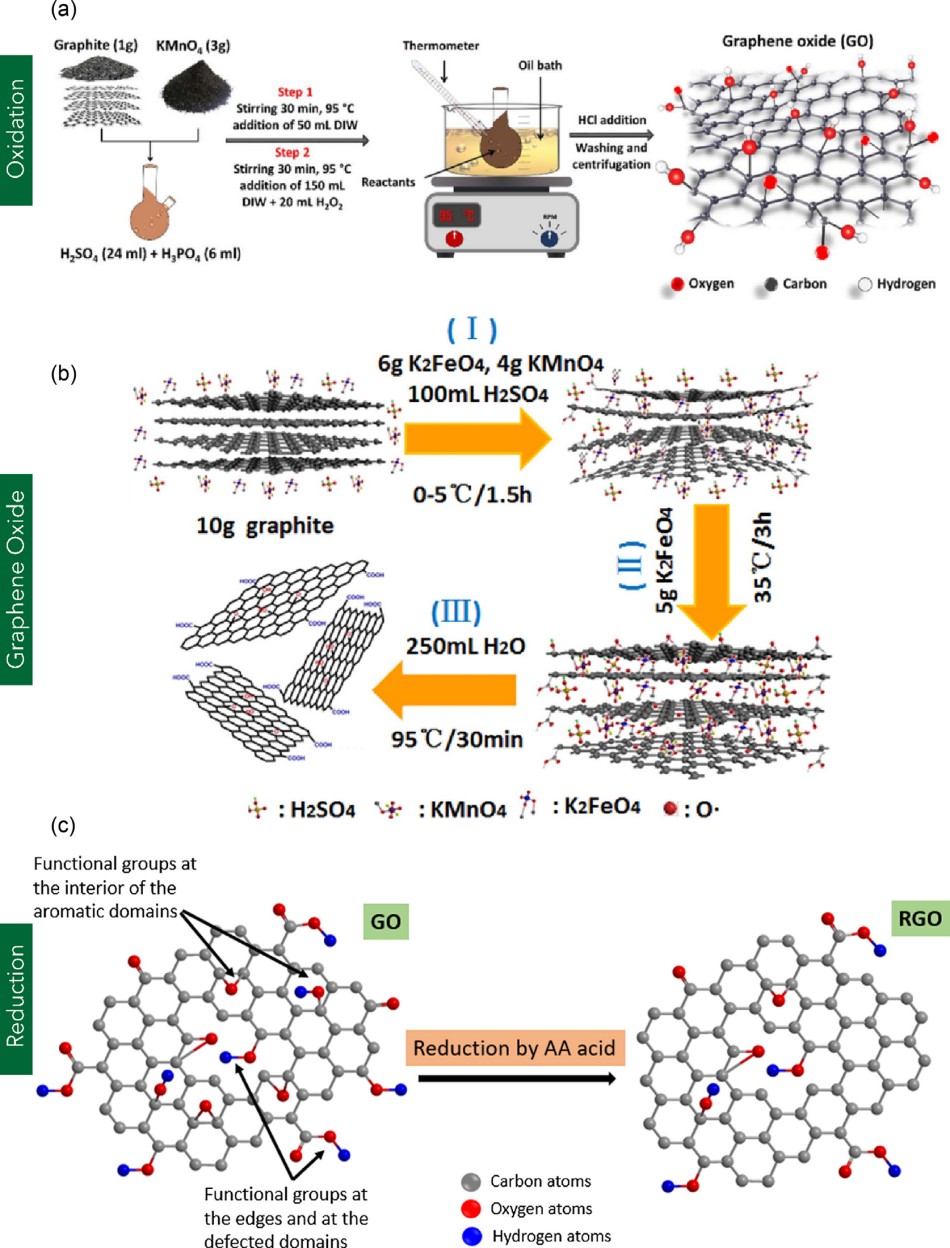
a) Schematic representation of graphite oxidation using the modified Hummers method. Reproduced under the terms of the CC‐BY license.^[^ ^401^ ^]^ Copyright 2021, the authors, published by MDPI. b) Schematic illustration of the GO synthesis process using the modified Hummers method. Reproduced under terms of the CC‐BY license.^[^ ^226^ ^]^ Copyright 2016, The Authors, published by Nature Portfolio. c) Chemical reduction of GO using C_6_H_8_O_6_. Reproduced with permission.^[^ ^232^ ^]^ Copyright 2018, Elsevier B.V.

Among the various top–down strategies for graphene synthesis, the chemical oxidation of graphite to GO, followed by its reduction to RGO, remains one of the most widely employed methods (Figure 14e,f). Four primary techniques have been established for GO preparation: the Brodie method,^[^ ^220^ ^]^ the Staudenmaier method,^[^ ^221^ ^]^ the Hofmann method,^[^ ^222^ ^]^ and the Hummers method.^[^ ^223^ ^]^ Among these, the modified Hummers method remains the most widely employed due to its scalability and high oxidation efficiency (Figure 14g). Figure 14h shows the scheme of LPE. **Figure** **19**a schematically depicts the GO synthesis process via a modified Hummers method. For example, Marcano et al.^[^ ^224^ ^]^ developed an improved Hummers method using a H_2_SO_4_/H_3_PO_4_ acid mixture as the oxidation medium, replacing the conventional use of NaNO_3_ and thereby eliminating the generation of toxic gases such as NO_2_ and N_2_O_4_. This approach also offers better temperature control and oxidation uniformity. The resulting GO exhibited an expanded interlayer spacing of ≈0.625 nm, compared with 0.335 nm for pristine graphite, indicating successful oxidation and intercalation. X‐ray photoelectron spectroscopy (XPS) analysis revealed that the synthesized intermediate graphene oxide contained ≈69% oxidized carbon and 31% graphitic carbon, reflecting significant disruption of the sp^2^ carbon lattice. Chen et al.^[^ ^225^ ^]^ also adopted an environmentally friendly variation of the improved Hummers method to fabricate GO with a C/O atomic ratio of ≈2.36, which closely matched the typical C/O ratio (≈2.23) of conventionally prepared GO. The resulting GO exhibited excellent aqueous dispersibility, confirmed by a zeta potential of –43.8 mV. Thermogravimetric analysis showed an initial weight loss below 100 °C due to the evaporation of adsorbed water, followed by a major decomposition event between 200 and 230 °C associated with the thermal breakdown of oxygen‐containing groups. In another study, Yu et al.^[^ ^226^ ^]^ synthesized GO using a refined version of the Hummers method. The resulting GO sheets exhibited thicknesses in the range of 1.5–2 nm, corresponding to ≈2–3 atomic layers. XRD analysis indicated an interlayer spacing of 0.833 nm. Thermal analysis revealed major mass loss around 200 °C due to the decomposition of labile oxygen functionalities, with additional weight loss occurring above 250 °C from residual oxygen groups (Figure 19b).

Chemical reduction is the commonly employed technique for converting GO into RGO. Among the various reducing agents explored, N_2_H_4_, sodium borohydride (NaBH_4_), and C_6_H_8_O_6_ are widely utilized due to their effectiveness and accessibility.^[^ ^227^ ^]^ N_2_H_4_, a strong nucleophilic and antioxidative reagent, is particularly efficient in reducing GO by removing oxygen‐containing functional groups. Experimental studies have shown that N_2_H_4_‐reduced GO can attain a high C/O atomic ratio of ≈10.3 and electrical conductivities up to 2420 S m^−1^, closely approaching that of pristine graphene (≈2500 S m^−1^).^[^ ^228^ ^]^ However, the reduction process often results in nitrogen doping, where nitrogen atoms incorporate into the graphene lattice as amine or pyrrolic groups. This unintended doping introduces structural defects, modifies the electronic band structure, shifts the Fermi level, and generates mid‐gap states—factors that may be beneficial or detrimental depending on the target application.^[^ ^175^ ^]^ NaBH_4_, a metal hydride‐based reducing agent, reduces GO primarily through hydride transfer reactions targeting —C=O and —OH groups. The tetrahedral borohydride ion (BH_4_^−^) acts as a nucleophile, donating hydride ions (H^−^) to electrophilic oxygen atoms, forming water and hydrogenated carbon.^[^ ^229^ ^]^ GO reduced by NaBH_4_ typically achieves a C/O ratio of ≈8.6 and an electrical conductivity of ≈45 S m^−1^.^[^ ^230^ ^]^ Unlike N_2_H_4_, NaBH_4_ does not introduce nitrogen dopants, yielding a more chemically pure RGO. However, FTIR often reveals residual boron‐oxygen species on the graphene surface. These species may act as charge‐scattering centers, potentially degrading carrier mobility.^[^ ^231^ ^]^ Owing to its moderate reduction strength and minimal functionalization, NaBH_4_ is well‐suited for thermal and electronic applications that require enhanced electrical properties with reduced structural disruption. C_6_H_8_O_6_, a naturally occurring antioxidant, has emerged as an environmentally benign reducing agent for GO. Its reduction mechanism is driven by nucleophilic electron donation from enediol groups (Figure 19c).^[^ ^232^ ^]^ The process proceeds via sequential reduction of epoxide and hydroxyl functionalities, forming water and restoring extended sp^2^‐conjugated domains. Raman spectroscopy of C_6_H_8_O_6_‐reduced GO reveals a high G to D band ratio, indicating significant lattice restoration with fewer defect sites.^[^ ^233^, ^234^ ^]^ The resulting RGO typically exhibits a C/O ratio of ≈6 and electrical conductivity values up to ≈800 S m^−1^.^[^ ^234^ ^]^ The mild reaction conditions (e.g., ≈95 °C in aqueous media) preserve the mechanical integrity of the graphene sheets, maintaining tensile strength and flexibility. Additionally, the presence of residual carboxylic acid groups contributes to excellent dispersion stability in polar solvents, as evidenced by ζ‐potential measurements, which indicate strong electrostatic repulsion.^[^ ^235^ ^]^

### 3.4 Miscellaneous Synthesis Techniques

Mechanical exfoliation, CVD, and oxidation–reduction remain the most widely used synthesis techniques. Abreast of these strategies, several unique miscellaneous synthesis techniques are also investigated across different graphene nanostructures. For example, BLG can be synthesized through various fabrication strategies, controlled folding, chemical intercalation, and the cutting–rotation–stacking method. Each technique offers distinct advantages in terms of structural control, scalability, and twist angle tunability. Wang et al.^[^ ^236^ ^]^ demonstrated a controlled folding technique to fabricate twisted BLG with tunable interlayer angles ranging from 0° to 30°. Initially, MG was synthesized on a Cu(111) substrate and then transferred to a Si/SiO_2_ substrate engineered with hydrophilic and hydrophobic surface regions. By folding the delaminated section over the fixed region, the authors achieved BLG structures with precise angular control and high uniformity. Zhu et al.^[^ ^237^ ^]^ developed a room temperature, lithium intercalation‐assisted chemical exfoliation method to produce BLG at scale. Biphenyl‐lithium (Bp‐Li) was intercalated into bulk graphite, facilitating a reduction in interlayer spacing. Upon dispersion in water, the intercalated structure exfoliated into high‐quality BLG, characterized by a low defect density (ID/IG = 0.14) and a high C/O atomic ratio (≈29.7). This method yielded BLG with a high efficiency of ≈78%. Chen et al.^[^ ^238^ ^]^ introduced a novel cutting–rotation–stacking technique to fabricate bilayer and trilayer graphene with adjustable twist angles. In this method, CVD‐grown MG was precisely cut using a femtosecond laser and then transferred onto a Si/SiO_2_ substrate, where it was manually stacked at desired rotational angles. This approach enables deterministic control over the twist angle, facilitating studies of angle‐dependent electronic and optical phenomena in layered graphene systems.

MLG can be synthesized through various techniques, including thermal annealing and layer exchange processes. For example, Zhao et al.^[^ ^239^ ^]^ reported the fabrication of MLG from coal using a hydrochloric acid‐assisted thermal annealing process at 200 °C. The resulting MLG exhibited an interlayer spacing of ≈0.33 nm, as confirmed by XRD analysis. Additionally, the product demonstrated a Brunauer–Emmett–Teller surface area of 23.7 m^2^ g^−1^ and a high carbon purity of 96.88%. Toko et al.^[^ ^98^ ^]^ developed a low‐temperature (350 °C) layer exchange technique by initially depositing amorphous carbon films on substrates, followed by thermal annealing. This method yielded MLG with an electrical conductivity of 2700 S cm^−1^ and a favorable IG/ID ratio in the Raman spectra (ranging from 0.9 to 21.3), indicating relatively low defect concentrations in the basal plane.

### 3.5 Other Graphene Nanostructure Synthesis

#### 3.5.1 Graphene Nanoribbons (GNRs) Synthesis

GNRs can be synthesized via top‐down approaches, such as patterning of MG, oxidative unzipping of CNTs, and lithographic techniques, or via bottom–up approaches, including on‐surface synthesis and CVD. One widely used method is oxidative unzipping, as demonstrated by Kosynkin et al.^[^ ^240^ ^]^ who treated sulfuric acid‐functionalized multiwalled carbon nanotubes (MWCNTs) with 500 wt% potassium permanganate (KMnO_4_) for 1 h at room temperature. This process yielded GNRs with widths exceeding 100 nm and lengths up to 4 μm. However, the extensive oxidative treatment led to heavily functionalized edges, resulting in low electrical conductivity (≈10^−6^ A at 0.5 V). Post‐treatment with N_2_H_4_ reduced the oxygen content from 42% to 16%, significantly enhancing the conductivity to 10–15 μA at 0.5 V. In another study, Wu et al.^[^ ^241^ ^]^ employed laser interference lithography to fabricate large‐scale GNR arrays on silicon substrates using a 1030 nm laser with power ranging from 0.4 to 2 W. By finely tuning the laser parameters, the authors achieved GNRs with uniform widths of 300–500 nm and lengths extending up to 42 μm. The produced GNRs exhibited a D/G peak intensity ratio of 0.51 in Raman spectroscopy, indicative of low structural disorder and high crystallinity, both essential for maintaining electrical conductivity. Alternatively, Ruffieux et al.^[^ ^242^ ^]^ demonstrated the bottom‐up synthesis of atomically precise zigzag‐edged GNRs through surface‐assisted polymerization followed by cyclodehydrogenation. U‐shaped molecular precursors were thermally deposited onto an Au(111) substrate and annealed at 625 K to induce polymerization and planarization via cyclodehydrogenation. The resulting GNRs exhibited well‐defined zigzag edges and demonstrated spin‐polarized edge states with energy splittings of Δ_0_ = 1.5 eV and Δ_1_ = 1.9 eV, rendering them promising candidates for spintronic applications.

In addition to these approaches, bottom‐up synthesis of GNRs using on‐surface and CVD methods has shown significant progress. For instance, Cai et al.^[^ ^243^ ^]^ described a metal surface‐assisted method for creating atomically precise GNRs. The process involved two key thermal activation steps on a metal surface, such as Au(111) or Ag(111). First, di‐halogenated molecular precursor monomers (10,10′‐dibromo‐9,9′‐bianthryl) were sublimed onto the heated surface (≈200–250 °C), which created surface‐stabilized radical species. In a second annealing step at a higher temperature (≈400–440 °C), an intramolecular cyclodehydrogenation (fusion) reaction occurred, converting the nonplanar polymer into a fully aromatic, flat graphene nanoribbon. The width, edge structure, and complex shapes were defined with atomic precision by the chemical structure of the original precursor monomers. Räder et al.^[^ ^244^ ^]^ leveraged CVD at ambient pressure conditions to fabricate GNRs. 6,11‐Dibromo‐1,2,3,4‐tetraphenyltriphenylene (molecular precursor) was sublimed on to Au/mica substrate at 200–250 °C under a gas flow of Ar and H_2_. On the heated gold surface, the monomers underwent thermal dehalogenation and generated reactive biradical species that diffused and underwent radical addition polymerization to form linear, chevron‐type polyphenylene precursors. After this, post‐annealing at 400–450 °C was carried out to induce surface‐assisted intramolecular cyclodehydrogenation, converting the polymeric chains into fully aromatic, structurally defined GNRs.

#### 3.5.2 Graphyne Synthesis

**Figure 20:**
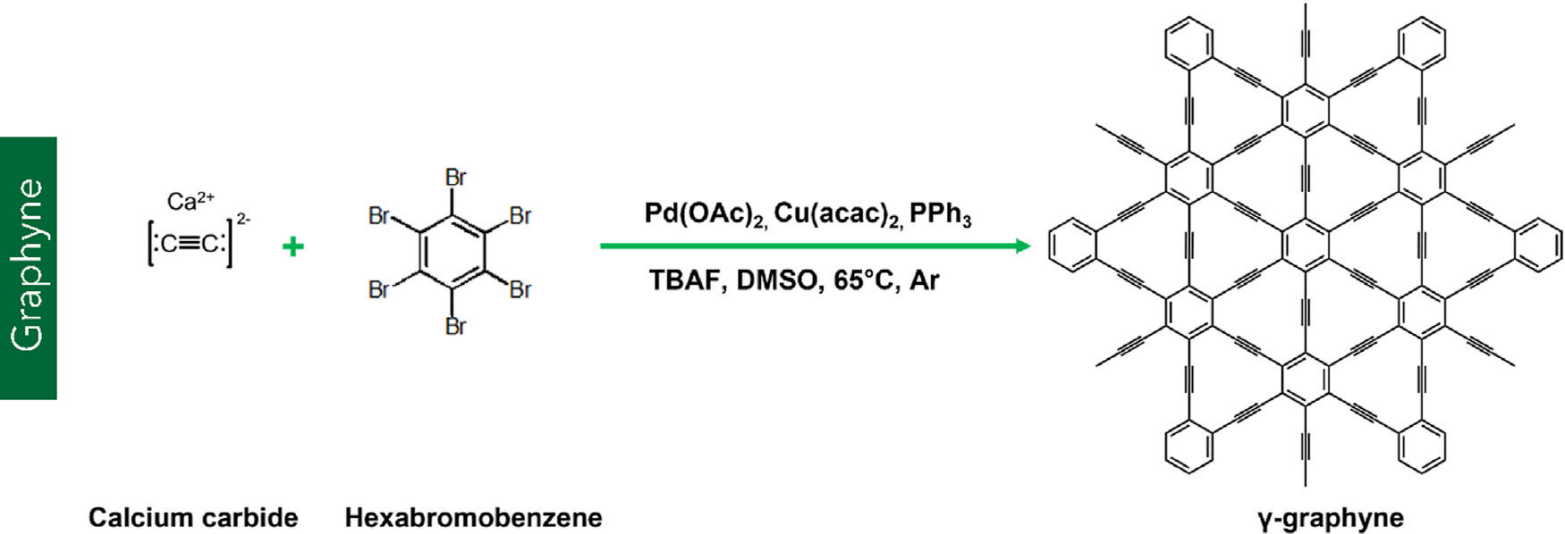
Reaction scheme depicting the synthesis of γ‐graphyne from hexabromobenzene and calcium carbide. It is reproduced with permission.^[^ ^245^ ^]^ Copyright 2022, Elsevier Ltd.

Graphyne can be synthesized using a variety of bottom‐up chemical and mechanochemical approaches, including cross‐coupling reactions and ball milling techniques. One of the most promising methods for fabricating γ‐graphyne involves the Sonogashira cross‐coupling reaction, as demonstrated by Barua et al.^[^ ^245^ ^]^ In this approach, calcium carbide (CaC_2_) and hexabromobenzene (HBrB) were utilized as precursors under mild reaction conditions to synthesize γ‐graphyne in bulk (**Figure** **20**). The resulting material exhibited high purity, a semiconducting bandgap of 2.3 eV, and a large BET surface area of 473 m^2^ g^−1^. Additionally, the synthesized γ‐graphyne showed excellent charge transport characteristics, confirming its suitability for electronic and energy storage applications. In a complementary study, Li et al.^[^ ^246^ ^]^ introduced a high‐yield, catalyst‐free route to synthesize γ‐graphyne via a mechanochemical approach. This method employed ball milling of CaC_2_ and HBrB, where mechanical force served as the sole energy input to drive the cross‐coupling reaction, eliminating the need for elevated temperatures or catalytic agents. The resulting γ‐graphyne displayed p‐type semiconducting behavior with a bandgap of 2.58 eV, consistent with the values reported by Barua et al.^[^ ^245^ ^]^ The material also exhibited a conduction band minimum at −0.05 V, further supporting its potential use in electronic and optoelectronic devices.^[^ ^246^ ^]^

### 3.6 Polymer Composite Fabrication

#### 3.6.1 Coating

**Figure 21:**
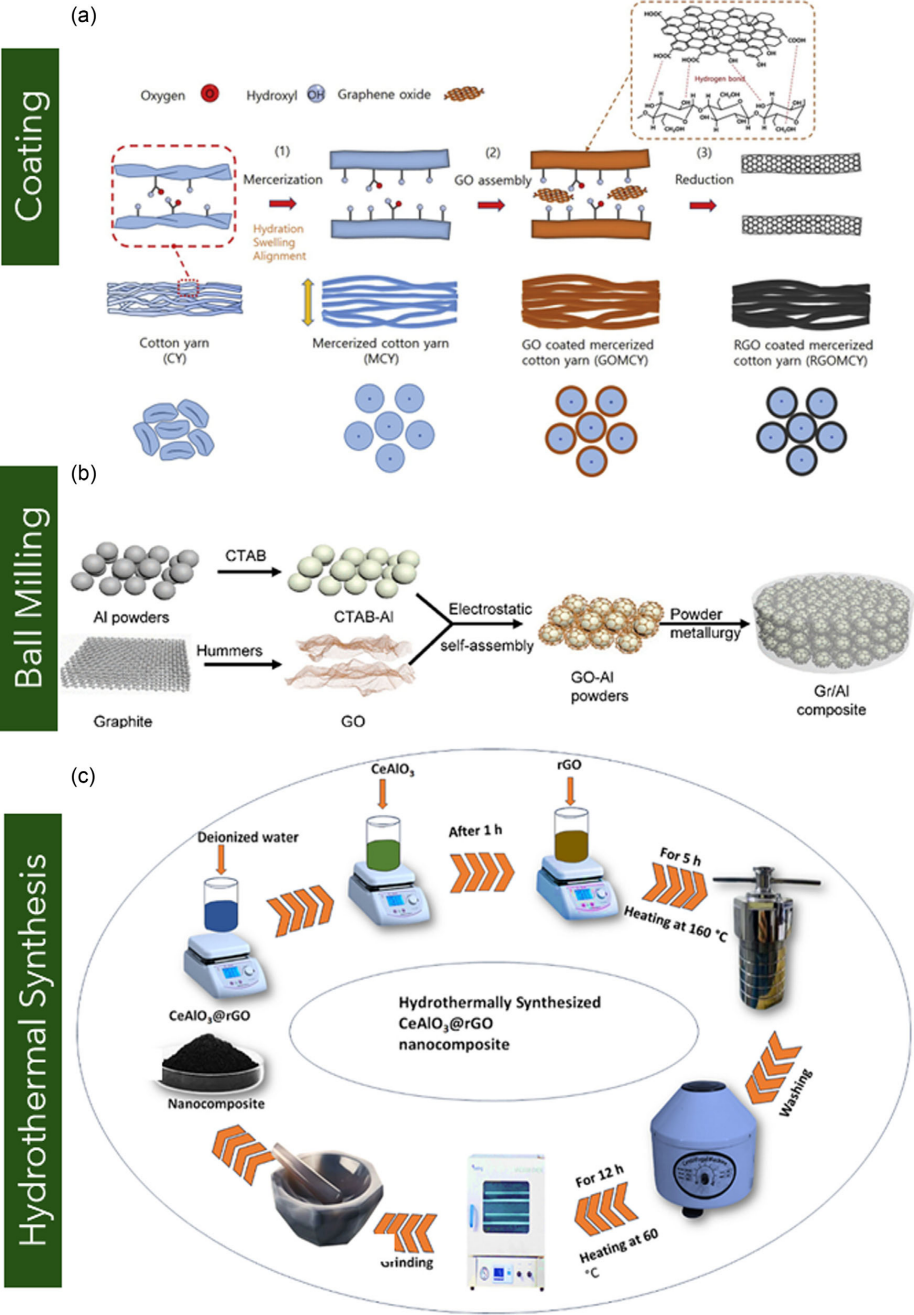
a) Scheme depicting the dip‐coating process for fabricating RGO‐coated cotton yarn composites. Reproduced with permission.^[^ ^248^ ^]^ Copyright 2019, Elsevier Ltd. b) Scheme depicting the fabrication process of Gr/Al composites. Reproduced with permission.^[^ ^262^ ^]^ Copyright 2024, Elsevier B.V. c) Scheme depicting the hydrothermal synthesis of CeAlO_3_@RGO nanocomposites. Reproduced with permission.^[^ ^268^ ^]^ Copyright 2024, Elsevier Ltd.

Coating methods, in particular, have demonstrated significant potential by enabling the uniform deposition of graphene or its derivatives (e.g., GO or RGO) onto polymeric or inorganic substrates to form functional composite structures. Several coating techniques have garnered considerable attention, including dip coating,^[^ ^247^, ^248^ ^]^ spin coating,^[^ ^249^ ^]^ spray coating,^[^ ^250^ ^]^ and electrophoretic deposition (EPD).^[^ ^251^ ^]^ In dip coating, substrates are immersed in a GO or RGO suspension—typically with concentrations ranging from 1 to 10 mg mL^−1^—followed by a controlled drying process and, if needed, a subsequent chemical or thermal reduction step (**Figure** **21**a).^[^ ^252^, ^253^ ^]^ This technique enables the formation of conductive coatings with electrical conductivities on the order of ≈10^3^ S m^−1^, suitable for flexible electronic and sensor applications.^[^ ^254^ ^]^ Spin coating provides precise control over film thickness by rotating the substrate at speeds typically between 1000 and 6000 rpm. This method yields thin films with thicknesses ranging from ≈50 nm to several micrometers, making it ideal for optoelectronic devices requiring uniform thin layers.^[^ ^249^ ^]^ In contrast, spray coating employs atomized GO suspensions delivered at pressures of ≈0.34 bar, allowing for scalable, large‐area deposition with excellent uniformity—an advantage for industrial‐scale manufacturing.^[^ ^255^ ^]^ EPD offers another powerful approach, in which a DC voltage (typically 20–100 V) is applied across electrodes submerged in a colloidal GO suspension. The charged GO particles migrate and deposit onto the substrate surface, enabling uniform coating even on complex or non‐planar geometries. The final film thickness is governed by the deposition time and applied voltage.^[^ ^251^, ^256^ ^]^ These coating techniques have been effectively integrated into graphene–polymer composite systems, demonstrating enhancements in mechanical performance (with tensile strength improvements of ≈20%–50%) and thermal conductivity increases up to ≈2 W m^−1^ K^−1^, compared with the baseline polymer or graphene material alone. Despite these advantages, challenges remain in achieving strong interfacial adhesion, uniform dispersion, and reproducibility—particularly in large‐scale applications. These limitations can be addressed through optimization of surface functionalization, dispersion stability, and deposition parameters.^[^ ^257^ ^]^

#### 3.6.2 Ball Milling

Ball milling is a widely adopted mechanical approach for fabricating graphene‐based composites, offering an efficient means to disperse graphene within metallic,^[^ ^258^ ^]^ polymeric,^[^ ^259^, ^260^ ^]^ or ceramic^[^ ^260^ ^]^ matrices while promoting strong interfacial interactions. In high‐energy ball milling, graphite or graphene precursors are subjected to mechanical shear and impact forces in the presence of a matrix material at rotation speeds typically ranging from 200 to 800 rpm, with milling durations spanning from 2 to 24 h.^[^ ^261^ ^]^ This process not only exfoliates graphite into FLG (3–10 layers) but also facilitates the uniform incorporation of graphene into the host matrix. For instance, Yang et al.^[^ ^262^ ^]^ synthesized graphene/aluminum (Gr/Al) composites via ball milling followed by a sintering process (Figure 21b). The resulting composite exhibited optimal mechanical performance at a graphene loading of 0.3 wt%, achieving a maximum tensile strength of 110 MPa and enhanced wear resistance. However, a further increase in graphene content to 0.5 wt% led to a decline in both tensile strength and tribological performance, likely due to graphene agglomeration and interface weakening.^[^ ^262^ ^]^ In another study, Li et al.^[^ ^263^ ^]^ utilized a combination of ball milling and spark plasma sintering (SPS) at 1600 °C to fabricate graphene–ceramic composites from graphene, boron, and silicon precursors. The composites exhibited excellent mechanical properties, including a flexural strength of 561 MPa, compressive strength of 2.17 GPa, and fracture toughness (the material's ability to resist the propagation of a pre‐existing crack) of 7.5 MPa $\sqrt{m}$, making them suitable for high‐performance structural applications. While ball milling offers advantages such as cost‐effectiveness, scalability, and versatility in composite design, prolonged milling durations can lead to structural degradation of graphene. Specifically, excessive milling may introduce defects, reduce lateral flake size, and significantly diminish electrical conductivity—dropping from values on the order of 10^4^ to 10^3^ S m^−1^.^[^ ^264^ ^]^ Therefore, careful optimization of milling parameters, including speed, time, ball‐to‐powder ratio, and atmosphere, is critical to preserving graphene's intrinsic properties and developing high‐performance composites with tailored mechanical, electrical, and thermal functionalities.

#### 3.6.3 Hydrothermal Synthesis

Hydrothermal synthesis is a solution‐based chemical method employed for the fabrication of graphene‐based composites through controlled reactions conducted at elevated temperatures (typically 100–300 °C) and pressures (up to ≈10 MPa) within a sealed autoclave environment.^[^ ^265^ ^]^ This technique enables the simultaneous reduction of GO and incorporating functional components—such as metal oxides, organic molecules, or polymers—into a composite matrix. Typically, GO suspensions with concentrations ranging from 2 to 5 mg mL^−1^ are mixed with selected precursors, facilitating in situ chemical reactions under hydrothermal conditions that yield well‐dispersed and structurally integrated nanocomposites.^[^ ^266^ ^]^ For example, Huang et al.^[^ ^267^ ^]^ synthesized graphene–TiO_2_ (G@T) composites via hydrothermal treatment at 180 °C for 12 h. The resulting G@T material demonstrated improved mechanical properties, including an increased softening point (48.5 °C) and enhanced rutting resistance, while retaining high photocatalytic activity, as evidenced by a 26% nitrogen oxide (NO) degradation rate under UV exposure after 30 min. In another study, Zahra et al.^[^ ^268^ ^]^ developed a CeAlO_3_@RGO nanocomposite using a hydrothermal route (Figure 21c). The composite exhibited exceptional electrochemical performance, including a high specific capacitance of 1442 F g^−1^, energy density of 57 Wh kg^−1^, and power density of 268 W kg^−1^, along with excellent cycling stability maintained over 5000 charge–discharge cycles, underscoring its suitability for high‐performance supercapacitor applications. Hydrothermal synthesis offers significant advantages, including the ability to tailor nanostructures and hierarchical morphologies by adjusting parameters such as precursor concentration, solution pH, reaction time, and temperature. This flexibility makes the technique particularly valuable for applications in energy storage, photocatalysis, and sensor technologies.^[^ ^265^ ^]^ However, the process can be time‐intensive and requires precise control over synthesis conditions to avoid agglomeration or phase separation within the composite matrix, especially when integrating graphene with polymeric systems. Figure 14i shows the scheme for coating, ball milling, and hydrothermal synthesis process. In addition, several simulation studies are also reported (Figure 14j).

#### 3.6.4 Other Techniques

Abreast of abovementioned strategies, graphene nanocomposites can be fabricated through solution mixing, in situ polymerization, and l‐b‐l assembly techniques.^[^ ^269^ ^]^ In the solution mixing method, graphene or its derivatives are physically blended with polymer solutions followed by casting and drying. For example, Dangi et al.^[^ ^270^ ^]^ fabricated PVA‐based RGO nanocomposites with varying RGO loadings (1, 2, and 3 wt%). The resulting composites exhibited tunable bandgap values of 2.67, 2.49, and 1.23 eV, respectively, demonstrating the ability to engineer the electronic properties of the composite by adjusting the graphene concentration. In situ polymerization is another widely adopted method, in which graphene is introduced directly into the monomer matrix before or during the polymerization process. This technique enhances interfacial interaction and ensures homogenous dispersion. For instance, Hong et al.^[^ ^271^ ^]^ developed a polydimethylsiloxane (PDMS)/graphene nanocomposite via in‐situ polymerization followed by shear exfoliation. The resulting composite functioned as a capacitive pressure sensor, exhibiting a pressure sensitivity of 0.0273 kPa^−1^—≈45 times higher than that of pristine PDMS, indicating a significant enhancement in sensing performance. To achieve precise control over the thickness, morphology, and architecture of multilayered structures, l‐b‐l assembly technique is particularly advantageous. Sha'rani et al.^[^ ^272^ ^]^ utilized l‐b‐l assembly to fabricate a graphene‐based nanocomposite membrane for vanadium redox flow batteries. The membrane was prepared by alternately depositing positively charged polyethylenimine and negatively charged GO layers. This hierarchical structure resulted in a 90% reduction in vanadium ion permeability and a high selectivity of 1.92 × 10^5^ S min cm^−1^, highlighting its suitability for energy storage applications.

Future research directions for graphene‐based polymer composites involve: 1) achieving uniform dispersion and controlled alignment of graphene nanosheets to harness their intrinsic anisotropic electrical, thermal, and mechanical properties; 2) developing functional gradient and multilayer architectures through advanced assembly techniques such as l‐b‐l deposition and sequential casting; and 3) enhancing multifunctionality by integrating graphene with other 2D materials to create hybrid composites with synergistic properties.

In summary, this section provides an overview of key synthesis strategies for preparing graphene nanostructures and graphene‐integrated composites, with an emphasis on their relevance to mechanical characterization. Specifically, CVD, epitaxial growth, and mechanical exfoliation techniques are discussed to fabricate pristine graphene nanostructures. Additionally, methods such as oxidative exfoliation and reduction, coating, ball milling, and hydrothermal synthesis are outlined to prepare graphene‐based composite systems. The subsequent section will focus on the mechanical characterization techniques applied to these synthesized graphene nanostructures and composites, highlighting their structure–property relationships and performance metrics.

### 3.7 Substrate Effects on Graphene Synthesis

The choice of substrate plays a decisive role in the synthesis, growth kinetics, and quality of graphene. Substrates not only serve as physical support but also actively influence nucleation density, domain orientation, defect formation, and layer number through their crystallographic structure, surface chemistry, and catalytic activity. Broadly, substrate effects can be classified into catalytic and non‐catalytic contributions, with underlying mechanisms tied to surface energy, lattice matching, and carbon solubility.^[^ ^273^ ^]^

On catalytic metal substrates, such as Ni, Co, and Fe, carbon atoms dissolve into the bulk at high temperatures and subsequently segregate onto the surface during cooling, yielding MLG.^[^ ^274^ ^]^ In contrast, metals with low carbon solubility—such as Cu, Au, and Ag—support surface‐mediated growth where carbon atoms adsorb, diffuse, and crystallize directly on the surface allowing controlled monolayer formation.^[^ ^275^ ^]^ For instance, Cu has become the most widely adopted substrate in CVD because its self‐limiting growth mechanism facilitates large‐area monolayer graphene with relatively low defect densities.^[^ ^276^ ^]^ The underlying mechanism is governed by the balance between carbon feedstock decomposition, surface diffusion, and nucleation at step edges or grain boundaries.

Substrate crystallography also determines graphene orientation and domain alignment. Single‐crystal Cu(111) substrates promote epitaxial alignment due to lattice compatibility, leading to fewer grain boundaries compared to polycrystalline Cu foils.^[^ ^276^ ^]^ Similarly, sapphire and h‐BN substrates provide atomically smooth, chemically inert surfaces that enable van der Waals epitaxy.^[^ ^277^ ^]^ In these cases, weak substrate interactions minimize defect nucleation while preserving graphene's intrinsic electronic properties. For example, h‐BN has been shown to support nearly strain‐free, high‐mobility graphene layers due to its close lattice match and dielectric compatibility.^[^ ^278^ ^]^

Beyond crystallographic effects, surface chemistry and morphology strongly affect nucleation kinetics. Rough or oxidized surfaces of substrates increase the density of active sites, thereby raising nucleation density but has a trade‐off for smaller domain sizes and increased grain boundary density.^[^ ^279^ ^]^ Pretreatments such as electropolishing of Cu foils or using single‐crystal substrates are therefore common strategies to improve graphene quality.^[^ ^280^ ^]^ In addition, insulating substrates like SiO_2_/Si are often employed as post‐growth supports rather than direct growth platforms. However, direct growth attempts on dielectric substrates using plasma‐enhanced CVD or metal‐catalyst transfer methods are gaining interest for device integration without the need for transfer processes.^[^ ^281^ ^]^

In short, substrates are not passive supports but active determinants of graphene synthesis pathways. Their catalytic activity, crystallography, and surface chemistry govern nucleation and growth dynamics, ultimately dictating the structural and electronic quality of the resulting graphene. Careful tailoring of substrate properties—whether through material choice, surface modification, or crystallographic engineering—remains central to advancing scalable, high‐quality graphene synthesis. **Table** **1** summarizes the key synthesis strategies for graphene nanostructure fabrication.

**Table 1:** Summary of key synthesis strategies to fabricate graphene nanostructures.

Synthesis process	Strategies to optimize graphene properties	Key tradeoffs	Benefits	Limitations
Mechanical exfoliation and cleaving	(1) Using HOPG with weak interlayer forces (2) Optimizing adhesive tape or substrate adhesion (3) Employing ultra‐smooth substrates (e.g., SiO_2_/Si) for visibility	Achieves the highest electronic quality but with extremely low, unpredictable yield and no scalability	(1) High‐quality graphene (2) Simple, low‐cost process	(1) Not scalable for mass production. (2) Tiny, random flake sizes.
Chemical vapor deposition	(1) Using low‐solubility catalysts like Cu for MG (2) Precise control of temperature, pressure, gas flow rates (low CH_4_:H_2_ ratio) (3) Using Cu–Ni alloys to tune carbon solubility and control layer number	Excellent scalability to wafer and roll‐to‐roll sizes, but process is complex and requires a damaging transfer step to insulating substrates	(1) Excellent scalability for large‐area, continuous films (2) Good control over layer number and domain size	(1) Requires high temperatures (2) Graphene has to be transferred
Epitaxial growth	(1) Precise control of Si sublimation temperature and rate (2) Using specific SiC polytypes (e.g., 4H‐SiC or 6H‐SiC)	Produces high‐quality, wafer‐scale graphene on a semi‐insulating substrate but is extremely expensive and the graphene is not freestanding	(1) Graphene is directly grown on a semi‐insulating SiC wafer, no transfer needed. (2) Wafer‐scale process compatible with microelectronics fabrication	(1) High cost of SiC substrates (2) Very high processing temperatures (>1400 °C)
Oxidation and reduction	(1) Controlling oxidation degree to minimize hole formation in the basal plane (2) Using chemical (hydrazine, HI), thermal, or electrochemical methods to reduce oxygen content and restore conductivity	Highly scalable and low‐cost, but the resulting material is highly defective and not pristine graphene	(1) Solution‐processable: enables printing, coating, and composite formation	(1) Reduction never fully restores the pristine graphene structure (rGO) (2) Low electrical conductivity compared with pristine graphene
Coating	(1) Using spray coating, spin coating, blade coating, or inkjet printing tailored to the application (2) Annealing process to remove solvents/binders and improve conductivity	Enables cheap, versatile deposition on any surface, but typically results in poor electrical performance and non‐uniform, multilayer films	(1) Can be applied to almost any surface or substrate (2) Ultra‐low‐cost and ambient processing	(1) No control over film uniformity or orientation. (2) Typically results in thick, multilayer films
Hydrothermal synthesis	(1) Controlling temperature and pressure to regulate reduction kinetics (2) Adding stabilizers to prevent restacking of graphene sheets	Uses water as a solvent (green process) but yields the same low‐quality, defective material as standard chemical reduction	(1) Scalable and low‐energy process compared to CVD/epitaxy (2) 3D graphene architectures like hydrogels and aerogels could be created	(1) Produced graphene has high defect density and poor electrical properties (2) Difficult to control the morphology of the final product

## 4 Mechanical Characterization Techniques for Graphene Nanostructures

**Figure 22:**
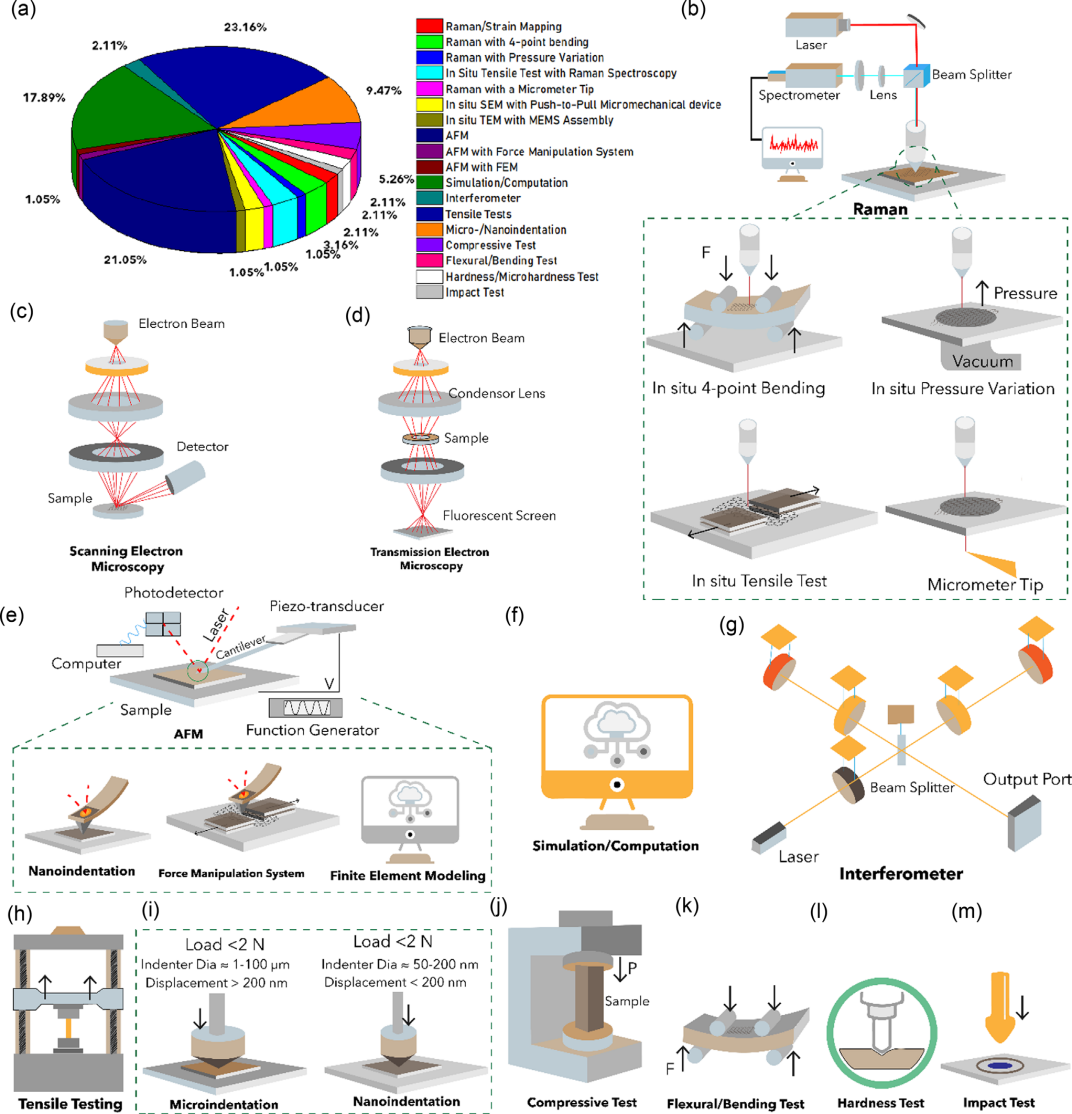
Mechanical characterization techniques for graphene nanostructures. a) Pie chart illustrating the distribution of employed mechanical characterization techniques. b) Schematic representation of Raman spectroscopy‐based mechanical testing, including in situ 4‐point bending, pressure variation, uniaxial tensile testing, and micrometer‐scale tip attachment, as shown in the inset. c) Scheme of SEM. d) Scheme of transmission electron microscope. e) AFM with nanoindentation, force manipulation system and FEA modeling attachment. f) Simulation/computation. g) Interferometer. h) Principle of tensile testing. i) Micro‐/nanoindentation test. j) Compressive testing. k) Flexural/bending test. l) Hardness testing. m) Impac*t* testing.

A variety of techniques have been employed to characterize the mechanical properties of graphene nanostructures, as illustrated in **Figure** **22**. Figure 22a depicts the distribution of employed mechanical characterization techniques. Among these, Raman spectroscopy, AFM, and in situ methods are particularly favored for nanoscale assessments due to their high spatial resolution and sensitivity. In contrast, conventional mechanical tests such as tensile, compression, and hardness testing are more commonly applied to bulk‐scale graphene‐based nanocomposites. Collectively, these methods enable accurate evaluation of key mechanical parameters across different structural hierarchies of graphene materials.

### 4.1 Raman Spectroscopy

Due to the atomic‐scale thickness of graphene and its derivatives, applying controlled mechanical strain to free‐standing nanosheets remains a considerable experimental challenge. A widely adopted approach involves transferring graphene onto flexible substrates, where mechanical deformation—through bending or stretching—induces tensile or compressive strain within the graphene layer. The applied strain could be monitored using Raman spectroscopy, which detects shifts in vibrational frequencies of characteristic phonon modes (Figure 22b). Specifically, tensile strain results in phonon softening, observed as a redshift in Raman peaks, whereas compressive strain leads to phonon hardening, corresponding to a blueshift.^[^ ^282^, ^283^ ^]^ Specifically, strain‐induced redshift and blueshift in graphene are observed in the G and 2D Raman bands, with the magnitude of the shift being proportional to the applied strain. For instance, a uniaxial tensile strain of ≈1% typically results in a downshift of ≈15 cm^−1^ in the G band and ≈30 cm^−1^ in the 2D band.^[^ ^284^ ^]^ These spectral shifts arise from alterations in bond lengths and bond angles within the graphene lattice under mechanical deformation, thereby allowing for the quantitative evaluation of strain and stress distributions within the material.^[^ ^285^ ^]^ The strain can be quantitatively described using the Grüneisen parameter,^[^ ^283^ ^]^ which relates the fractional change in vibrational frequency to the volume change of the crystal lattice. The stress transfer efficiency between the substrate and the graphene layer could be measured by a linear elastic model developed by Gong et al.^[^ ^286^ ^]^ as described in Equation (8).

(8) $$\varepsilon_{x}\left(x\right)=\varepsilon_{m}\left[1-\frac{\cosh\left(\beta x\right)}{\cosh\left(\frac{\beta L}{2}\right)}\right]$$

where $\varepsilon_{x}$ is the strain distribution in the graphene layer, $\varepsilon_{m}$ is the applied strain to the substrate, *L* is the length of 2D materials in the *x* direction at the center, and β is the shear‐lag parameter characterizing the critical length scale for effective load transfer through shear.

The validity of using Raman shifts as a quantitative probe of strain relies critically on the efficiency of strain transfer from the substrate to graphene. Imperfect adhesion, interfacial slippage, or wrinkle formation can lead to partial relaxation, causing the apparent Raman shift rates to underestimate the intrinsic Grüneisen parameters of graphene.^[^ ^283^ ^]^ For example, graphene on polymer substrates often exhibits lower shift rates compared to suspended membranes because the shear‐lag mechanism mediates load transfer. In this framework, the shear‐lag parameter *β* (Equation 8) assumes uniform adhesion and linear elastic deformation, which may not hold in practice due to interfacial roughness or viscoelasticity of the polymer. Consequently, extracted strain values should be interpreted as “effective strain” rather than absolute, unless validated by complementary techniques such as AFM indentation or direct displacement tracking.^[^ ^287^ ^]^

For macroscale considerations, Raman probes strain at micron or even submicron spots, which may not represent the average strain across an entire flake. Inhomogeneities—such as wrinkles, cracks, or local slippage—can broaden the peaks (as observed by Bissett et al.)^[^ ^288^ ^]^ and mask intrinsic responses. Therefore, extracting elastic constants from Raman requires either spatial mapping to ensure homogeneity or modeling frameworks that bridge local vibrational shifts with continuum‐scale mechanics.

A key indicator of strain in graphene is the splitting of the G band into G^+^ and G^−^ components under uniaxial strain, which arises because the doubly‐degenerate E_2_g phonon mode is symmetry‐broken. In contrast, the 2D band originates from a double‐resonant process involving intervalley scattering.^[^ ^289^ ^]^ Its shift is dominated by changes in electronic band structure rather than symmetry breaking, which explains the absence of clear splitting even under significant strain.^[^ ^290^ ^]^ Disentangling strain from other effects such as doping is also critical, since electron/hole doping can stiffen the G mode (blue shift) while affecting the 2D band differently. Polarization‐resolved Raman measurements and the correlation between G and 2D shifts are commonly used to separate these effects.

**Figure 23:**
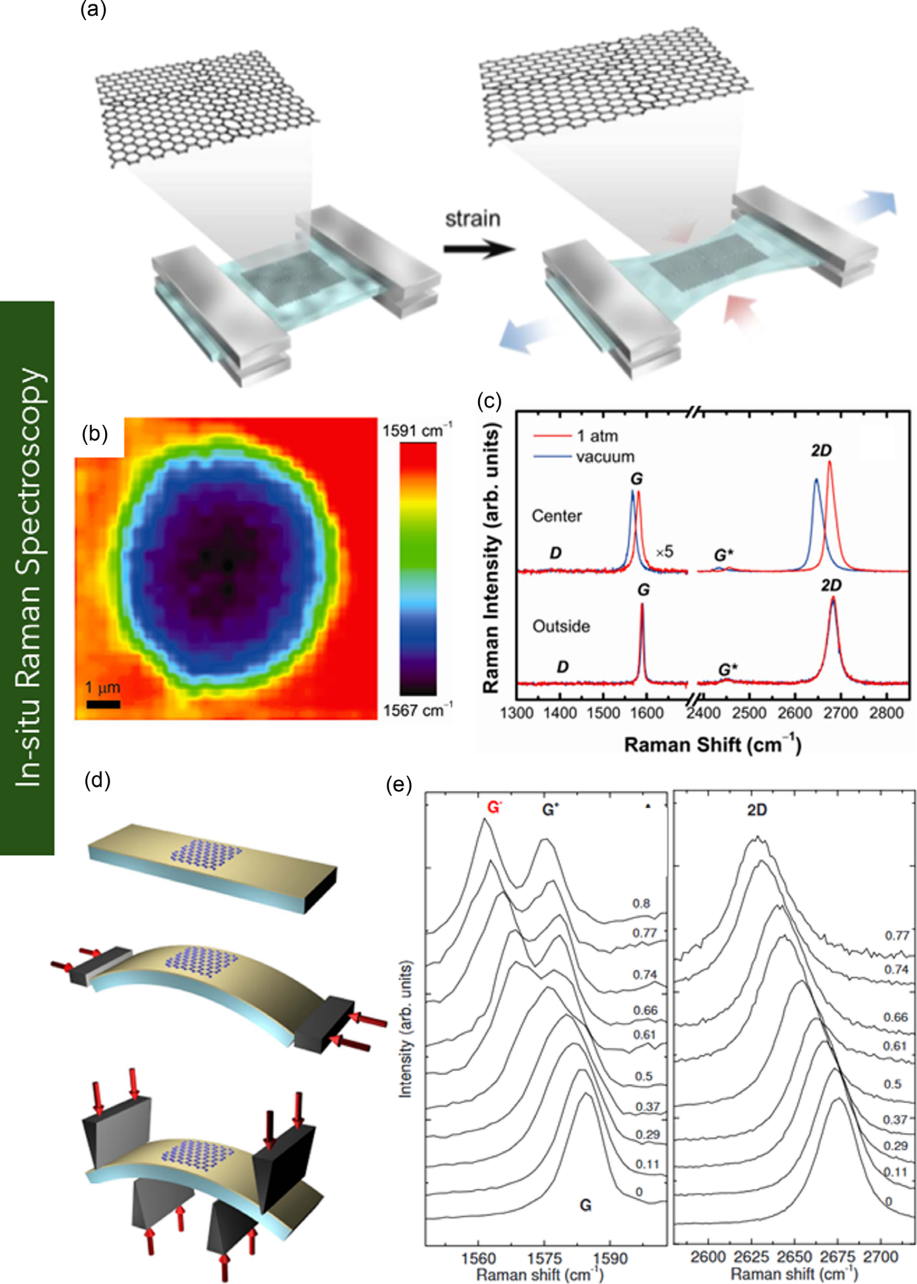
a) Scheme depicting the mechanical strain application to PG using a custom rig. Reproduced with permission.^[^ ^288^ ^]^ Copyright 2012, American Chemical Society. b) Raman mapping of G peak data of MG at a hole diameter of 6.4 μm. c) Raman spectra comparison of suspended graphene membranes at atmospheric and vacuum pressure conditions. (b,c) are reproduced with permission.^[^ ^291^ ^]^ Copyright 2012, American Chemical Society. d) Schematic illustration depicting the experimental setup for uniaxial strain application to MG‐supported polymer substrates (PET & acrylic). e) Raman shifts in the D and 2G bands as a function of uniaxial strain. (d,e) are reproduced with permission.^[^ ^290^ ^]^ Copyright 2009, American Physical Society.

For instance, Bissett et al.^[^ ^288^ ^]^ synthesized PG via CVD and subsequently transferred the films onto flexible polymer substrates, PDMS and PMMA, to investigate the mechanical strain response using confocal Raman spectroscopy. Uniaxial strain was applied using a custom‐fabricated tensile rig, with strain levels calibrated based on the spectral shift of the 2D Raman band (**Figure** **23**a). The maximum applied strain ranged from ≈0.2% to 0.3%, and the corresponding Raman shifts were quantified using the Grüneisen parameter (*γ*_2_ *D* = 2.7). For CVD graphene, the G band exhibited a positive shift of +41.1 cm^−1^/%—contrary to the behavior typically observed in exfoliated graphene—while the 2D band shift remained consistent across all graphene types at –72.3 cm^−1^/%. The Raman spectral analysis revealed peak broadening without evident peak splitting, suggesting an inhomogeneous strain distribution within the graphene films.

Beyond mechanical strain, Raman spectroscopy has also proven to be a powerful tool for probing the mechanical properties of graphene under pressure‐induced deformation,^[^ ^291^ ^]^ particularly through the use of micrometer‐scale tips.^[^ ^292^ ^]^ Weng et al.^[^ ^292^ ^]^ employed tip‐enhanced Raman scattering (TERS) to evaluate the Young's modulus of suspended MG. The CVD‐grown MG was transferred and suspended across a hexagonal aperture with a side length of 34.6 μm. A gold micrometer tip with an apex diameter of 2.3 μm was used to induce localized strain by varying the tip–graphene distance. As the tip approached the graphene surface, the Raman intensities of the G and 2D bands exhibited significant enhancement, following an exponential decay profile and remaining detectable up to a distance of 1.5 μm. To further probe the mechanical response, a DC bias voltage (0–12 V) was applied between the tip and the suspended MG, resulting in voltage‐induced out‐of‐plane deformation. This deformation was accompanied by increased 2D band intensity from 3.3 to 6.3 arbitrary units. By analyzing the Raman intensity data in conjunction with a point‐force model for a circular graphene membrane (60 μm diameter), the authors estimated Young's modulus of MG to be ≈1.48 TPa. This technique can be further employed to investigate the out‐of‐plane mechanical response of 2D materials, which is critical for understanding delamination and wrinkling phenomena that directly influence structural integrity and performance stability.

In addition, MEMS‐based platforms have been effectively utilized to apply uniaxial strain during in‐situ Raman spectroscopy. For instance, Garza et al.^[^ ^293^ ^]^ applied large uniaxial strains exceeding 10% to FLG (three layers) using a thermally actuated in‐plane microactuator (TIM) integrated within a MEMS device. Mechanically exfoliated FLG flakes were transferred onto silicon substrates, and precise clamping was achieved through localized epoxy deposition using a femtopipette. Strain was induced by passing an electric current through the TIM, leading to thermal expansion and the generation of tensile force. Raman spectroscopy was employed to monitor the strain response, with a maximum elongation of 12.5% achieved under an applied force of 1.75 mN. At this strain level, the G band and 2D band exhibited redshifts of −0.24 cm^−1^/% and −0.50 cm^−1^/%, respectively. Notably, this represents the highest uniaxial strain applied to FLG during Raman measurement reported to date. The deformation process was fully reversible and nondestructive, as indicated by the absence of permanent changes in the Raman spectra following strain relaxation.^[^ ^293^ ^]^ In MLG, vibrational coupling between adjacent layers further complicates the strain response. Interlayer interactions shift the intrinsic frequencies of both G and 2D bands and can suppress or modify the splitting behavior seen in monolayer graphene. This highlights the importance of layer number, stacking order, and interlayer registry when interpreting Raman‐derived mechanical parameters. Beyond high strain and precision strain tuning resolution, many MEMS platforms could be used for cyclic loading and unloading or multiphysics actuation, which are critical to study fatigue response, hysteresis behaviors, and thermal‐/electric‐mechanical relationship of 2D materials.

Raman spectroscopy can be integrated with vacuum‐assisted pressure modulation systems and two‐/four‐point bending platforms to characterize the mechanical behavior of graphene nanostructures under controlled deformation. For instance, Lee et al.^[^ ^291^ ^]^ employed a vacuum‐assisted pressure variation system to induce biaxial strain in suspended MG films. Mechanically exfoliated MG was transferred onto Si/SiO_2_ substrates containing circular cavities with diameters ranging from 2 to 7 μm. Upon applying a pressure differential across the membrane within a vacuum chamber, the graphene deformed into a balloon‐like structure, generating biaxial strain at the membrane center. In situ Raman spectroscopy was used to monitor strain through shifts in the G band (Figure 23b). As the hole diameter increased, the G peak exhibited a progressive redshift, corresponding to increasing biaxial strain—ranging from 0.07% (for 3.1 μm diameter) to 0.19% (for 6.4 μm diameter) (Figure 23c). Utilizing the Raman shift data and finite element analysis (FEA), Young's modulus of MG was estimated to be 2.4 ± 0.4 TPa, while BLG exhibited a modulus of 2.0 ± 0.5 TPa. This technique accurately evaluated graphene's mechanical properties under small‐strain conditions.^[^ ^291^ ^]^ Additionally, Mohiuddin et al.^[^ ^290^ ^]^ applied uniaxial strain to MG using two‐point and four‐point bending configurations to study the strain‐induced evolution of Raman features (Figure 23d). MG, prepared via micromechanical cleavage and transferred onto flexible PET and acrylic substrates, was subjected to progressive uniaxial strain up to 1.3%, in increments of 0.05%. Under applied strain, the G band split into two distinct sub‐peaks, G^+^ and G^−^, redshifted with increasing strain at −10.8 cm^−1^/% and −31.7 cm^−1^/%, respectively. The 2D band also redshifted at −64 cm^−1^/% but did not exhibit spectral splitting (Figure 23e). From these shifts, the Grüneisen parameters were extracted, yielding values of 1.99 for the E_2_g^+^ mode and 0.99 for the E_2_g^−^ mode, in agreement with first‐principles calculations. Furthermore, the G peak intensities showed polarization‐dependent behavior, with their relative intensities varying according to the crystallographic orientation of the MG with respect to the strain direction.^[^ ^290^ ^]^

### 4.2 In Situ SEM

In situ SEM serves as a powerful technique for characterizing the mechanical behavior of graphene nanostructures by integrating high‐resolution imaging with real‐time deformation analysis (Figure 22c). When combined with MEMS devices, this setup enables the application of controlled tensile or compressive loads to graphene‐based specimens directly within the SEM chamber.^[^ ^294^ ^]^ MEMS‐based tensile platforms are capable of exerting forces in the nN range with displacement resolutions down to ≈1 nm, facilitating precise acquisition of stress–strain responses in ultrathin graphene structures.^[^ ^295^ ^]^ For in situ SEM, the modulus is not obtained directly from the imaging itself, but from the combination of SEM observation with integrated force and displacement‐sensing platforms such as MEMS devices, PTP stages, or AFM cantilevers.^[^ ^296^ ^]^ While the MEMS or PTP devices supply calibrated force–displacement data, SEM provides real‐time visualization of specimen geometry and deformation progression. The elastic modulus is then calculated from the linear slope of the stress–strain curve, where stress is determined by dividing the applied force (measured from the load cell or cantilever deflection) by the effective cross‐sectional area of the graphene specimen. Strain is determined by displacement normalized by the gauge length, often tracked through SEM imaging or integrated capacitive/displacement sensors.^[^ ^283^, ^297^ ^]^ For example, Wang et al.^[^ ^298^ ^]^ employed a MEMS tensile device to investigate the mechanical properties of GO paper under in situ nanomechanical testing. The elastic modulus of GO in ambient conditions was measured at 7.43 ± 0.18 GPa within the linear elastic regime (up to 1.20% strain), which subsequently decreased to 2.22 ± 0.09 GPa at higher strains due to interlayer slippage between GO sheets. SEM imaging during deformation enables the visualization of structural phenomena such as wrinkle formation, localized buckling, and fracture propagation, offering mechanistic insights into the deformation behavior and failure modes of graphene nanostructures.^[^ ^298^ ^]^

Push‐to‐pull (PTP) devices are also widely utilized in conjunction with in‐situ SEM to investigate the mechanical behavior of graphene nanostructures under controlled loading conditions. For instance, Li et al.^[^ ^299^ ^]^ explored the in‐plane mechanical properties of MLG nanosheets with thicknesses ranging from ≈10 nm (≈10 layers) to 300 nm (≈1000 layers) through in‐situ tensile testing conducted within SEM and transmission electron microscopy (TEM) environments. CVD‐synthesized MLG was mounted onto a Hysitron PTP micromechanical testing platform, which applied uniform uniaxial tensile strain across the sample. The nanosheets were clamped at both ends using conductive silver adhesive to ensure secure electrical and mechanical contact. The results revealed that thinner MLG nanosheets exhibited significantly higher fracture strength (2.59 GPa) and larger fracture strain (25.97%) compared to their thicker counterparts. In contrast, thicker MLG samples demonstrated a lower fracture strength and followed a characteristic three‐stage failure process: an initial flattening phase, elastic deformation, and abrupt brittle fracture. Notably, thinner nanosheets displayed interlayer delamination during fracture, highlighting the influence of thickness on failure mechanisms.^[^ ^299^ ^]^

In addition to MEMS and PTP devices, micro‐tensile testers integrated within the SEM chamber have also been employed for mechanical testing of freestanding graphene. Jang et al.^[^ ^300^ ^]^ used this approach to study the fracture behavior of MG. MG was mechanically exfoliated using the Scotch tape method and subsequently transferred onto a SiO_2_ substrate. A focused ion beam (FIB) was used to introduce a pre‐crack, and the specimen was mounted on a PTP device for uniaxial tensile testing under SEM observation. This enabled real‐time visualization of crack initiation and propagation. Load‐displacement curves showed a stiffness of 510 N m^−1^, and the failure load was measured to be 45.5 μN. It was found that crack propagation occurred along a zigzag direction, with the fracture surface perpendicular to the loading direction. The recorded load–displacement data showed a stiffness of 273 N m^−1^ and a failure load of 45.5 μN. Crack propagation occurred predominantly along the zigzag crystallographic direction, with the fracture surface oriented perpendicular to the tensile axis. The in‐plane Young's modulus was calculated as 273 N m^−1^ (2D representation), corresponding to an equivalent 3D modulus of 816 GPa. The measured fracture toughness was MPa·$\sqrt{m}$, attributed to the intrinsic nonlinear elastic behavior of MG at elevated strain levels.^[^ ^300^ ^]^

**Figure 24:**
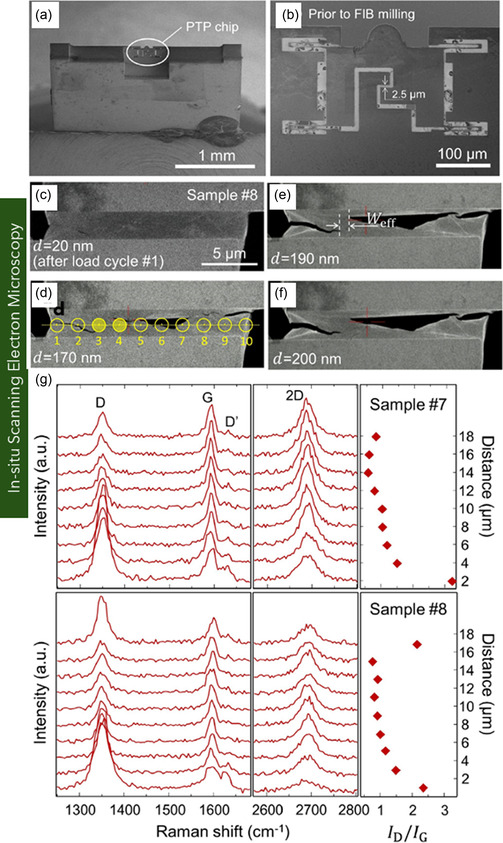
a) SEM of PTP device mounted on SEM holder with a MEMS system. b) The SEM image shows the transferred MG before FIB milling. c–f) SEM images of sequential loading cycles of the PTP test at an indenter displacement of 20, 170, 190, and 200 nm, respectively. g) Raman spectra of FIB cut MG. Reproduced under the terms of CC‐BY license.^[^ ^301^ ^]^ Copyright 2024, The Authors, published by Elsevier Ltd.

SEM integrated with Raman spectroscopy and strain/stress testing platforms provides a robust multimodal approach for probing the mechanical properties of graphene nanostructures. This integrated setup enables simultaneous high‐resolution imaging and chemical‐mechanical analysis, offering valuable insights into the relationship between structural features and mechanical performance. For example, Varillas et al.^[^ ^301^ ^]^ employed a multiprobe technique combining in‐situ SEM and Raman spectroscopy to investigate the mechanical behavior of MG. CVD‐synthesized MG was transferred onto Si substrates using a cellulose nitrate sacrificial polymer layer to facilitate sample handling. The graphene samples were mounted onto a PTP device equipped with AFM cantilevers and subjected to uniaxial tensile loading (**Figure** **24**a,b). Mechanical testing within a strain range of 0 to 2.5% yielded a computed Young's modulus of ≈1.0 TPa. Sequential SEM imaging captured deformation progression (Figure 24c–f), while Raman spectroscopy was used to monitor local strain via the shift of the 2D band. A linear correlation was observed between the applied strain and Raman shift, with a slope of ≈50 cm^−1^ per percent strain (Figure 24g). The fracture strength of pristine MG was reported as 110 GPa, with a maximum tensile stress of up to 130 GPa prior to failure. Furthermore, the study demonstrated that structural imperfections, including grain boundaries and vacancies, significantly impaired the mechanical integrity of MG. Defective regions exhibited a reduction in tensile strength of up to 60%, underscoring the sensitivity of 2D materials to nanoscale defects.^[^ ^301^ ^]^

In addition to evaluating parameters such as strain, elastic modulus, stiffness, failure load, and fracture strength, in situ SEM is also a valuable tool for investigating interfacial strength in nanostructured systems. For example, Roenbeck et al.^[^ ^302^ ^]^ examined the adhesion energy between MWCNTs and graphene by integrating in situ SEM with AFM and molecular mechanics simulations. In their study, MWCNTs were mechanically peeled from a graphene‐on‐Cu substrate using an AFM cantilever within the SEM chamber, while the applied peeling force was quantified by measuring cantilever deflection. The AFM cantilevers employed had well‐characterized stiffness values, enabling precise force calibration. The adhesion or surface energy was calculated using Kendall's peeling model, which relates the peeling force to the peel angle and cantilever deflection. Based on this model, the interfacial adhesion energy was determined to be ≈6.0 nJ m^−1^ per unit length of the MWCNT. The computed surface energy values ranged from 0.20 J m^−2^ for collapsed nanotubes to 0.36 J m^−2^ for flattened MWCNTs, illustrating the significant role of nanotube morphology in interfacial mechanics.^[^ ^302^ ^]^

In SEM‐based mechanical characterization of graphene and related nanostructures, reported error bars arise from both experimental limitations and specimen variability. The error bars typically are generated from three main sources: 1) uncertainties in force calibration of MEMS sensors or AFM cantilevers, 2) variations in specimen geometry such as effective thickness and gauge length (significant for FLG), and 3) image resolution limits in measuring displacement within SEM. To minimize these errors, multiple loading–unloading cycles and repeated tests across different specimens are conducted, with statistical averaging used to define mean modulus values and standard deviations. Thus, modulus values reported in SEM‐based mechanical studies reflect not only the intrinsic response but also the measurement precision of the coupled force–displacement sensing platforms.

Defects play a critical role in governing the mechanical behavior of graphene nanostructures, influencing properties such as strength, stiffness, and fracture mechanisms. In addition to quantitative mechanical characterization, in situ SEM enables direct visualization of defect‐mediated deformation, offering mechanistic insights into failure processes at the nanoscale. Bhatt et al.^[^ ^303^ ^]^ reported that the fracture strength of graphene decreases by ≈17.7% due to the presence of a single atomic vacancy, underscoring the pronounced impact of even minimal lattice imperfections. Grain boundaries also contribute significantly to mechanical performance, with in situ SEM observations revealing that the orientation and structure of grain boundaries strongly affect crack propagation trajectories and interfacial strength. Tensile testing of PG using in situ SEM demonstrated fracture initiation at grain boundaries, with failure stresses ranging from ≈70 to 90 GPa, depending on grain size and defect density.^[^ ^304^ ^]^ Under compressive loading, defective graphene nanostructures exhibit localized wrinkling and buckling, particularly in regions surrounding vacancies and grain junctions. High‐resolution SEM imaging (≈10 nm) during mechanical loading enables the real‐time detection of stress concentrations, bond breakage, and crack nucleation, which corroborate MD simulation predictions of stress distribution and fracture pathways.^[^ ^305^ ^]^ These findings highlight the utility of in situ SEM as a bridge between experimental observation and computational modeling in defect‐sensitive 2D material systems.

### 4.3 TEM

**Figure 25:**
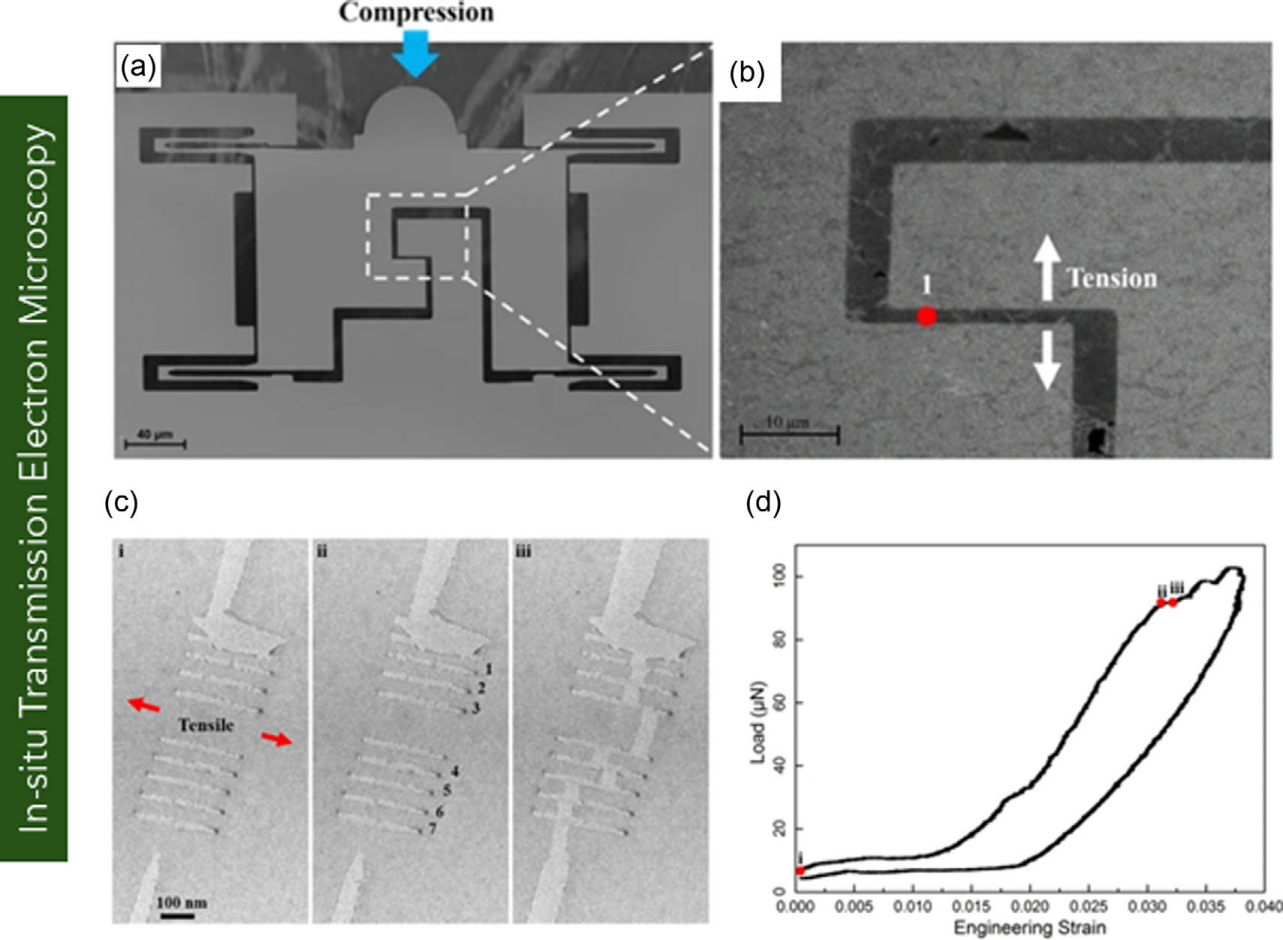
a) SEM image of the PTP device. b) SEM image depicting the successful placement of MG on the PTP device. c) TEM images at i) initial strain, ii) maximum strain, and iii) after GNR fracture. d) Load‐engineering strain curve of GNRs. Reproduced under terms of the CC‐BY license.^[^ ^309^ ^]^ Copyright 2017, The Authors, published by Nature Portfolio.

Compared to SEM, TEM serves as a pivotal technique for investigating the mechanical behavior of graphene nanostructures (Figure 22d). TEM enables direct atomic‐scale visualization of lattice arrangements and defect structures under applied mechanical stress, offering critical insights into deformation mechanisms and fracture pathways.^[^ ^262^ ^]^ In situ mechanical testing within TEM is typically conducted by suspending graphene nanostructures over microfabricated grids and applying load through integrated actuators such as AFM probes or nanoindenters.^[^ ^263^ ^]^ This configuration allows precise evaluation of tensile properties and fracture responses. Moreover, TEM facilitates detailed analysis of defect‐mediated mechanical responses. For example, vacancy defects markedly reduce tensile strength, while Stone–Wales defects introduce local stress concentrations that promote non‐uniform deformation and localized plasticity.^[^ ^265^ ^]^ At the macroscale, the high density of such point defects in CVD‐grown graphene films has been shown to reduce wafer‐scale breaking strength ranging from 98–118 GPa,^[^ ^306^ ^]^ lower than the intrinsic theoretical value of ≈130 GPa. Similarly, grain boundaries—commonly visualized by TEM as arrays of pentagons and heptagons—act as preferential crack initiation sites.^[^ ^307^ ^]^ While isolated grain boundaries can sometimes redistribute strain and blunt cracks, high‐density networks typically degrade mechanical performance, reducing the effective modulus relative to defect‐free exfoliated graphene.^[^ ^308^ ^]^ Thus, TEM observations not only identify defect structures but also provide a mechanistic basis for explaining the reduced strength and fracture toughness of large‐area polycrystalline films used in practical devices. Liao et al.^[^ ^309^ ^]^ examined the mechanical performance of GNRs subjected to uniaxial tension within a TEM chamber. The GNRs were fabricated by patterning CVD‐grown graphene into nanoribbons with widths ranging from 40 to 75 nm using focused electron beam sculpting. The structural integrity and crystallinity of the GNRs were validated via Raman spectroscopy and selected area electron diffraction (SAED). In situ tensile testing was conducted using a Hysitron PicoIndenter (**Figure** **25**a,b), revealing that the GNRs fractured at true strain levels between 2.8% and 3.0%, significantly lower than the theoretical fracture strain of ≈16%. Figure 25c illustrates the morphological evolution before, at peak loading, and after fracture, while Figure 25d presents the corresponding load‐strain profiles under different test conditions. Furthermore, EELS analysis indicated no bandgap opening during deformation. This discrepancy (3% vs. 16%) was attributed to electron‐beam‐induced edge roughness and vacancy generation, which at the wafer scale manifest as edge‐dominated fracture in patterned graphene channels. Complementary DFT simulations further suggested that bandgap opening under strain (>2.2%) would only be feasible for sub‐2 nm GNRs, indicating dimensional and defect constraints that limit strain engineering in macroscale devices. These findings underscore the influence of nanoscale defects and dimensional constraints on the mechanical and electronic performance of GNRs, with implications for their deployment in strain‐engineered nanoelectronic applications.^[^ ^309^ ^]^

In addition to AFM probes and nanoindenters, MEMS devices integrated with TEM have been increasingly utilized to apply controlled strain to graphene nanostructures during in situ mechanical testing. For example, Kim et al.^[^ ^310^ ^]^ investigated the mechanical behavior of MG by performing in situ uniaxial tensile testing within a TEM. MG was mechanically exfoliated from graphite and transferred onto a MEMS device using a dry transfer method. A flat punch probe was employed to apply uniaxial strain during TEM observation. The resulting stress‐strain curves revealed Young's modulus ranging from 89 to 371 GPa and a maximum tensile strength of 22.3 GPa. Crack initiation was observed at folded regions of the graphene, with crack propagation preferentially occurring along zigzag and armchair crystallographic orientations, underscoring the brittle fracture characteristics of MG.^[^ ^310^ ^]^ Hence, even in exfoliated samples, wafer‐scale properties are controlled by mesoscale structural imperfections.^[^ ^310^ ^]^

In another study, Cao et al.^[^ ^311^ ^]^ explored the thickness‐dependent failure strength of GO nanosheets using in situ TEM tensile testing facilitated by a MEMS device fabricated via the silicon‐on‐insulator micromachining process (SOI‐MUMPs). GO nanosheets were drop‐cast onto the MEMS platform, and uniaxial tensile strain was applied using V‐beam thermal microactuators. The thickness of the linked GO nanosheet was determined to be 27 ± 7 nm using electron energy loss spectroscopy (EELS). Mechanical testing revealed a failure strength of 4.4 ± 2.2 GPa, which was lower than that of pristine GO. This reduction in strength was attributed to the presence of carbon‐based linkages, which acted as stress concentrators. Fracture occurred at an applied strain of ≈4%, with crack propagation observed predominantly along the carbon‐linking interfaces.^[^ ^311^ ^]^ At device scales, such weak interfaces manifest as interflake debonding, which compromises the macroscopic toughness of GO membranes.^[^ ^311^ ^]^

In situ TEM has been utilized to characterize the mechanical behavior and lattice evolution of functionalized graphene nanostructures under applied strain. Liu et al.^[^ ^312^ ^]^ investigated the mechanical response of lithiated GNRs using in situ TEM. The GNRs were synthesized by unzipping MWCNTs and subsequently lithiated via electrochemical biasing at −2 V, with lithium metal serving as the counter electrode. Lithiation induced the formation of a Li_2_O surface layer and expanded the interlayer spacing from 3.35 to 3.59 Å. Mechanical testing revealed that, unlike the brittle fracture often observed in MWCNTs, the lithiated GNRs demonstrated exceptional mechanical resilience under tensile and compressive loading, exhibiting no evidence of catastrophic failure.^[^ ^312^ ^]^ The observed Li_2_O surface layer and expanded interlayer spacing suppressed brittle fracture, suggesting that controlled chemical modification may enhance wafer‐scale durability by mitigating vacancy‐driven crack propagation.^[^ ^312^ ^]^

TEM provides critical atomic‐scale evidence of how defects such as vacancies, grain boundaries, folds, and chemical linkages govern the fracture and deformation behavior of graphene. These nanoscale observations correlate directly with the mechanical performance of wafer‐scale graphene membranes and devices, explaining the discrepancy between theoretical ideal strengths (≈130 GPa) and lower experimentally measured values (typically ≈118 GPa). Thus, TEM not only characterizes intrinsic nanoscale mechanisms but also bridges them to macroscopic reliability in large‐area graphene applications.

### 4.4 AFM

#### 4.4.1 Atomic Force Microscopy: Nanoindentation

AFM nanoindentation is a high‐resolution technique that integrates the principles of AFM with nanoindentation to quantitatively assess the mechanical properties of materials at the nanoscale (Figure 22e). This method involves applying a controlled force—typically ranging from 1 nN to 10 μN—via a sharp indenter tip affixed to the end of a compliant cantilever, which is brought into contact with the sample surface.^[^ ^313^ ^]^ By precisely measuring the indentation depth in response to the applied force, key mechanical parameters such as hardness, elastic modulus, and adhesion forces can be extracted.^[^ ^294^ ^]^ The underlying mechanics of AFM nanoindentation are governed by the localized interaction between the tip and the sample surface. As the tip indents the material, a force–displacement (F–h) curve is generated, which serves as the basis for deriving mechanical properties using established contact mechanics models.^[^ ^314^ ^]^ The indenter, typically fabricated from diamond or silicon with a well‐defined geometry, transmits the load to the sample, and the resulting deformation is monitored with sub‐nanometer precision. The relationship between the applied force *F* and the indentation depth *h* can be expressed analytically, as shown in Equation (9).

(9) F=k⋅h

where *k* is the sample stiffness under indentation and *h* is the penetration depth. The F–h relationship obtained during AFM nanoindentation reflects the mechanical response of the material, particularly within the elastic regime prior to the onset of plastic deformation. As the indenter penetrates the sample surface, the material initially undergoes elastic deformation, which is reversible. With increased loading, plastic (irreversible) deformation may also occur depending on the mechanical characteristics of the material.^[^ ^314^ ^]^ During the loading phase, the applied force increases either linearly or nonlinearly, influenced by the material's elastic and plastic response. Upon unloading, the force decreases, and the slope of the initial portion of the unloading curve corresponds to the contact stiffness of the material. The elastic modulus *E* is commonly determined from the unloading segment of the F–h curve using the Oliver–Pharr method.^[^ ^315^ ^]^ In this method, the indentation depth *h* is related to the applied load *F* within the elastic regime, and the reduced modulus is extracted based on the contact stiffness (Equation 10).

(10) $$E=\frac{1}{2}\frac{S}{\sqrt{A}}$$

where *A* is the projected contact area of the indenter and *S* is the contact stiffness, which can be obtained from the unloading slope (dF/dh).

**Figure 26:**
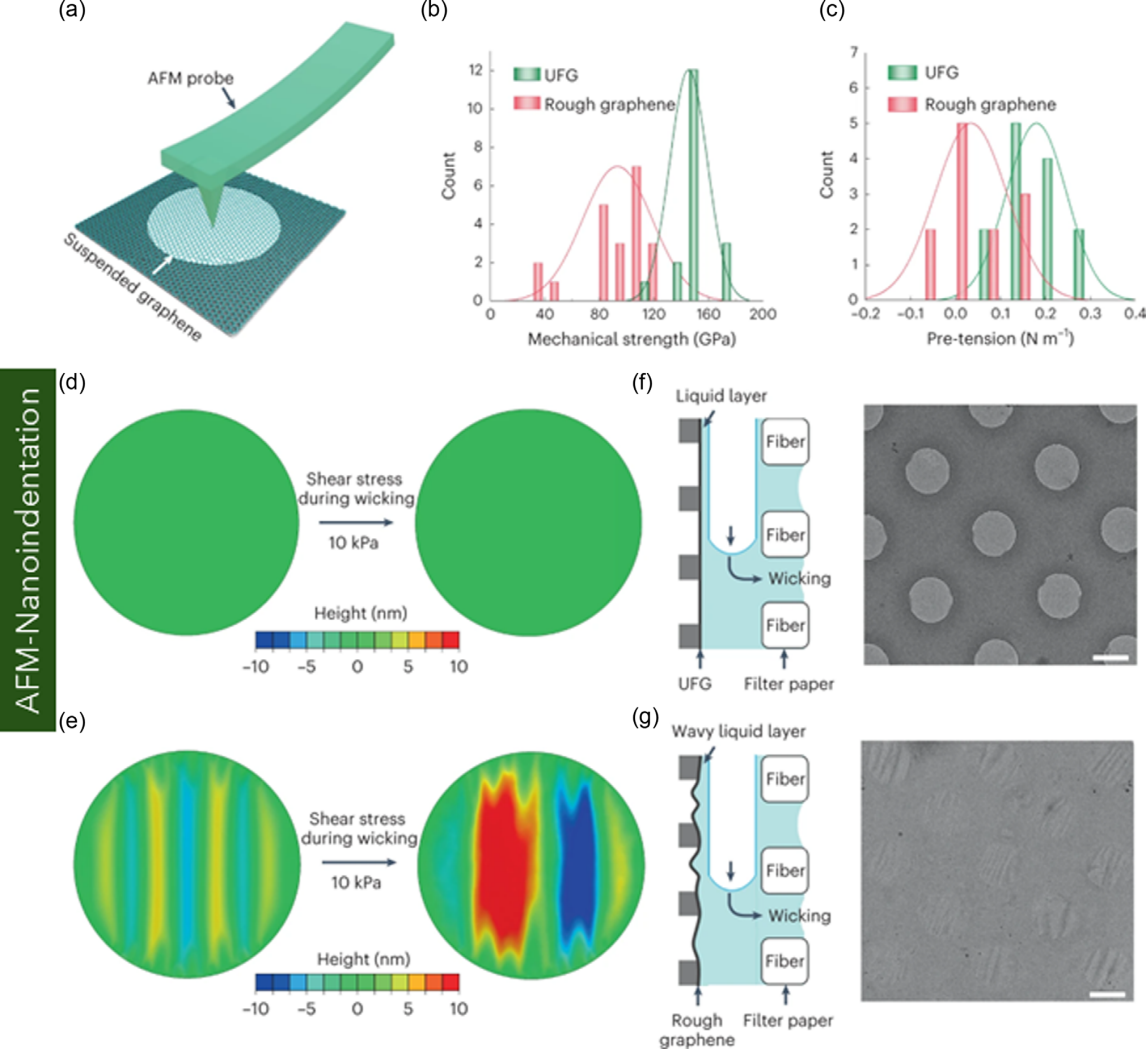
a) Schematic illustration of AFM nanoindentation performed on suspended MG. b,c) Histograms showing the distribution of mechanical strength values for suspended MG. d) Finite element simulation depicting the deformation behavior of suspended MG without applied pretension. e) Finite element simulation of suspended MG under a pretension of 0.2 N m^−1^. f) Schematic representation of the wicking process and corresponding cryo‐EM images demonstrating the absence of ice formation. g) Schematic and cryo‐EM images illustrating successful ice formation on suspended MG. Reproduced under terms of the CC‐BY license.^[^ ^319^ ^]^ Copyright 2023, The Authors, published by Nature Portfolio.

In addition to stiffness, AFM nanoindentation is widely utilized to quantify the elastic modulus and mechanical strength of graphene nanostructures with high spatial resolution. For instance, Lee et al.^[^ ^316^ ^]^ investigated the elastic and frictional properties of MG and FLG (three layers) through AFM‐based nanoindentation and friction force microscopy (FFM). Graphene samples were mechanically exfoliated and transferred onto SiO_2_ substrates featuring circular wells, which were fabricated using nanoimprint lithography. AFM nanoindentation was employed to probe the mechanical response of freestanding graphene membranes. The measured 2D Young's modulus of MG was 342 N m^−1^, corresponding to a 3D bulk modulus of ≈1.02 TPa. FFM measurements revealed a decrease in frictional forces with increasing graphene thickness, attributed to enhanced in‐plane stiffness and reduced surface interaction in FLG compared with MG. The study also highlighted the nonlinear elastic behavior of graphene under large strains, with MG exhibiting a breaking strength of ≈42 N m^−1^ (≈130 GPa in bulk), which diminished with increasing layer thickness.^[^ ^316^ ^]^ Zandiatashbar et al.^[^ ^317^ ^]^ further explored the influence of structural defects on the mechanical integrity of MG using AFM nanoindentation. MG sheets were mechanically exfoliated and suspended over circular holes on prefabricated SiO_2_ substrates. Controlled defect generation was introduced via oxygen plasma treatment to create sp^3^‐type and vacancy‐type defects. The study found that sp^3^‐type defects, even when spaced as closely as 5 nm, had minimal impact on the in‐plane stiffness of graphene. In contrast, increasing the density of vacancy‐type defects significantly reduced mechanical performance, with stiffness decreasing by ≈30% relative to pristine MG. Similarly, fracture strength declined with defect incorporation, with sp^3^‐type defects reducing strength by ≈14%, while vacancy defects led to a more pronounced reduction, emphasizing the critical role of defect type and distribution in governing the mechanical robustness of graphene.^[^ ^317^ ^]^ Rasuli et al.^[^ ^318^ ^]^ investigated the elastic modulus of FLG cantilevers using AFM nanoindentation. Circular wells were fabricated on Si substrates via photolithography followed by reactive ion etching, onto which mechanically exfoliated FLG was transferred to form suspended cantilever structures. Force–displacement data obtained through AFM nanoindentation enabled extraction of both elastic modulus and interfacial shear forces. By applying the Derjaguin–Müller–Toporov (DMT) contact mechanics model, the researchers reported a Young's modulus of ≈37 GPa for the FLG cantilevers, and an in‐plane modulus of ≈0.7 TPa. The shear force required to deform the cantilever was also quantified, yielding a value of 5.1 μN, with a corresponding value of 6.4 N m^−1^ reported for MG.^[^ ^318^ ^]^ Zheng et al.^[^ ^319^ ^]^ further explored the mechanical properties of ultraflat graphene (UFG) using AFM nanoindentation (**Figure** **26**a). MG was synthesized on Cu(111)/sapphire wafers and subsequently transferred onto electron microscopy (EM) grids to achieve ultraflat, wrinkle‐free graphene films. AFM nanoindentation was conducted using a diamond tip with a ≈10 nm radius to probe the mechanical behavior of UFG. The measured Young's modulus was 933 ± 171 GPa, and the tensile strength was 145 ± 13 GPa, values comparable to those of mechanically exfoliated graphene (Figure 26b,c). The study revealed that UFG exhibited enhanced resistance to deformation relative to rough graphene, which displayed reduced mechanical strength and increased surface wrinkling. Finite element simulations (Figure 26d,e) indicated that a pretension of 0.2 N m^−1^ in the UFG contributed to maintaining its planar geometry. This structural uniformity is particularly advantageous for cryogenic electron microscopy (cryo‐EM), as it supports the formation of a uniform vitrified ice layer critical for high‐resolution biomolecular imaging. The presence of UFG improved ice quality and particle distribution on EM grids, resulting in enhanced cryo‐EM image resolution (Figure 26f,g). These findings underscore the dual functionality of UFG in mechanical reinforcement and structural optimization for advanced imaging applications.^[^ ^319^ ^]^

The integration of AFM with Raman spectroscopy offers a powerful approach for investigating the mechanical properties of graphene nanostructures by enabling simultaneous force application and strain characterization. For example, Won et al.^[^ ^320^ ^]^ examined the mechanical response of suspended MG under high biaxial strain using AFM nanoindentation in conjunction with in situ Raman spectroscopy. MG was mechanically exfoliated and transferred onto quartz substrates containing circular cavities, resulting in suspended graphene membranes. A custom‐designed AFM probe with a central aperture was employed to apply a localized point load, inducing biaxial strain on the suspended graphene, while Raman spectroscopy was used to quantify the resulting strain via shifts in the G and 2D Raman peaks. The study reported substantial peak shifts under strain—282 cm^−1^ for the G band and 684 cm^−1^ for the 2D band—which represent some of the largest Raman shifts observed in strained graphene to date. A linear correlation was established between the applied strain and Raman shift, with measured sensitivities of 50 cm^−1^/% strain for the G peak and 126 cm^−1^/% strain for the 2D peak. The maximum biaxial strain of 6.1% was achieved under a load of 27.8 μN. These results demonstrate that AFM nanoindentation coupled with Raman spectroscopy enables precise, nondestructive quantification of mechanical strain in graphene nanostructures, providing critical insights into their elastic behavior and strain transfer mechanisms.^[^ ^320^ ^]^

#### 4.4.2 Atomic Force Microscopy: Finite Element Analysis

**Figure 27:**
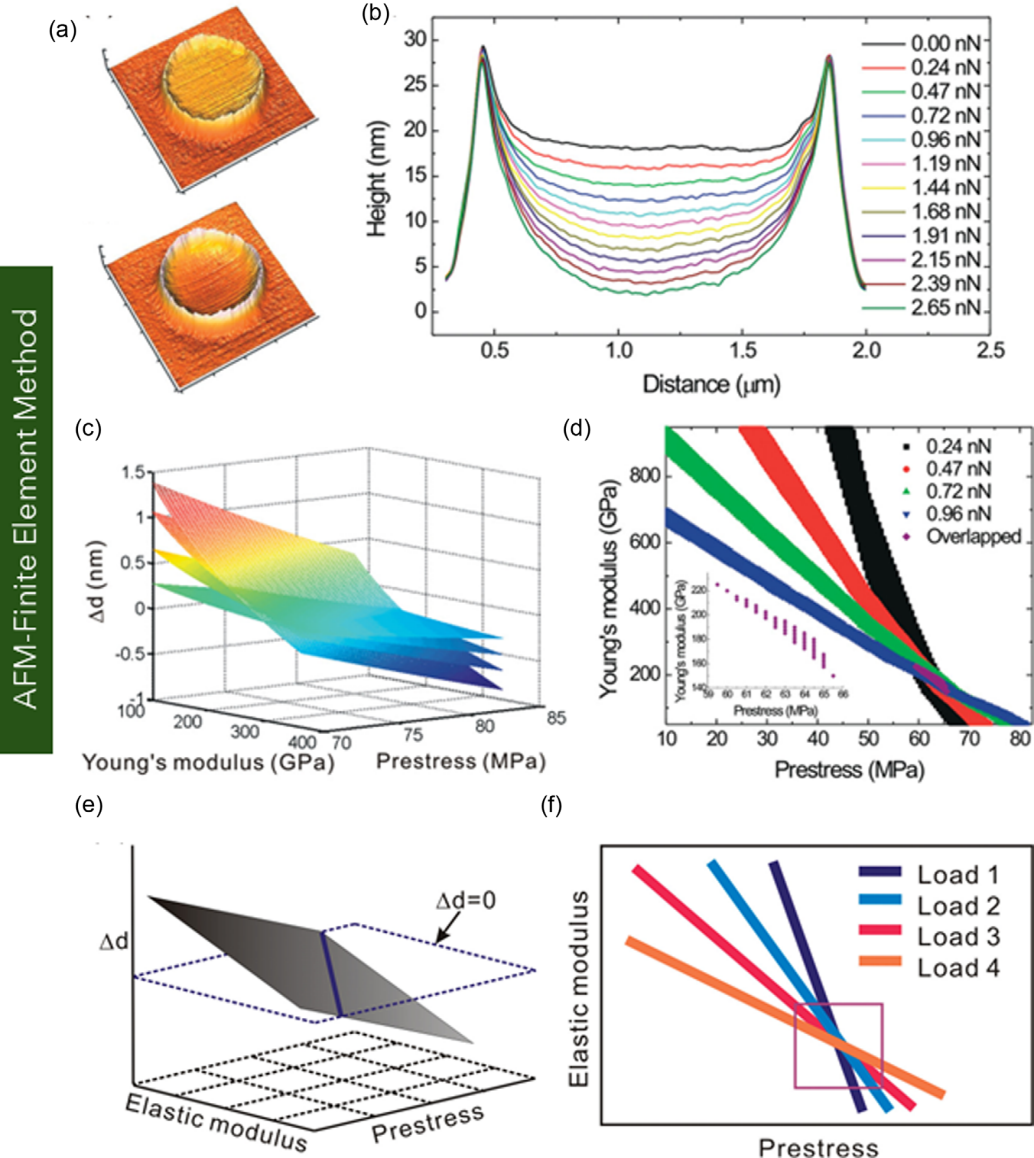
a) 3D AFM height images of monolayer GO under applied loads of 0 and 2.65 nN. b) Cross‐sectional Z‐profile of the GO membrane under different loading conditions. c) 3D displacement difference maps (Δ*d* = *d*_FEM_ − *d*_measured_) at applied loads of 0.24, 0.47, 0.72, and 0.96 nN. d) 2D mapping of effective Young's modulus versus prestress values under varied mechanical loading. e) 3D visualization of displacement differences (Δ*d*) between experimental and FEA‐predicted displacements at a representative loading condition. f) 2D representation of the correlation between extracted Young's modulus and prestress across multiple loading conditions. Reproduced with permission.^[^ ^161^ ^]^ Copyright 2010, American Chemical Society.

AFM and FEA are often integrated to investigate the mechanical behavior of graphene‐based nanostructures by correlating experimental data derived from F–h curves with computational simulations. AFM provides direct nanoscale measurements of mechanical responses, while FEA enables theoretical modeling of stress–strain distributions, accounting for structural defects, geometric constraints, and chemical modifications. Experimental data acquired via AFM can be used to calibrate FEA models, thereby enhancing the predictive accuracy of mechanical performance under applied loads. For instance, Suk et al.^[^ ^161^ ^]^ employed a combined AFM‐FEA approach to examine Young's modulus and intrinsic prestress of monolayer GO nanosheets. GO was synthesized via a modified Hummer's method and subsequently deposited onto quantifoil carbon support films (TEM grids) to facilitate AFM‐based mechanical indentation. As shown in **Figure** **27**a, the suspended GO membranes were subjected to normal forces ranging from 0 to 2.65 nN. The resulting F‐h curves were analyzed to determine the effective Young's modulus, which was reported as 207.6 ± 23.4 GPa (Figure 27b–d)—significantly lower than that of MG, typically ≈1 TPa. Additionally, the prestress within the membranes was quantified at 76.8 ± 19.9 MPa. Membrane deformation exhibited a clear thickness dependence, with thinner regions undergoing higher localized strains (Figure 27e,f). The reduced stiffness of GO, compared to pristine graphene, was attributed to the presence of oxygen‐containing functional groups such as –OH and epoxide moieties, which disrupt the sp^2^ carbon lattice and weaken load transfer efficiency. Complementary FEA simulations were conducted to model elastic deformation behavior, revealing that the degree of oxidation and surface functionalization significantly alters the mechanical response at the nanoscale.^[^ ^161^ ^]^ Similarly, Cao et al.^[^ ^321^ ^]^ deposited GO flakes onto silicon nitride TEM grids featuring 2.5 μm circular holes to investigate their mechanical properties via AFM‐based nanoindentation. The measured breaking load of the suspended GO membranes was 324 ± 121 nN, corresponding to an average tensile strength of 24.7 GPa—≈50% of the intrinsic strength of pristine MG. FEA simulations were subsequently performed and yielded comparable results, validating the experimental observations. From the FEA results, the effective elastic modulus of the 2D GO membranes was determined to be 269 ± 21 N m^−1^, and the intrinsic prestress was estimated at 91 ± 10 mN m^−1^. In parallel, first‐principles DFT calculations were conducted to assess the theoretical strength of GO under biaxial tension, which was predicted to be 31.3 GPa. The close agreement between the DFT predictions and AFM‐derived experimental data underscores the robustness of the combined experimental‐computational framework for evaluating the mechanical performance of functionalized graphene derivatives.^[^ ^321^ ^]^

Both studies employed FEA to extract the mechanical properties of GO membranes from AFM nanoindentation, but their approaches differ in how the continuum representation of an atomically thin sheet was handled. In Suk et al.^[^ ^161^ ^]^'s work, the focus was on mapping elastic modulus and prestress by fitting simulated displacements to experimental AFM data, with bending stiffness explicitly included to capture the enhanced out‐of‐plane rigidity imparted by oxygen functionalization. In contrast, Cao et al.'s^[^ ^321^ ^]^ study emphasized the correlation between indentation load and in‐plane Cauchy stress to derive membrane strength, adopting a uniform effective thickness of 0.7 nm for monolayer GO.

These approaches highlight the central issue of representing a single atomic layer within continuum plate theory. In both cases, graphene oxide was modeled as an isotropic elastic plate of finite thickness, an assumption supported by AFM step‐height measurements (≈0.7 nm) and widely used in prior literature. The inclusion of bending rigidity is justified by the non‐negligible resistance to out‐of‐plane deformation, arising from π–orbital overlap in pristine graphene and further reinforced by oxygen bonding in GO. Poisson's ratio was consistently taken as 0.165 and frictionless tip–membrane contact was assumed. Thus, while atomically thin, GO membranes can be validly treated within continuum mechanics by assigning effective thickness and bending stiffness, enabling direct comparison between FEA simulations and experimental indentation data.

Beyond the characterization of Young's modulus, stiffness, and fracture strength, AFM can also be employed to manipulate graphene nanostructures at the nanoscale, providing insights into their wear behavior and structural response to mechanical deformation. Vasić et al.^[^ ^322^ ^]^ explored wrinkle formation and fracture mechanisms in MG using AFM‐based lateral manipulation combined with force measurements. Mechanically exfoliated MG was transferred onto Si/SiO_2_ substrates, and its edges were mechanically engaged using an AFM tip. During lateral manipulation, a progressive increase in lateral force was recorded as the AFM probe interacted with the graphene edges, resulting initially in elastic deformation, followed by the onset of plastic deformation manifested as wrinkle formation. Wrinkle initiation occurred at forces ranging from 59 to 99 nN, while complete edge fracture and delamination were observed at ≈118 nN.^[^ ^322^ ^]^ Similarly, Giesbers et al.^[^ ^323^ ^]^ utilized AFM probes to manipulate exfoliated MG flakes deposited on SiO_2_ and GaAs substrates. Using contact‐mode lateral manipulation, the AFM tip could move, tear, and fold the graphene sheets, demonstrating its utility in nanoscale mechanical processing. Furthermore, local anodic oxidation (LAO) was performed by applying a 25 V bias between the conductive AFM tip and the graphene surface, resulting in the formation of nanoscale grooves with widths as narrow as 30 nm. This technique enabled precise nanoscale patterning of graphene, highlighting the potential of AFM‐based manipulation and LAO for tailoring graphene architectures in device fabrication and nanomechanical applications.^[^ ^323^ ^]^

### 4.5 STM

In addition to AFM, scanning tunneling microscopy (STM) can be leveraged to manipulate graphene nanostructures at the atomic level. By precisely controlling the tip's position and the applied bias voltage, customizable nanostructures could be obtained. STM can be utilized for flattening nanoridges in graphene. For example, Xu et al.^[^ ^324^ ^]^ utilized STM to manipulate the MLG surface. The epitaxially grown graphene on a 4 H‐SiC(0001) substrate had bunched nanoridges ≈0.1 nm high. Using electrostatic‐manipulation STM (EM‐STM), the nanoridge morphology can be altered. By varying the bias voltage of the STM tip while maintaining a constant tunneling current, a constant force could be applied to the grounded MLG. It was found that a force of up to ≈5 nN could be exerted by the tip, and an energy of around 10 eV was required to alter the geometry of a ≈100 × 200 nm^2^ area. The nanomanipulation resulted in both reversible and irreversible local changes to the morphology of the nanoridges.^[^ ^324^ ^]^ Alyobi et al.^[^ ^325^ ^]^ mechanically manipulated FLG and MLG and inquired about how the applied voltage affected structures. The group utilized an approach‐retraction method to repeatedly stretch the FLG and MLG membranes suspended over voids. A hysteresis response was observed, which was dependent on the voltage applied. The nanomanipulation was driven by the combined interplay between VdW and electrostatic forces. While the suspended MLG membrane could be stretched by 80 nm, the supported portion could only be stretched up to 20 nm owing to substrate‐graphene interactions. It was also reported that electrostatic forces may dominate at high tip voltage, but VdW forces become more prominent at low tip voltage. Such a study provides crucial insights into nanomanipulating graphene nanostructures and strain engineering of 2D material systems.^[^ ^325^ ^]^

### 4.6 Computational Modeling

Simulation and computational techniques play a critical role in advancing the understanding of graphene's mechanical properties by enabling atomic‐scale resolution beyond the capabilities of experimental methods (Figure 22f). These approaches allow the exploration of extreme loading conditions, optimization of experimental parameters, and bridging across spatial and temporal scales. The most widely employed computational methods for investigating the mechanical behavior of graphene nanostructures include MD,^[^ ^326^ ^]^ DFT,^[^ ^327^ ^]^ and continuum mechanics.^[^ ^328^ ^]^ Among these, MD simulations are particularly prevalent due to their ability to model atomic‐scale interactions using empirical interatomic potentials. Commonly used potentials include the Tersoff, reactive empirical bond order (REBO), adaptive intermolecular reactive empirical bond order (AIREBO), and ReaxFF potentials, which capture bond formation, breaking, and nonlinear mechanical responses under various loading conditions.^[^ ^329^ ^]^ MD is especially well‐suited for studying key mechanical parameters such as elastic modulus, fracture strength, and failure strain. In contrast, DFT offers a first‐principles, quantum mechanical framework capable of delivering highly accurate predictions of mechanical and electronic properties at small scales. However, the significant computational cost of DFT restricts its application to relatively small systems, limiting its scalability for large or defect‐rich graphene models.^[^ ^330^ ^]^ Additionally, peridynamics (PD) has recently emerged as a promising computational approach for simulating fracture behavior in graphene nanostructures. Unlike classical continuum mechanics, PD is capable of modeling discontinuities such as cracks without requiring additional failure criteria or remeshing, thereby offering an accurate and robust framework for simulating damage evolution in 2D materials.^[^ ^331^ ^]^

**Figure 28:**
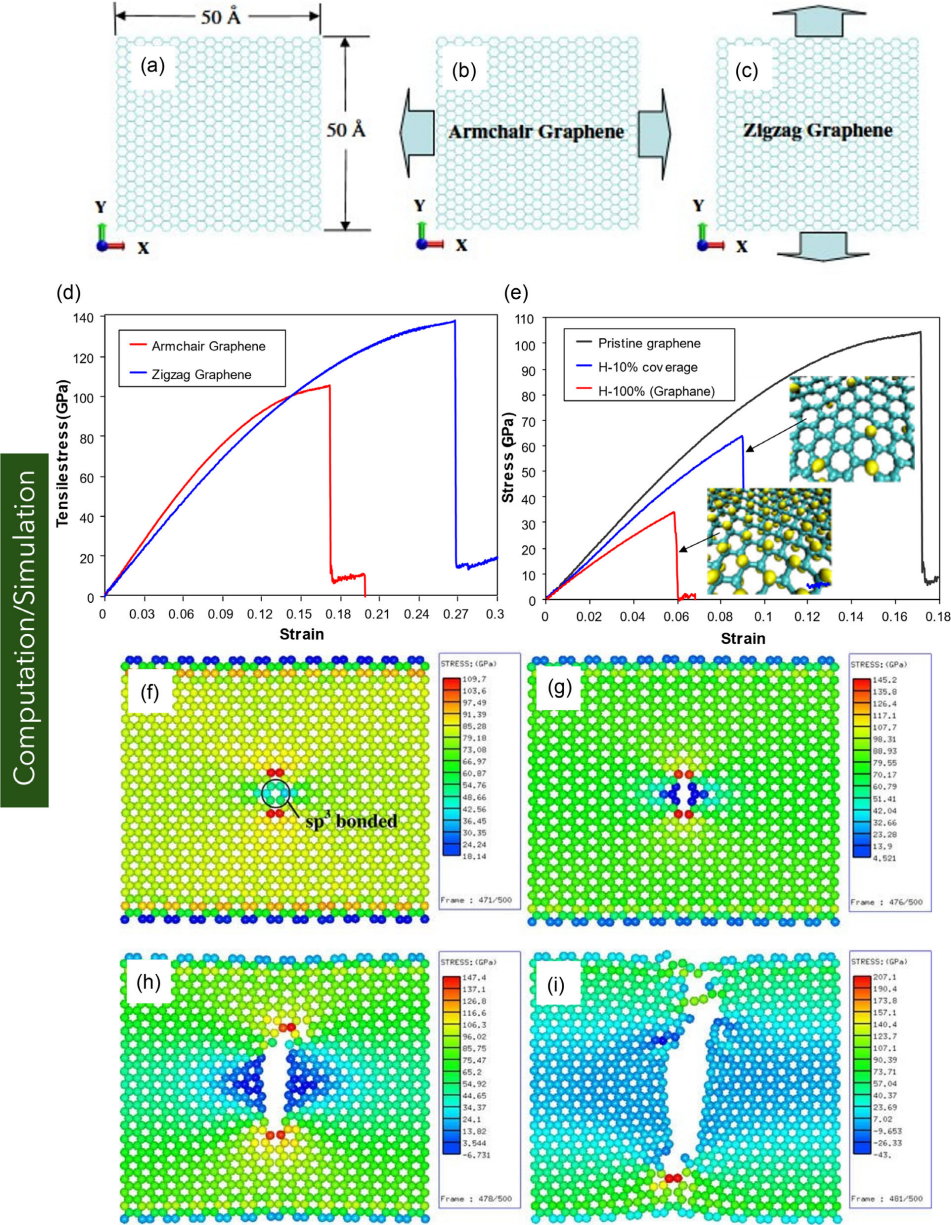
a) Schematic representation of GNR dimensions. b) Armchair‐edged GNR subjected to tensile force. c) Zigzag‐edged GNR under tensile deformation. d) Stress–strain responses of pristine GNRs along the zigzag and armchair directions. e) Stress–strain behavior of hydrogen‐functionalized GNRs. f) Stress distribution profile prior to bond rupture. g) Initiation of bond breaking associated with sp^3^ hybridization. h) Propagation of cracks within the GNR structure. i) Final fracture morphology of the GNR. Reproduced with permission.^[^ ^332^ ^]^ Copyright 2009, Elsevier Ltd.

MD simulations have been extensively employed to investigate the fracture behavior and crack propagation characteristics of graphene nanostructures. For instance, Pei et al.^[^ ^332^ ^]^ explored the influence of hydrogen functionalization on the mechanical properties of GNRs. The study examined hydrogen coverage ranging from 0% (pristine graphene) to 100% (graphane), subjecting both armchair and zigzag GNRs to uniaxial tensile loading along the X and Y directions at a strain rate of 0.0004 ps^−1^ (**Figure** **28**a–c). For unmodified GNRs, Young's modulus was ≈0.86 TPa, with a tensile strength of 121 GPa and a fracture strain of 0.22. In contrast, fully hydrogenated GNRs (graphane) exhibited substantially diminished mechanical performance: Young's modulus decreased to 0.61 and 0.59 TPa for the armchair and zigzag configurations, respectively, while tensile strength dropped to 34.9 GPa (armchair) and 56.9 GPa (zigzag). Fracture strain was also markedly reduced to 0.059 and 0.114 in the armchair and zigzag directions, respectively. The stress–strain responses before and after hydrogenation are illustrated in Figure 28d,e. The degradation in mechanical properties with increasing hydrogen coverage—particularly at low coverage levels (≤30%)—was primarily attributed to the transition from sp^2^ to sp^3^ bonding and the increased rotational freedom of sp^3^‐hybridized carbon atoms within the 2D structure (Figure 28f–i).^[^ ^332^ ^]^

Beyond GNRs, MD simulations have also been applied to model the mechanical behavior of graphene‐reinforced polymer nanocomposites. Lin et al.^[^ ^333^ ^]^ investigated the temperature‐dependent mechanical response of MLG‐reinforced PMMA composites. The Young's modulus (*E*_11_) of a composite containing 3 wt% MLG was found to decrease from 36.538 GPa at 300 K to 31.926 GPa at 500 K. Similarly, the shear modulus (G_12_) (a measure of a material's elastic shear stiffness) declined slightly with increasing temperature, from 11.388 GPa at 300 K. For MG, Young's modulus was reported as 1812 GPa, 1769 GPa, and 1748 GPa at 300 K, 400 K, and 500 K, respectively. Interestingly, the shear modulus (G_12_) for MG was 683 GPa at 300 K and showed a marginal increase with rising temperature. These results underscore the dual impact of both filler content and temperature on the mechanical properties of graphene‐based composites, where higher graphene volume fractions enhance stiffness but also render the materials more susceptible to thermal degradation.^[^ ^333^ ^]^ Furthermore, Javvaji et al.^[^ ^334^ ^]^ examined the synergistic effects of domain size, lattice orientation, and initial crack length on the mechanical performance of graphene sheets with a lattice constant of 2.49 Å. The MD simulations revealed that yield strength was inversely correlated with domain size. Specifically, at a domain size of 10 nm and an initial crack length of 0.1 L, the yield strength was 122.098 GPa, whereas it decreased to 7.091 GPa for an 86 nm domain size. The lattice orientation also played a significant role, with the yield strength dropping from 122.098 GPa at 0° to 78.482 GPa at 13.9°, and further to 12.210 GPa at 30°. Additionally, increasing the normalized crack length from 0.1 to 0.6 L reduced yield strength from 122.098 to 49.535 GPa. These findings collectively highlight the critical role of structural parameters such as domain size, crystallographic orientation, and defect dimensions in governing the mechanical integrity of graphene.^[^ ^334^ ^]^

DFT has been widely employed to investigate the mechanical behavior of graphene due to its quantum‐level accuracy. Memarian et al.^[^ ^335^ ^]^ evaluated the Young's modulus of MG using both MD and DFT approaches. Within the MD framework, various interatomic potentials were tested, including AIREBO, Tersoff, and EDIP‐Marks, due to their capability to accurately model bond lengths and elastic responses. The calculated Young's modulus values were 1023.95 GPa along the zigzag direction and 1022.01 GPa along the armchair direction. In contrast, DFT simulations employing the generalized gradient approximation (GGA) and local density approximation (LDA) yielded equilibrium bond lengths of 1.414 and 1.426 Å, respectively. The corresponding Young's modulus values were slightly higher than those from MM simulations, with GGA and LDA producing 1079.26 and 1084.84 GPa, respectively. These computational results exceeded the experimental benchmark (≈1000 ± 100 GPa), likely due to idealized modeling assumptions. Among the tested potentials, the EDIP‐Marks potential demonstrated the best agreement with experimental data, offering an optimal balance between computational efficiency and predictive accuracy for structural and mechanical properties.^[^ ^335^ ^]^ In a complementary study, Aparicio et al.^[^ ^336^ ^]^ examined the mechanical behavior of finite‐sized GNRs using MD simulations with multiple empirical potentials and validated the findings via DFT. Zigzag‐edged GNRs (zGNRs) were subjected to uniaxial tensile deformation along the *x*‐axis, with hydrogen passivation applied at the ribbon edges to stabilize the structure. Three different potentials—ReaxFF, AIREBO, and a screened reactive empirical bond order (REBO‐scr)—were utilized to assess mechanical responses. For a 7 × 7 zGNR configuration, Young's modulus was found to be ≈0.9 TPa using REBO‐scr and AIREBO potentials, decreasing to ≈0.7 TPa for narrower ribbons. In contrast, the ReaxFF potential predicted an increasing trend in Young's modulus with ribbon width, reaching as high as 1.2 TPa for larger GNRs. Fracture strain was also potential‐dependent, with REBO‐scr yielding ≈2.5% and AIREBO slightly higher at ≈3.0%. DFT simulations corroborated the mechanical behavior predicted by REBO‐scr, particularly in the elastic and fracture regimes. The fracture mode was predominantly brittle, with a fracture stress of ≈99.3 GPa. These results underscore the significant impact of potential selection on mechanical predictions and highlight REBO‐scr as a suitable choice for capturing the fracture characteristics of finite‐sized GNRs with good agreement to first‐principles calculations.^[^ ^336^ ^]^

A relatively new technique, PD, was utilized to investigate the layer‐dependent fracture mechanics of graphene nanostructures. Liu et al.^[^ ^337^ ^]^ utilized PD simulations to examine the fracture behavior of MLG by incorporating both intralayer and interlayer interactions. The inter‐layer model specifically accounted for vdW forces between adjacent graphene layers, enabling the simulation of shear slip and relative atomic displacements. A bond‐based damage criterion was implemented, whereby bonds irreversibly break upon exceeding a critical stretch threshold, thus capturing crack initiation and propagation. The study reported mode I critical stress intensity factors (K_1_c) of 6.63, 6.2, 6.3, and 6.7 MPa $\sqrt{m}$ for monolayer, bilayer, trilayer, and five‐layer graphene sheets, respectively. For MG, the fracture stress was determined to be 21 GPa. In trilayer graphene, a 53.4% reduction in fracture stress was observed as the precrack length increased from 25 to 150 nm, highlighting the influence of flaw size on mechanical integrity. Furthermore, the strain energy density exhibited an increasing trend with the number of graphene layers, which was attributed to asynchronous crack propagation and interlayer slippage. The fracture strain for MG was ≈2.4%, with higher fracture strains observed for multilayer systems. Additionally, an increase in precrack tip radius resulted in a 29.6% enhancement in fracture stress. These results underscore the significant impact of layer number, precrack length, and crack tip geometry on the fracture toughness and mechanical performance of multilayer graphene nanosheets.^[^ ^337^ ^]^

Future research in the mechanical simulation of graphene and other 2D materials is expected to focus on the development of hybrid or coupled frameworks, such as MD–PD, PD–FEA, and integrated DFT/MD/PD models, to bridge multiple length and time scales. Additionally, emerging directions include simulating coupled thermomechanical failure mechanisms and incorporating data‐driven and ML‐enhanced approaches to improve predictive accuracy and computational efficiency.

### 4.7 Interferometry

Interferometry is a noncontact, high‐resolution experimental technique widely employed to characterize the mechanical properties of 2D films (Figure 22g). It enables precise measurement of surface deformations and mechanical responses under external stimuli.^[^ ^338^ ^]^ Optical interferometry and laser Doppler vibrometry (LDV) are among the most frequently utilized methods for quantifying key mechanical characteristics of thin films, including Young's modulus, mechanical resonance, and fracture behavior.^[^ ^339^ ^]^ Fundamentally, interferometry operates by analyzing interference patterns formed from reflected light waves on the sample surface. These patterns are highly sensitive to nanoscale surface displacements and vibrational amplitudes, allowing for exceptionally accurate mechanical characterization. For instance, Ushakov et al.^[^ ^340^ ^]^ employed single‐beam interferometry to investigate the piezoelectric response of CVD‐grown FLG transferred onto silicon nitride membranes with a thickness of 20 nm. A laser interferometer was used to detect displacements induced by an externally applied electric field. At the system's fundamental resonance frequency (≈15 kHz), the graphene‐coated membranes exhibited displacements of up to 14 nm under a 30 V bias, which was ≈30 times greater than the electrostrictive response. The effective piezoelectric coefficient was determined to be 1 nm V^−1^ at low frequencies and increased to 14 nm V^−1^ at resonance. A linear relationship between displacement and applied voltage was observed, indicating a robust piezoelectric effect in the graphene‐based membrane. Moreover, the resonance frequency was inversely proportional to the membrane size—smaller membranes demonstrated higher resonance frequencies but exhibited lower displacement amplitudes at resonance. These results underscore the potential of graphene‐based membranes for integration into micromechanical systems, including microactuators, micropumps, and microfluidic devices, where large displacements and mechanical flexibility are critical performance metrics.^[^ ^340^ ^]^

### 4.8 Micro‐/Nanoindentation

Micro‐ and nanoindentation techniques are widely employed to characterize the localized mechanical properties of graphene and its composites, including hardness, elastic modulus, and fracture toughness, at both micro‐ and nanoscale dimensions (Figure 22i).^[^ ^341^ ^]^ These methods involve applying a controlled load to a sharp indenter tip—commonly made of diamond/sapphire/silicon/silicon nitride/titanium alloys and shaped as Berkovich, cube‐corner, Vickers, conical, spherical, conospherical, and flat‐punch with a diameter of 100 nm to a few hundred microns—while it is pressed into the surface of the graphene‐based material. The resulting load–displacement response is recorded and analyzed using models such as the Oliver–Pharr method to extract quantitative mechanical parameters.^[^ ^342^ ^]^ Among these approaches,^[^ ^341^, ^343^ ^]^ AFM‐based nanoindentation with a nominal tip radius ≤ 100 nm is particularly effective for assessing the mechanical behavior of suspended graphene membranes due to its high spatial resolution and sensitivity. A summary of AFM nanoindentation applications in probing graphene's mechanical response is provided in Section 4.4.1.

**Figure 29:**
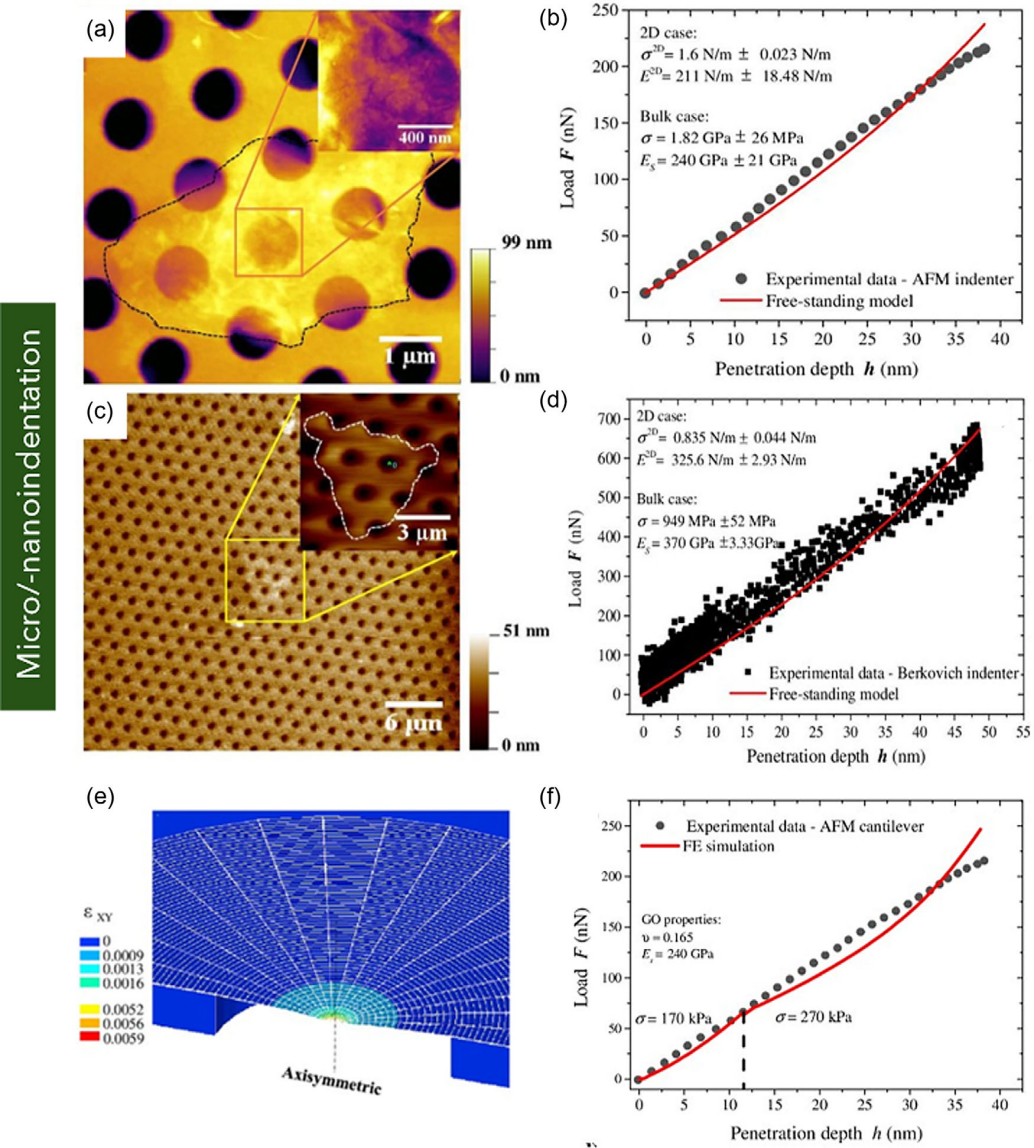
a,b) AFM‐derived Z‐height topography and the corresponding load–penetration depth curve obtained via AFM‐based nanoindentation. c,d) Z‐height image and load–penetration response acquired using instrumented nanoindentation with a Berkovich indenter. e) FEA illustrating the axisymmetric deformation field in the X–Y plane. f) Comparative analysis of load–penetration depth curves obtained from AFM nanoindentation and FEA. Reproduced with permission.^[^ ^345^ ^]^ Copyright 2021, Elsevier Ltd.

Instrumented nanoindentation has been extensively utilized to evaluate graphene‐based nanostructures’ stiffness and mechanical behavior. For example, Zhang et al.^[^ ^344^ ^]^ developed a straightforward yet effective method to simultaneously determine the mechanical properties and the number of GO layers using an instrumented nanoindenter. By accounting for the substrate influence, the elastic modulus of GO was measured to be ≈0.89 TPa, aligning well with both theoretical predictions and prior experimental observations. Moreover, a progressive reduction in hardness and elastic modulus was observed with increasing GO layer number, demonstrating a linear correlation between mechanical properties and layer thickness.^[^ ^344^ ^]^ In another study, Segovia et al.^[^ ^345^ ^]^ employed AFM‐based nanoindentation and instrumented nanoindentation to assess the mechanical response of multilayer GO sheets. GO was synthesized via a modified Hummer's method, yielding flakes with an average area of 3 μm^2^ and a surface roughness of ≈3 nm. Two distinct indentation systems were used: an AFM probe with a tip radius of 40 nm and a Berkovich indenter with a tip radius of 636 nm, allowing for comparative analysis. Their results indicated that the mechanical properties of GO varied with layer number (**Figure** **29**a–d). Upon normalization, both methods converged on a bulk elastic modulus of ≈130 GPa, though individual measurements yielded values of 240 GPa (AFM) and 370 GPa (Berkovich), respectively. FEA were further employed to estimate the pre‐stress levels in the GO sheets, revealing prestress values of 1.82 GPa (*σ*_2_D = 1.6 N m^−1^) for AFM indentation and 949 MPa (*σ*_2_D = 0.835 N m^−1^) for Berkovich indentation (Figure 29e–f). These discrepancies were primarily attributed to differences in tip geometry, with the AFM technique exhibiting greater variability in pre‐stress and stiffness measurements.^[^ ^345^ ^]^

In contrast to nanoindentation, microindentation techniques are primarily employed to evaluate graphene‐reinforced composites’ hardness and fracture behavior at the microscale. These methods are particularly suited for bulk or polycrystalline composite systems where l‐b‐l resolution is less critical. For instance, Song et al.^[^ ^346^ ^]^ examined the microscopic mechanical performance of MLG‐reinforced titanium matrix composites (TiMMCs) fabricated via SPS. Using a spherical indenter with a radius of 20 μm and applying loads ranging from 0 to 2000 mN, the group determined the elastic modulus and hardness of the composites. The TiMMCs incorporating 0.5 wt% MLG demonstrated a substantial improvement in mechanical properties—exhibiting a 71.4% increase in hardness (15.39 GPa) and a 6.4% enhancement in elastic modulus (264.25 GPa)—compared with pure titanium (8.98 GPa hardness, 248.37 GPa modulus). These enhancements were primarily attributed to efficient load transfer facilitated by graphene, along with strengthening mechanisms such as increased dislocation density and the Orowan looping mechanism.^[^ ^346^ ^]^ Similarly, Porwal et al.^[^ ^347^ ^]^ utilized microindentation to assess the fracture toughness and hardness of graphene‐reinforced alumina (Al_2_O_3_) composites with graphene volume fractions ranging from 0 to 5 vol%. The Vickers indentation method was employed, revealing that while the hardness of pure alumina was 22.9 GPa, adding 0.8 vol% graphene slightly reduced the hardness to 21.3 GPa. However, a notable increase in fracture toughness was observed, reaching 3.9 MPa $\sqrt{m}$ at 0.8 vol% graphene—an ≈40% improvement over the value for pure alumina (2.8 MPa $\sqrt{m}$). This enhancement was attributed to mechanisms such as crack bridging, deflection, and graphene pull‐out during crack propagation.^[^ ^347^ ^]^

### 4.9 Nanocomposite Tests

#### 4.9.1 Tensile Test

**Figure 30:**
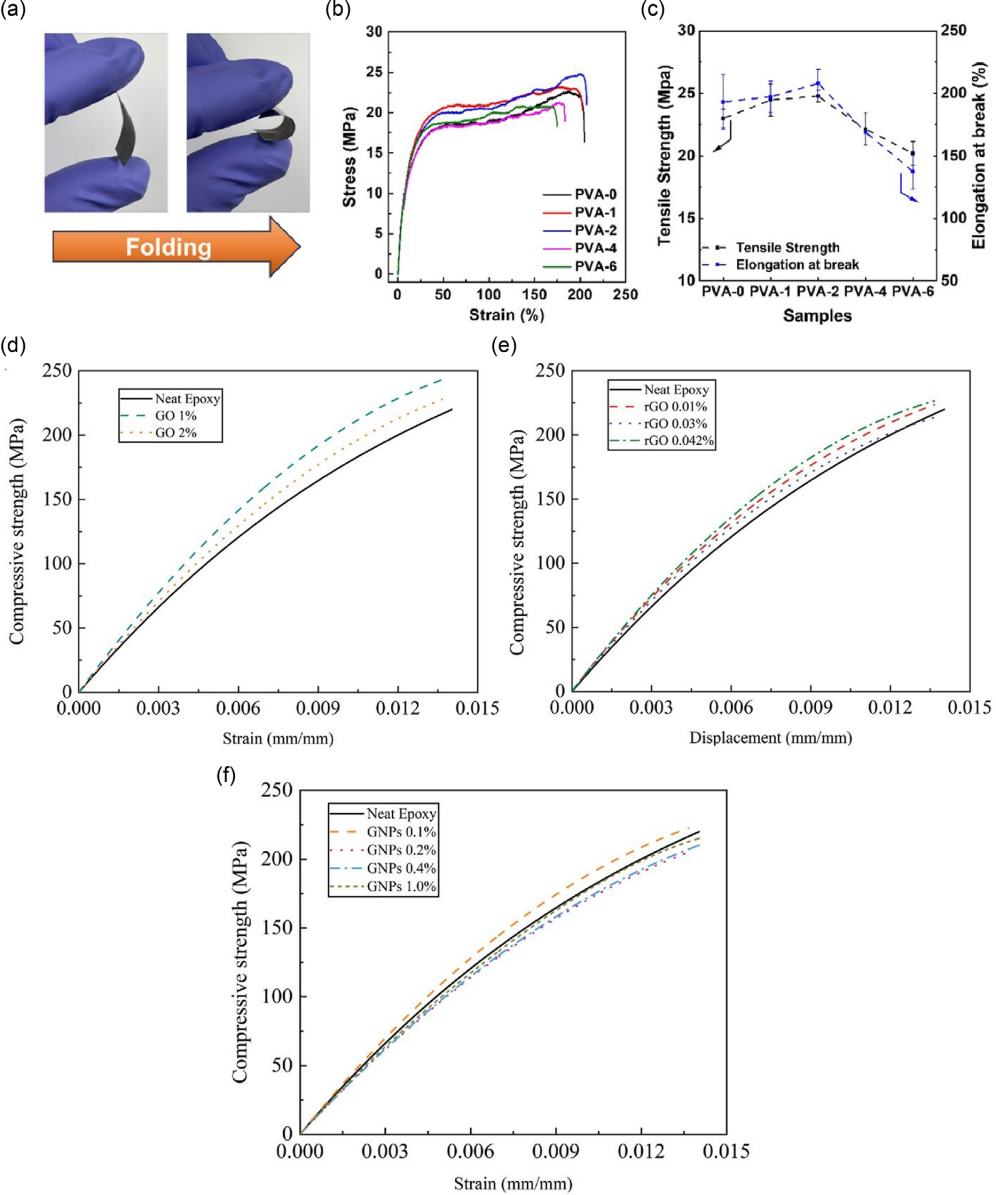
a) Demonstration of the mechanical flexibility of PVA/GF/GO composites. b) Stress–strain behavior of PVA/GF/GO composites as a function of GO loading. c) Comparison of tensile strength and elongation at break for PVA/GF/GO composites with varying GO content. (a–c) are reproduced with permission.^[^ ^351^ ^]^ Copyright 2024, Wiley Periodicals LLC. d–f) Stress–strain curves of graphene‐integrated glass fiber–epoxy composites incorporating d) GO, e) RGO, and f) GNP. (d–f) are reproduced under the terms of CC‐BY license.^[^ ^357^ ^]^ Copyright 2024, The Authors, published by Elsevier Ltd.

In contrast to the nano‐ and microscale mechanical characterization techniques discussed earlier, uniaxial tensile testing serves as a fundamental method for evaluating the bulk mechanical properties—including strength, stiffness, and ductility—of graphene‐based composites (Figure 22h). Graphene nanostructures, when embedded as reinforcement agents, significantly enhance the mechanical performance of various matrices such as polymers, metals, and ceramics.^[^ ^348^ ^]^ During tensile testing, a specimen is subjected to uniaxial tension while stress and strain are continuously monitored until failure. The resulting stress–strain curve provides key mechanical parameters, including Young's modulus, ultimate tensile strength (UTS), and elongation at break.^[^ ^349^, ^350^ ^]^ Remarkably, even at low graphene loadings (e.g., <1 wt%), substantial improvements in tensile strength and stiffness are observed. For instance, Jung et al.^[^ ^351^ ^]^ fabricated PVA‐based nanocomposites reinforced with graphene flakes (GF) and GO via solution mixing and casting. At 2 wt% GO loading, the PVA/GF/GO composites exhibited a 7.8% enhancement in tensile strength and a 15% increase in elongation at break compared to neat PVA (**Figure** **30**a–c). Similarly, Sahu et al.^[^ ^352^ ^]^ utilized twin‐screw extrusion followed by 3D printing to develop graphene nanoparticle (GN)‐reinforced polyurethane (PU) composites. While pristine PU exhibited Young's modulus of 800 MPa and tensile strength of 23 MPa, GN‐PU composites demonstrated progressive enhancements. Specifically, Young's modulus increased by 2%, 5%, 9%, and 25%, and tensile strength improved by 4%, 9%, 17%, and 26% for graphene loadings of 0.01, 0.02, 0.03, and 0.05 wt%, respectively.^[^ ^352^ ^]^ The observed enhancement in mechanical properties is primarily attributed to graphene's ability to act as an effective stress transfer medium, facilitating load distribution from the surrounding matrix to the reinforcing graphene layers. However, the extent of this improvement is highly dependent on several critical factors, including the interfacial bonding strength between graphene and the matrix, the uniformity of graphene dispersion, and the intrinsic defect density within the graphene structure.^[^ ^353^ ^]^ Poor dispersion or inadequate interfacial adhesion can lead to localized stress concentrations, which may initiate premature failure under loading conditions. To mitigate these limitations, graphene functionalization—achieved through chemical or physical surface modifications—has been widely adopted to enhance interfacial interactions and improve load transfer efficiency. In addition, structural parameters such as graphene flake size, orientation (alignment), and aspect ratio significantly influence the overall tensile performance of the composite system.^[^ ^354^ ^]^

#### 4.9.2 Compression Tests

Compression testing serves as a critical method for evaluating the bulk mechanical behavior of graphene‐based composites under compressive loading (Figure 22j). This technique provides key parameters such as compressive strength, modulus, and energy absorption capacity.^[^ ^355^ ^]^ Incorporating graphene nanosheets into polymer or metal matrices enhances compressive performance by impeding matrix deformation and improving load‐bearing capacity through effective stress transfer mechanisms. For instance, Sekhar et al.^[^ ^356^ ^]^ synthesized aluminum–graphene composites via a stir‐casting technique and observed substantial improvements in compressive strength due to the effective dispersion of graphene. Specifically, composites processed with stirring at 680 °C exhibited a significant increase in compressive strength, from 224.17 to 392.74 MPa, compared with their nonstirred counterparts. Similarly, Rafiee et al.^[^ ^357^ ^]^ employed vacuum‐assisted resin transfer molding to fabricate epoxy‐based composites reinforced with GO, RGO, and GNP. Compression testing revealed that GO and RGO notably enhanced the compressive modulus, with increases of 16.4% and 16.7% at 1 wt% and 2 wt% GO loadings, respectively (Figure 30d–f). However, composites incorporating GNPs at concentrations above 0.1 wt% exhibited reduced compressive strength, attributed to nanoparticle agglomeration and ineffective stress transfer. These performance enhancements were primarily linked to improved interfacial bonding between the graphene and the epoxy matrix, which mitigated crack initiation and propagation.^[^ ^357^ ^]^

The efficacy of compressive reinforcement is strongly governed by graphene dispersion, orientation, and interfacial adhesion. Random or poorly aligned graphene can limit the mechanical benefits under compressive loads. To overcome this, alignment techniques such as magnetic field‐assisted or shear‐induced orientation have been employed to optimize stress transfer along the graphene plane, thereby enhancing compressive strength.^[^ ^355^ ^]^ Furthermore, for porous graphene‐based materials such as aerogels and graphene foams, compression testing highlights their remarkable energy absorption, structural resilience, and recovery capability. These attributes arise from their intricate hierarchical architectures, rendering them highly promising for lightweight, impact‐resistant applications.^[^ ^358^ ^]^

#### 4.9.3 Flexural/Bending Tests

In addition to tensile and compressive testing, flexural (or bending) testing is a critical method for assessing the bulk mechanical behavior of graphene‐based composites under bending loads (Figure 22k).^[^ ^359^ ^]^ This test provides valuable information on flexural strength, flexural modulus, and failure mechanisms, which are particularly relevant for applications in lightweight structural components, surface coatings, and flexible electronic devices. Kaftelen–Odabaşı et al.^[^ ^360^ ^]^ investigated the influence of GNP loading (0.05, 0.25, and 1.25 wt%) on the flexural properties of carbon fiber–epoxy composites fabricated via vacuum infusion. At a low GNP concentration of 0.05 wt%, the composites exhibited a 6% increase in flexural strength and a 12.6% increase in storage modulus, attributed to improved GNP dispersion and enhanced fiber‐matrix interfacial adhesion. However, increasing the GNP content to 0.25 wt% resulted in a marginal decline in flexural strength, while a further increase to 1.25 wt% led to a significant reduction—≈33%—in flexural strength. This degradation was primarily due to GNP agglomeration and poor distribution within the epoxy matrix, which introduced stress concentration sites and weakened the composite structure. Despite the decline in strength at higher loadings, incorporating GNPs also enhanced the composite's energy dissipation capability, as evidenced by increased damping (tan δ) values—most notably in the 1.25 wt% GNP samples. These results underscore the importance of optimizing filler concentration to achieve a critical balance between reinforcement dispersion and mechanical performance in graphene‐based composite systems.^[^ ^360^ ^]^

#### 4.9.4 Hardness Tests

Hardness testing is a fundamental technique for assessing the resistance of graphene‐based composites to localized deformation, such as indentation or scratching (Figure 22l). The primary distinctions between conventional hardness tests and nanoindentation lie in their load and displacement ranges—with conventional tests operating in the gram‐to‐kilogram range and measuring displacements on the order of micrometers to millimeters, while nanoindentation employs micro‐ to millinewton loads and captures nanometer‐ to micrometer‐scale displacements. Standard hardness tests, including Vickers, Brinell, and nanoindentation, are commonly used to quantify a material's resistance to penetration under controlled loading conditions.^[^ ^361^ ^]^ Incorporating graphene nanostructures into polymeric, metallic, or ceramic matrices significantly improves composite hardness, owing to graphene's exceptional intrinsic strength, high Young's modulus, and superior load‐transfer capabilities. For example, Wang et al.^[^ ^362^ ^]^ introduced an in situ method for fabricating Cu/graphene–Al_2_O_3_ composites via hot‐press sintering. Vickers hardness testing revealed a substantial increase in hardness following adding aluminum isopropoxide, reaching a peak value of 89.3 HV. Subsequent cold rolling further enhanced the hardness to 121.5 HV, while the tensile strength increased to 394.1 MPa. These improvements were attributed to graphene's high specific surface area and strong interfacial interactions with the matrix, facilitating efficient load distribution and suppressing localized plastic deformation.^[^ ^362^ ^]^

#### 4.9.5 Impact Test

Impac*t* testing is a critical technique for assessing the energy absorption capacity, toughness, and resistance to sudden dynamic loading in composite materials (Figure 22m). This method evaluates a composite's ability to withstand high‐strain rate loading and dissipate energy prior to failure, making it especially relevant for applications in the aerospace, automotive, and protective equipment sectors.^[^ ^363^ ^]^ Incorporating graphene nanostructures significantly enhances the impact resistance of composites by facilitating efficient load transfer, stress redistribution, and energy dissipation during fracture events. Under impact loading conditions, graphene nanosheets serve as effective barriers that deflect, bridge, and delocalize stress, thereby delaying crack initiation and propagation. For instance, Doğan et al.^[^ ^364^ ^]^ fabricated GNP‐modified carbon/glass fiber hybrid composites via a vacuum‐assisted hand layup method. Impact testing was performed using a drop‐weight impact machine at an energy level of 30 J. The results demonstrated that incorporating 0.1 wt% GNP improved the impact resistance of the composites, attributed to improved interfacial load transfer and stress distribution. However, when the GNP content was increased to 0.5 wt%, a reduction in impact resistance was observed. This decline was likely due to GNP agglomeration and poor interfacial bonding, which compromised the composite system's structural integrity and energy dissipation efficiency.^[^ ^364^ ^]^

In addition to these tests, diamond anvil cell (DAC) studies are highly significant for understanding the mechanical properties of graphene because they enable precise probing of its structural and vibrational response under extreme pressures that cannot be achieved by conventional testing.^[^ ^365^ ^]^ By compressing graphene in a DAC and monitoring changes via in situ techniques such as Raman spectroscopy, several parameters may be obtained, including pressure‐induced shifts in phonon modes, quantifying compressibility, and assessing strain transfer efficiency. For example, Proctor et al.^[^ ^366^ ^]^ presented a high‐pressure Raman spectroscopy study of graphene using a DAC to investigate its mechanical properties under hydrostatic pressure up to 8 GPa. They reported that unsupported graphene under compression was intrinsically like graphite. For graphene supported on a silicon/SiO_2_ substrate, the DAC tests demonstrated that MG adhered to the substrate under compressive stress. A clear trend was observed where the pressure‐induced shifts of the Raman G and 2D peaks were larger for thinner samples. In contrast, thicker flakes did not adhere as well to the substrate. It highlighted the utility of high‐pressure DAC experiments in probing the mechanical and interfacial properties of graphene.

Overall, this section provides an overview of the experimental protocols employed to characterize the mechanical properties of graphene nanostructures. Techniques such as Raman spectroscopy, in situ SEM, TEM, and AFM are primarily utilized to probe mechanical behavior at the nanoscale, offering high‐resolution insights into local strain, stiffness, and defect‐induced deformation. In contrast, macroscale testing methods—including tensile, compressive, indentation, and impact testing—are applied to evaluate the bulk mechanical performance of graphene‐reinforced composites. The subsequent section will present a comparative analysis of key mechanical properties—such as Young's modulus, shear strength, and fracture toughness—across different graphene variants, highlighting their dependencies on the characterization techniques employed. **Table** **2** summarizes the key mechanical characterization techniques for graphene nanostructures.

**Table 2:** Summary of the mechanical characterization techniques utilized for graphene nanostructures.

Characterization technique	Principle	Applicability	Benefits	Limitations	References
Raman spectroscopy	It is based on the Raman effect, where the frequency of scattered light is measured. Inelastic scattering of incident light interacts with vibrating molecules. Such scattering is used to create the Raman spectra and probes the molecular vibrations	(1) Strain mapping using the G and 2D band of Raman spectra (2) Calculating Young's modulus from Raman shift	(1) Can detect nanometer‐scale strain variations (2) Nondestructive and noncontact testing	(1) Challenging to isolate the mechanical strain and the electronic doping‐induced Raman shifts (2) Grüneisen parameter for graphene varies between different studies and experimental conditions, resulting in potential discrepancies.	[402]
In situ SEM	Real‐time direct observation and quantification of deformation with the help of a PTP or a microtensile tester	(1) Measures the force‐displacement curves (2) Calculating Young's modulus, fracture, and tensile strength	(1) Provides visual confirmation of deformation/failure processes (2) Can be utilized to probe specific locations or defects	(1) Complex setup (2) Requires vacuum environment (3) Electron beam can potentially damage/alter the sample	[300]
TEM	Provides an atomic‐scale resolution of the MEMS‐induced deformation during quantitative tensile testing	(1) Measures the Young's modulus and tensile strength of graphene (2) Provides insights into atomic‐scale failure mechanism	(1) High spatial resolution (2) Direct measurement of force‐displacement curves	(1) Complex setup (2) Requires a vacuum environment	[310]
Atomic force microscopy	Using a sharp tip to apply a controlled indentation force to graphene	(1) Calculating the Young's modulus, breaking strength and maximum stress	(1) Minimal sample preparation requirement compared to SEM and TEM (2) Provides nanoscale resolution	(1) AFM tip can induce wrinkle or tear (2) Has high environmental sensitivity (3) Simplified assumptions for calculating modulus	[317]
Interferometry	It measures the light interference occurred during the out‐of‐plane deflection of strained graphene membranes	(1) Calculating pretension and displacement owing to applied force	(1) Has high sensitivity (2) Nondestructive and noncontact testing	(1) Complex experimental setup (2) Only suspended graphene can be characterized	[340]
Micro‐/nanoindentation	Measures a material's mechanical properties by continuously recording the force and displacement of an indenter as it deforms the surface	(1) Measures the hardness and fracture behavior of graphene‐based composites	(1) Ideal for thin films and coatings	(1) Inconsistent results owing to the indentation size effect (2) Poor measurement precision at low loads	[344]
Tensile testing	Axially pulls a material specimen at constant force or elongation until failure	(1) Provides the stress‐strain curves of composites (2) Measures Young's modulus and strength of composites	(1) Easy and reproducible (2) The stress‐strain curves provide multiple crucial mechanical parameters	(1) Destructive testing (2) Potential for operator error and misalignment	[351]
Compression testing	Applies compression to composites at constant force or displacement, while the applied force and displacement are recorded	(1) Measures the compressive strength, stiffness, and ductility of composites	(1) Low cost	(1) Long, slender specimen composites may show buckling (2) Friction between the compression plates and the composite specimen can affect the results	[357]
Flexural/bending test	Applies a bending load to a beam‐like composite supported by two supporting pins	(1) Measures flexural strength, flexural modulus (stiffness), and flexural strain at fracture	(1) Easy sample preparation (2) Provides results simulating real‐world conditions.	(1) Highly dependent on specimen geometry (2) Can show stress concentrations	[360]
Hardness test	A hard object (indenter) is used to scratch or indent the composite at a fixed force	(1) Measures the resistance to permanent indentation or scratching (2) Provides insight into wear resistance and composite strength	(1) It is fast (i.e., Rockwell technique) and straightforward (2) Low‐cost	(1) Requires careful surface preparation (grinding, polishing) (2) A damaged indenter may lead to inconsistent results	[362]
Impact test	A notched composite specimen is struck by a pendulum or hammer, and the energy absorbed in fracturing it is measured to assess toughness	(1) Measures toughness and fracture behavior	(1) Simple, quick, and inexpensive (2) Provides insights into damage tolerance and energy absorption capacity	(1) Results might vary owing to material anisotropy (2) Often it does not simulate real‐world conditions	[364]

## 5 Mechanical Properties of Graphene Nanostructures

Mechanical properties play a pivotal role in determining the structural integrity, functional performance, and application‐specific reliability of graphene nanostructures. Key mechanical parameters—such as Young's modulus (elastic modulus), tensile strength, shear strength, and localized point stresses at the nanoscale—have been extensively investigated to evaluate mono‐/bi‐/multilayer graphene and their variants’ (GNRs and PG) potential in advanced material systems. For example, Lee et al.^[^ ^41^ ^]^ employed AFM‐based nanoindentation to measure the mechanical properties of suspended MG films, reporting a Young's modulus of ≈1.0 TPa and a breaking strength of 42 N m^−1^.^[^ ^41^ ^]^ Mazilova et al.^[^ ^367^ ^]^ utilized a two‐chamber field ion microscope (FIM) operating at 78 K to determine the intrinsic tensile strength of GNRs, which was measured to be 99.34 GPa. Interfacial mechanics have also been explored. Daly et al.^[^ ^368^ ^]^ investigated the interfacial shear strength of multilayer GO films using a GO‐to‐GO friction force microscopy technique. Their findings revealed an interfacial shear strength of 5.3 ± 3.2 MPa, substantially lower than the theoretically predicted range of 17–132 MPa, depending on the assumed interlayer configurations. Furthermore, Suk et al.^[^ ^161^ ^]^ combined AFM and FEA to evaluate the pre‐stress in GO membranes, reporting values ranging from 39.7 to 76.8 MPa. Their study revealed that the prestress in GO membranes is approximately an order of magnitude lower than that of mechanically exfoliated MG, underscoring the influence of fabrication method and structural morphology on mechanical behavior.

**Figure 31:**
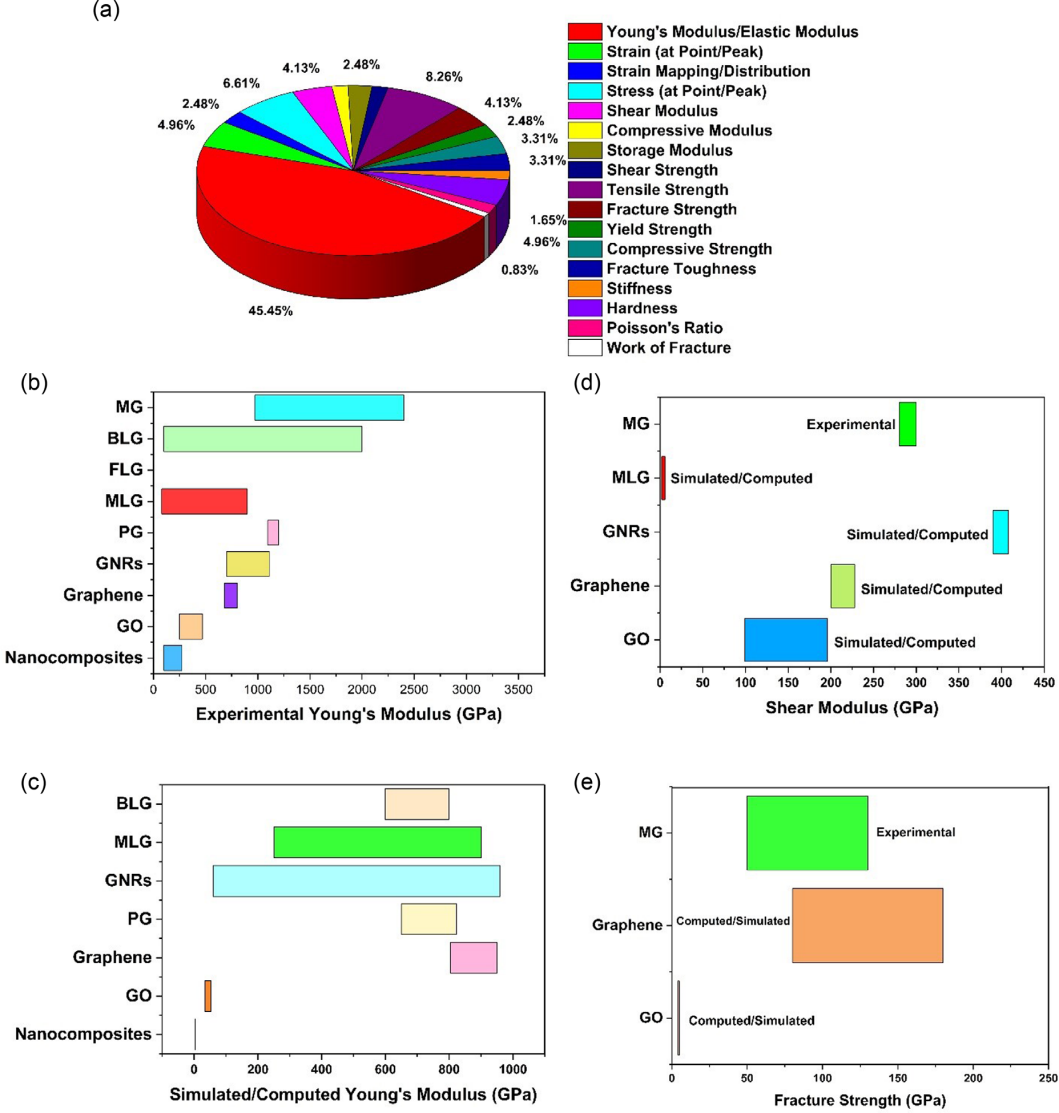
a) Pie chart illustrating the distribution of mechanical parameters investigated for various graphene nanostructures. b) Experimentally measured Young's modulus values. c) Simulated Young's modulus obtained from computational studies. d,e) Simulated and experimental shear modulus and fracture strength.

In contrast to pristine graphene, the mechanical performance of graphene‐based nanocomposites is predominantly characterized by parameters such as storage modulus, fracture toughness, stiffness, and hardness, which reflect their macroscopic functional enhancement. These properties are strongly influenced by the dispersion, interfacial bonding, and concentration of graphene nanofillers within the matrix. For instance, Gupta et al.^[^ ^369^ ^]^ synthesized GNP‐reinforced shape memory polyurethane (SMP) composites via a melt‐blending technique with varying GNP contents of 0.2, 0.4, 0.6, and 0.8 phr (parts per hundred rubber). Using dynamic mechanical analysis, the storage modulus of the composites containing 0.6 phr GNPs was reported to range from 9.2 to 15.1 MPa. Chen et al.^[^ ^370^ ^]^ employed a hot‐pressing technique to fabricate graphene nanosheet (GNS)‐reinforced alumina ceramics with GNS loadings of 0.1, 0.2, 0.5, and 1 wt%. The highest fracture toughness, measured at 6.6 MPa $\sqrt{m}$, was achieved at 0.2 wt% GNS loading. In another study, Chu et al.^[^ ^371^ ^]^ examined the mechanical reinforcement of carbon fiber‐reinforced epoxy composites through graphene incorporation. The addition of 2 wt% graphene into the epoxy matrix resulted in a 10 GPa increase in axial stiffness and a 200 MPa enhancement in axial strength, highlighting graphene's significant role in load transfer and matrix toughening. Additionally, Kurapova et al.^[^ ^372^ ^]^ fabricated graphene–Ni nanocomposites using ball milling and cold pressing techniques with graphene concentrations of 0.1 and 1 wt%. The highest hardness, 181 ± 39 HV, was observed for the 0.1 wt% graphene–Ni sample, as measured using a microindentation technique. A summary of these mechanical properties for representative graphene nanostructures is presented in **Figure** **31**a as a pie chart.

Among various graphene variants, MG has been the most extensively studied in terms of mechanical properties. However, the reported values vary significantly, as illustrated in Figure 31b. These discrepancies primarily arise from differences in testing techniques, substrates, and experimental parameters. For example, Young's modulus of MG was first reported by Lee et al.^[^ ^41^ ^]^ in 2008 as ≈1.0 TPa, measured using AFM. In contrast, Weng et al.^[^ ^292^ ^]^ employed Raman spectroscopy with a micrometer‐scale indenter tip to assess CVD‐grown MG and observed a higher Young's modulus of 1.48 TPa. Abooalizadeh et al.^[^ ^373^ ^]^ reported a slightly lower value of 0.973 TPa for MG supported on a silicon substrate using the contact resonance AFM (CR‐AFM) method. Additionally, Lee et al.^[^ ^291^ ^]^ measured a significantly elevated Young's modulus of 2.4 ± 0.4 TPa by bulging graphene balloons under applied pressure and quantifying strain distributions via Raman mapping. These variations can be partially attributed to the strain‐dependent behavior of Young's modulus.^[^ ^291^ ^]^ Notably, they also applied a very small strain of 0.19%, which is orders of magnitude lower than the strain ranges used by Lee et al.^[^ ^41^ ^]^ (0%–5% strain) and Weng et al.^[^ ^292^ ^]^ thereby contributing to the observed deviation in reported modulus values. Overall, lower values (≈1.0 TPa) typically arise in AFM‐based indentation tests due to localized defects, edge effects, and higher applied strains, while higher values (≈2.4 TPa) are often observed in bulge tests where strain is uniformly distributed and defect influence is minimized. Substrate effects, grain boundaries in CVD‐grown samples, and variations in strain amplitude all contribute to the wide spread of reported Young's modulus values for MG. In addition, a decreasing trend in Young's modulus from MG to BLG, FLG, and MLG has been consistently observed, primarily due to structural and interfacial effects. In BLG, FLG, and MLG, the layers are held together by weak vdW interactions, which are significantly weaker than the in‐plane sp^2^ covalent bonds. These weak interlayer forces render the structure more susceptible to interlayer sliding, delamination, and nonuniform deformation under applied stress. Furthermore, the inefficient load transfer across graphene layers, resulting from these weak vdW interactions, contributes to the overall reduction in the effective stiffness of the multilayer systems.

As shown in Figure 31b, PG and GO exhibit significantly lower experimental Young's modulus values compared with MG. For instance, Suk et al.^[^ ^374^ ^]^ reported a Young's modulus of 0.95 ± 0.12 TPa for CVD‐grown PG using a bulge test coupled with Raman spectroscopy. In a subsequent study, the same group evaluated the mechanical properties of monolayer GO using a similar bulge test combined with Raman mapping, obtaining Young's modulus of 207.6 ± 23.4 GPa—substantially lower than that of MG.^[^ ^161^ ^]^ The reduced stiffness in PG arises from its polycrystalline nature, consisting of multiple crystalline grains separated by grain boundaries. These boundaries introduce a high density of defects, such as pentagon–heptagon dislocations, vacancies, and lattice misorientations, which disrupt the continuity of the sp^2^‐bonded hexagonal network and act as stress concentrators, thereby weakening the material under load. As a result, PG retains stiffness values on the order of ≈1 TPa but consistently below MG, reflecting the balance between intact grain regions (which preserve sp^2^ stiffness) and weakened grain boundaries (which reduce load transfer efficiency). In the case of GO, the presence of oxygen‐containing functional groups (e.g., ‐OH, epoxy, and ‐COOH) covalently bonded to the basal plane and edges results in partial conversion of sp^2^ to sp^3^ hybridization. This chemical modification breaks the delocalized π‐bonding network, reducing in‐plane stiffness. Furthermore, the oxidation process introduces additional structural defects such as vacancies and distortions, which further compromise mechanical integrity. GO also tends to absorb water and exhibit enlarged interlayer spacing in multilayer configurations, increasing its flexibility and contributing to the observed reduction in Young's modulus. Consequently, GO displays a far greater modulus reduction than PG, since both chemical functionalization and structural disorder act simultaneously, leading to values (≈200 GPa) that are an order of magnitude lower than MG.

In addition to experimental approaches, computational techniques have been extensively utilized to simulate and predict the mechanical properties of various graphene‐based structures, including MG, BLG, GNRs, PG, GO, and graphene‐based nanocomposites, as shown in Figure 31c. However, significant discrepancies are often observed between experimentally measured and simulated values. For example, Amal et al.^[^ ^375^ ^]^ conducted MD simulations to investigate the nanoindentation behavior of BLG. Based on the force–deflection response obtained from the simulations, the calculated Young's modulus was ≈0.8 TPa. In contrast, an experimental study by Lee et al.^[^ ^291^ ^]^ using a bulge test combined with Raman spectroscopy to assess BLG under applied pressure, reported a substantially higher Young's modulus of 2.0 ± 0.5 TPa. This difference likely arises because MD simulations often employ defect‐free and idealized structures, while experimental BLG samples may experience interlayer coupling, defects, and pressure‐induced strain redistributions that increase the apparent stiffness. Konstantinova et al.^[^ ^376^ ^]^ leveraged ab initio calculations and tabulated the Young's modulus of MG to be 1.24 TPa, which was higher and lower than the experimental values reported by Lee et al. (1.0 TPa)^[^ ^41^ ^]^ and Lee et al. (≈2.4 TPa),^[^ ^291^ ^]^ respectively. Such variability underscores how sensitive Young's modulus is to sample quality, testing method, and environmental effects. In contrast, computational models capture intrinsic lattice stiffness, whereas experimental measurements reflect extrinsic influences such as wrinkles, adsorbates, and substrate interactions. These deviations can be attributed to several factors, including input parameters, structural defects, substrate interactions, and out‐of‐plane wrinkling in experimental samples—not all of which are typically accounted for in computational models.

Abreast of Young's modulus, other key mechanical properties such as shear modulus and fracture strength have also been investigated through experimental and computational approaches (Figure 31d,e). For instance, Min et al.^[^ ^377^ ^]^ employed MD simulations to predict the shear modulus and fracture strength of bulk graphene composed of 3,936 carbon atoms, with a simulated domain size of 100.8 × 102.2 Å^2^ under varying temperature conditions (300, 800, and 1700 K). The study revealed that the shear strength was ≈60 GPa at 300 K and decreased to 30 GPa at 1700 K, indicating a strong temperature dependence. Furthermore, the fracture strength and Poisson's ratio (transverse strain a material experiences when subjected to axial strain) at 300 K were calculated to be 97.54 GPa and 0.21 ± 0.01, respectively. This pronounced temperature dependence can be attributed to enhanced atomic vibrations and phonon–phonon interactions at elevated temperatures, which reduce bond stability and lower the energy barrier for shear failure. Liu et al.^[^ ^378^ ^]^ measured the shear modulus of CVD‐grown MG using Raman spectroscopy and found an average shear modulus of 280 GPa. The relatively lower value compared to idealized simulations is likely due to the presence of grain boundaries, wrinkles, and point defects commonly introduced during CVD growth, all of which weaken in‐plane shear resistance. However, in Thomas et al.'s^[^ ^379^ ^]^ MD simulation study, the calculated shear modulus of MG was reported to be 479 GPa, higher than the experimental study. Such overestimation is typical of defect‐free atomistic simulations, where ideal lattice structures without imperfections or environmental influences lead to higher intrinsic stiffness compared to real CVD‐grown samples. Table S1, Supporting Information, provides a summary of the expected and experimentally observed mechanical properties of various graphene variants, highlighting key discrepancies and influencing factors.

The bending stiffness of graphene, a critical parameter governing its flexibility and stability in devices, has been investigated extensively through theoretical, computational, and experimental approaches. For example, Wei et al.^[^ ^380^ ^]^ presented a theoretical method to determine the bending rigidity and Gaussian bending stiffness of MG by combining DFT with the Helfrich Hamiltonian, arriving at values of 1.44 and –1.52 eV, respectively. These results highlight the intrinsic resistance of a single graphene sheet to out‐of‐plane deformation. In contrast, Berinskii et al.^[^ ^381^ ^]^ developed a discrete mechanical model that links macroscopic bending stiffness to atomic‐scale bond stiffness parameters. Their calculations yielded values in the range of 0.35–0.58 nN·nm (comparable to ≈1 eV), demonstrating consistency with DFT but also emphasizing that the choice of model and input parameters can strongly affect the predicted stiffness.

More importantly, experimental and hybrid computational‐experimental studies reveal that bending stiffness is not a fixed material constant but can vary with geometry and interlayer interactions. Han et al.^[^ ^382^ ^]^ reported that in FLG, bending stiffness decreases substantially with increasing bending angle due to a slip‐mediated bending mechanism. Here, interlayer shear and slippage act as stress‐relief channels, reducing the effective rigidity compared to the idealized single‐layer case. This explains why MG typically exhibits stiffness in the range of 1.2–1.7 eV, whereas FLG can soften by nearly 400%, becoming far more flexible than predicted by single‐layer theory. Table S2, Supporting Information provides a cross‐technique validation summary of different mechanical properties of graphene nanostructures.

Taken together, these results suggest several important trends. First, MG possesses relatively well‐defined bending stiffness, consistent across both atomistic simulations and continuum models. Second, the introduction of multiple layers fundamentally alters bending mechanics—not simply by additive stiffening but through competing mechanisms of interlayer coupling versus slip. Third, discrepancies between theoretical and experimental values can often be traced to whether models incorporate interlayer shear, defect states, or environmental constraints such as substrates. These findings indicate that bending stiffness is highly sensitive to both intrinsic factors (bond stiffness, atomic configuration) and extrinsic factors (layer number, strain state, interlayer interactions), making it a tunable property that directly impacts the design of flexible graphene‐based devices. **Table** **3** summarizes the synthesis methods, mechanical characterization techniques, and the measured mechanical properties of graphene nanostructures.

**Table 3:** Graphene nanostructures, synthesis, characterization techniques, and their mechanical properties.

Graphene nanostructure	Synthesis	Characterization technique	Mechanical properties	Reference
MG	Micromechanical cleaving of graphite	Raman mapping with integrated 4‐point bending	At a shift rate of 61 ± 2 cm, the max relaxed strain is 0.25% and the shear stress is 0.25 MPa	[403]
Suspended MG	Micromechanical exfoliation of graphite	AFM	2D elastic modulus‐350 N m^−1^ and a Young's modulus of 1 TPa	[404]
MG	CVD	AFM and Raman spectroscopy	Peak elastic modulus of 95 GPa	[405]
MG	Mechanical cleaving	CR‐AFM	Young's modulus of 0.973 ± 0.001 TPa	[373]
MG	–	Simulation/computation (MD simulation)	Peak Young's modulus of 0.95 and real Young's modulus of 0.973 GPa	[406]
MG	Mechanical cleaving	AFM nanoindentation and Raman spectroscopy integrated with micro tensile tester	Young's modulus of 800–1100 GPa	[407]
Suspended MG	CVD	Raman spectroscopy with micrometer tip	Young's modulus of 1.48 TPa	[292]
MG	CVD	SEM with integrated PTP device	Young's modulus of ≈1 TPa, with a maximum tensile strength of 60 GPa at a peak strain failure of 58%	[408]
MG	CVD	Bulge tests and Raman mapping	Young's modulus for single crystal graphene and PG of 0.95 ± 0.12 TPa and −0.79 ± 0.13 TPa Prestress for single crystal graphene and PG of 0.63 ± 0.20 and 0.57 ± 0.24 GPa	[374]
MG	CVD	SEM with integrated PTP device	Elastic modulus of 1 TPa Fracture strength of 110 GPa	[301]
Boron‐doped MG	Thermal treatment	Friction test, Nanoindentation	Elastic modulus of 329 ± 40 N m^−1^	[409]
MG	–	Raman spectroscopy	Young's modulus of MG of 2.4 ± 0.4 TPa	[291]
BLG	–	Raman spectroscopy	Young's modulus BLG of 2.0 ± 0.5 TPa	[291]
FLG/MLG	Mechanical exfoliation	AFM	Young's modulus of 900 GPa	[410]
MLG	Mechanical exfoliation	AFM	Maximum Young's modulus of 80 GPa	[411]
MLG	–	Simulation/computation (MD Simulation)	Young's modulus in the armchair and zigzag direction of 900 GPa, and the shear modulus for both zigzag and armchair directions of 445 GPa (before buckling)	[412]
GNR	–	Simulation/computation (DFT)	Poisson's ratio of 0.129 to 0.261 Shear modulus of 0.447–0.667 TPa	[413]
GNR	–	Simulation/computation (MD simulation)	Tensile strength of zigzag GNR and armchair GNR of 107 and 90 GPa	[414]
Graphene drums	CVD	AFM and Raman	Max deformation of 38 nm	[415]
PG	–	Simulation/computation (MD simulation)	Young's modulus reduces from 82% to 65% compared with single‐crystalline graphene, and the fracture strength decreases by 16% when grain size is reduced from 10 nm to 2.5 nm	[416]
γ‐Graphyne	–	Simulation/computation (MD simulation)	Young's modulus in reclined chair and zigzag direction of 0.586 TPa and 0.510 TPa Poisson's ratio in RC and ZZ direction of 0.48 and 0.64	[417]
Monolayer GO	Modified Hummer's method	AFM with finite element analysis	Young's Modulus‐207.6 ± 23.4 GPa and maximum layer prestress 90 MPa	[161]
GO	–	Simulation/computation (MD Simulation)	Young's modulus of 530 GPa, shear modulus of 228 GPa failure strain of 21%	[418]
Multilayer GO	–	TEM with integrated MEMS	Fracture toughness of 39 J m^−2^	[419]
GO Film	Inkjet printing	AFM nanoindentation	Young's modulus of 470 GPa shear modulus of 196 GPa Poisson's ratio of 0.197	[420]
RGO film	Modified Hummer's method and chemical reduction	Tensile test	Maximum tensile strength of 43 MPa	[421]
3D/vertical graphene	CVD	Micro indentation test	Young's modulus of >100 MPa	[422]
Wrinkled graphene	CVD	Raman with integrated microtensile tester	Young's modulus of 680 ± 16 GPa and shear moduli of 290 ± 10 GPa	[423]
Graphene	CVD	AFM	Peakforce of 128 nN	[424]
Graphene (unspecified)	Mechanical exfoliation	AFM	Young's modulus dropped when electric field strength exceeded 0.18 ± 0.03 V nm^−1^	[425]
Wrinkled graphene	–	Simulation/computation (MD simulation)	Shear modulus of 1106.79 MPa and strength of 612.06 MPa	[426]
Graphene	–	ML‐assisted MD simulations	Elastic modulus of 1.069 TPa, Fracture strength of 197.7 and failure strain of 0.346	[427]
Graphene (unspecified)	CVD	Raman spectroscopy and AFM	Maximum Young's modulus of 630 GPa	[428]
Hydrogenated ψ‐graphene	–	Simulation/computation (Vienna ab initio simulation package)	Young's modulus for pristine ψ‐graphene of ≈272.58 N m^−1^ and for hydrogenated ψ‐graphene of 171.70 N m^−1^	[429]
BLG‐PDMS composites	Chemical exfoliation of BLG and solution mixing	Raman mapping with integrated 4‐point bending	Maximum strain of 0.19%	[430]
BLG‐PMMA composites	CVD and spin coating	Berkovich instrumented nanoindentation and AFM nanoindentation	Elongation of ≈2.15% at stress of 0.19 MPa	[431]
MLG‐NFC composites	Solution mixing and vacuum filtration	Tensile testing	Addition of 1.25% MLG led to a Young's modulus of 16.9 GPa	[432]
Graphene‐based carbon fiber composites	–	Tensile and compressive tests	At 2 wt% graphene addition, the composite Young modulus increased from 114.5 ± 1.4 GPa to 124.1 ± 5.1 GPa, and the tensile strength increased from 2418 ± 151 MPa to 2636 ± 114 MPa	[270]
PPy‐ErGO composites	Oxidation of graphite and in situ polymerization	AFM	The spring constant (k) for PPy/ErGO composites is found to be >800 nN nm^−1^. In contrast, for pure polypyrrole (PPy), it is >100 nN nm^−1^	[433]
Graphene foam/aerogel	–	Micro‐/nanoindentation test	45% energy dissipation for 100 nm tip	[434]
Graphene/epoxy‐nanoclay composites	3D printing	Flexural/bending test	Flexural strength of 82 MPa, flexural modulus of 4.7 GPa	[435]
Multilayer GO on carbon fiber	Chemical grafting	AFM	Elastic modulus of 34–77 GPa, failure strain of 8%‐–5%, and strength of 4–5 GPa	[436]
HOPG	Mechanical exfoliation	AFM, friction force microscopy	Elastic modulus of 41.5 ± 0.08 GPa and contact stiffness of 0.11 ± 0.05 N m^−1^	[437]

Graphene simultaneously exhibits an exceptionally high in‐plane Young's modulus and tensile strength owing to the stiffness of the sp^2^ C—C bonds, but an unusually low bending rigidity (κ = 1.5 eV),^[^ ^383^ ^]^ smaller than in‐plane bond‐stretching energies. This disparity means that out‐of‐plane deformations—wrinkling, buckling, and intrinsic rippling—occur at very low energetic cost and strongly influence apparent in‐plane mechanical measurements. For instance, in tensile experiments on suspended graphene, initial strain is often consumed in flattening wrinkles and slack. Raman spectroscopy and nanoindentation experiments both show strain‐dependent increases in the apparent modulus, with values around ≈250 GPa^[^ ^384^ ^]^ in wrinkled membranes up to the intrinsic ≈1 TPa^[^ ^41^ ^]^ once out‐of‐plane morphology is removed. Under compression, the competition between in‐plane stiffness and bending rigidity leads to Euler‐type buckling at critical strains, smaller than those of typical bulk materials. This geometric instability explains the ubiquity of wrinkles in transferred or supported graphene sheets and their strong effect on transport and mechanical properties.

Moreover, the electronic structure adds an additional layer of coupling. Graphene's π orbitals, extending out of plane, are relatively compressible compared to the in‐plane σ bonds. Local out‐of‐plane compression or rippling alters π–π overlap and enhances π–σ hybridization, which in turn modifies bond lengths and the effective in‐plane modulus.^[^ ^385^ ^]^ DFT calculations suggest that such π‐orbital deformation can soften the in‐plane stiffness, consistent with experimental reports of strain‐dependent Raman G‐band shifts deviating from purely geometric models.^[^ ^386^ ^]^ Together, these observations demonstrate that graphene's “mechanical constants” are not fixed but depend critically on the interplay of bending rigidity, in‐plane stiffness, and the electronic flexibility of the π system, all of which manifest in phenomena such as buckling, wrinkling, and strain‐dependent modulus evolution.^[^ ^387^ ^]^

In addition, combining mechanical adhesion and friction remains crucial. For instance, Shin et al.^[^ ^388^ ^]^ associated mechanical adhesion and friction in graphene. Microscratch tests revealed a nearly thickness‐independent friction coefficient of ≈0.03 for mono‐, bi‐, and trilayer exfoliated and epitaxial graphene, indicating that interlayer sliding and surface interactions were largely governed by intrinsic graphene properties rather than layer number. Complementary ramping force nanoindentation tests determined the critical load—and corresponding mean pressure up to ≈6.8 GPa—required to penetrate or induce failure in the graphene film, offering a quantitative measure of adhesion energy. The close agreement between low friction coefficients and the high critical loads suggested that strong interfacial adhesion and robust mechanical integrity coexisted in these systems. Minor discrepancies arose due to differences in measurement scale, tip geometry, or environmental conditions, as friction reflected dynamic interfacial sliding, while adhesion energies from indentation represented quasi‐static bonding strength. Together, these complementary techniques provided a coherent picture of graphene's interfacial mechanics, highlighting both its resistance to detachment and propensity for low‐friction sliding.

Moving forward, advancing the understanding of graphene nanostructures’ mechanical properties requires integrated efforts in both simulation and experimental domains. Key areas for future investigation include establishing comprehensive defect–mechanics relationships that account for the combined effects of point, line, edge, and wrinkling defects; developing multiscale modeling frameworks that bridge atomic‐level simulations (e.g., MD and DFT) with continuum‐scale finite element methods; and exploring coupled mechanical–thermal–electrical behavior under various loading and environmental conditions. Additionally, there is a critical need to investigate the impact of extreme temperatures, humidity, and oxidative environments on fatigue resistance and functional integrity. Incorporating machine learning (ML) and artificial intelligence (AI) approaches holds significant promise for predicting mechanical performance based on structural and chemical descriptors, thereby enabling data‐driven material design and optimization. A detailed method selection flowchart outlining suitable experimental and computational techniques for different mechanical properties is provided in Figure S1, Supporting Information.

## 6 Conclusions and Perspectives

As research on graphene continues to advance, its potential in emerging and frontier applications expands correspondingly. The unique nanostructural features and mechanical properties of graphene play a pivotal role in developing next‐generation materials and devices. In this review, we comprehensively analyze various graphene nanostructures, their fabrication methodologies, and the associated mechanical characterization techniques. Initially, we examine different graphene nanostructures, including MG, BLG, FLG, MLG, and PG, along with their functionalization, fabrication processes, and applications. Variations in the number of graphene layers are closely associated with changes in key functional properties, including bandgap, electron mobility, electrical and thermal conductivity, as well as mechanical behavior. These property shifts have a direct impact on the performance and applications. The review then transitions to an in‐depth exploration of key graphene fabrication techniques such as mechanical cleaving and exfoliation, CVD, oxidation–reduction, and polymer composites, highlighting the quality and performance of the resulting graphene or composite materials. Incorporating graphene nanostructures into composite matrices—such as polymers, metals, or ceramics—enhances the resulting materials’ functional and mechanical properties. Although computational approaches, including coarse‐grained models, have been employed to investigate the interfacial interactions between graphene and the matrix, experimental validation remains limited. A further focal point is the mechanical characterization techniques applied to graphene and its composites, and the mechanical properties derived from these methods. We extensively review the determination of Young's modulus, shear modulus, and fracture strength of graphene and graphene composites. Notably, there is a significant discrepancy between experimental and theoretical data. From an experimental standpoint, the observed discrepancies primarily stem from challenges in sample preparation, structural imperfections, and uncertainties introduced by substrate interactions and measurement limitations. From a modeling perspective, the lack of consideration for out‐of‐plane deformations such as wrinkling and the difficulty in bridging atomistic‐scale phenomena with continuum‐level mechanical behavior contribute significantly to the mismatch. These issues highlight the necessity for further advancements in experimental characterization techniques and multiscale modeling approaches. These further advancements are essential for ensuring accurate measurement of the mechanical properties of graphene and all other 2D materials, facilitating their application, and supporting continued advancements in the field.

Despite significant advances in understanding the mechanical behavior of graphene and its derivatives, several quantitative research targets remain critical for advancing both measurement reliability and practical applications: 1) *strain transfer optimization* (achieving strain transfer efficiencies exceeding 90% in polymer‐supported or embedded graphene systems to ensure accurate stress mapping and load transfer during Raman or AFM‐based mechanical tests); 2) *measurement reproducibility* (reducing the uncertainty in experimentally determined Young's modulus to below 5% across independent AFM indentation, bulge, and Raman‐shift techniques through standardized calibration and cross‐validation); 3) *atomistic‐to‐continuum coupling* (developing multiscale frameworks that couple MD simulations with FEA or coarse‐grained models to predict stress localization at grain boundaries and defects, with less deviation between simulated and experimentally measured fracture strength); and 4) *ML‐based predictive modeling* (building data‐driven models trained on DFT and MD datasets to forecast elastic modulus, failure strain, and interfacial strength across graphene nanostructures, aiming for prediction accuracy (*R* ^2^) greater than 95% across varying defect densities, layer numbers, and functionalization levels).

Achieving these quantitative milestones would substantially improve cross‐technique consistency, allow standardized mechanical benchmarking of graphene variants, and facilitate the integration of graphene and its derivatives into high‐performance functional devices.

## Supporting Information

Supporting Information is available from the Wiley Online Library or from the author.

## Conflict of Interest

The authors declare no conflict of interest.

## Supporting information

Supplementary Material
