# Supplementary material for: Insights into Graphene Nanostructures, Fabrication Techniques, Mechanical, and Functional Behavior Characterization
Source: Small Sci. 2025 Oct 30;5(12):e202500272. doi: 10.1002/smsc.202500272 (PMC12697800; doi:10.1002/smsc.202500272)
Supplement: Supplementary file 1 — Supplementary Material [file SMSC-5-e202500272-s001.zip › smsc70147-sup-0001-SuppData-S1.pdf]

Supplementary Information

Insights into Graphene Nanostructures, Fabrication Techniques, Mechanical, and Functional Behavior Characterization

Ashfaqul Hoque Khadem[1], *Camili Brignoni Diaz*[2], *and Lihua Lou*[1]*

[1]NanoBio Mechanics & Manufacturing Laboratory, Department of Mechanical Engineering, College of Engineering, Computing, and Applied Science, Clemson University, Clemson, SC 29634, United States

[2]Department of Mechanical and Materials Engineering, Florida International University, Miami, FL 33174, United States

* Email: llou@clemson.edu

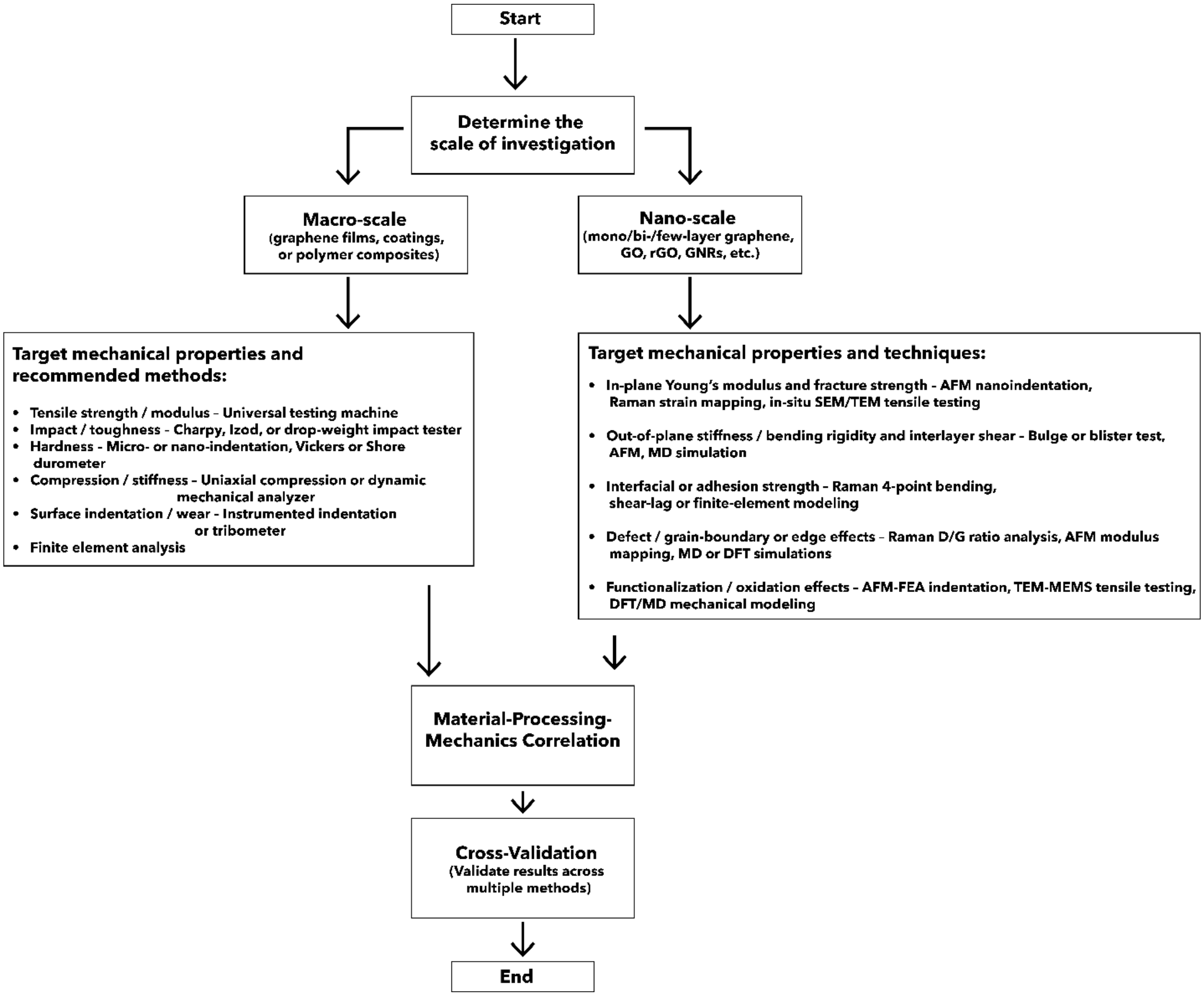

Figure S1. Method selection guide for mechanical characterization of graphene nanostructures and graphene-based composites at the macro and nanoscale levels.

Table S1. Summary depicting the expected vs. observed mechanical properties with key influencing factors.

Graphene Nanostructure	Expected / Intrinsic Mechanical Properties	Observed / Reported Mechanical Properties (with technique)	Influencing Factors	References
Monolayer Graphene (MG)	$E \approx$ 1.0–1.1 TPa; σ ≈ 130 GPa	E = 0.8–1.48 TPa; σ = 60–130 GPa (AFM, Raman, SEM-PTP, Bulge Test)	Substrate adhesion, defect density, strain-transfer efficiency, wrinkles	[1]
Suspended MG	$E \approx$ 1.0 TPa	E = 0.95–1.05 TPa; prestress ≈ 0.6 GPa (AFM, Bulge Test)	Clamping tension, pre-strain, pressure calibration	[2]
Bilayer Graphene (BLG)	$E \approx$ 0.9–1.0 TPa (slight reduction from MG)	E = 0.6–0.9 TPa; E = 2.0 ± 0.5 TPa (Raman, AFM)	Stacking order, shear slippage	[1a]
Few-Layer / Multilayer Graphene (FLG / MLG)	$E \approx$ 1.0 TPa	E = 0.08–0.9 TPa (AFM, MD Simulation)	Shear deformation, vdW coupling, bending	[3]
Graphene Nanoribbons (GNRs)	$E \approx$ 0.45–0.7 TPa; σ ≈ 100 GPa (edge-dependent)	E = 0.45–0.67 TPa; σ = 90–107 GPa (DFT, MD Simulation)	Edge roughness, confinement, chirality	[4]
Graphene Drums / Membranes	$E \approx$ 0.8–1.0 TPa (flat membrane)	Max deflection ≈ 38 nm; E = 0.2–0.8 TPa (AFM, Raman)	Pre-tension, wrinkles, boundary effects	[5]
Polycrystalline Graphene (PG)	$E \approx$ 0.9–1.0 TPa (for monocrystalline graphene)	E = 0.65–0.82 × monocrystalline E; strength reduces by 16 % compared to MG (MD Simulation)	Grain boundaries, voids	[6]
Graphyne / ψ-Graphene	$E \approx$ 0.5 TPa; ν ≈ 0.4–0.6	E = 0.51–0.59 TPa; ν = 0.48–0.64 (MD Simulation, DFT)	sp/sp^2 bond ratio, structural instability	[7]

Graphene Oxide (GO)	*E* ≈ 0.3–0.5 TPa (expected reduction due to oxidation)	*E* = 0.21–0.53 TPa; *G* ≈ 0.20 TPa (AFM-FEA, TEM-MEMS, MD Simulation)	Functional groups, hydration, wrinkling	[8]
Wrinkled Graphene	*E* ≈ 0.8–1.0 TPa (if flattened)	*E* = 680 ± 16 GPa; *G* = 290 ± 10 GPa (Raman, MD Simulation)	Out-of-plane strain, surface corrugation	[9]
3D / Vertical Graphene	*E* ≈ 0.1–0.2 GPa (expected for porous networks)	*E* > 100 MPa (Micro-indentation)	Porosity, wall connectivity	[10]
Graphene Nanocomposites	Predicted by the micromechanical models (i.e., Mori-Tanka, rule of mixtures, etc.)	*E* = 4.7–124 GPa; σ increases by 10–15 % (Tensile, AFM, Bending)	Load transfer, interfacial adhesion	[11]
HOPG (Bulk Graphite)	$E_{in\text{-}plane}$ ≈ 1 TPa (theoretical)	*E* = 41.5 ± 0.08 GPa (AFM, FFM)	Interlayer sliding, surface effects	[12]

Note: *E* = in-plane elastic (Young's) modulus; σ = tensile (fracture) strength; *G* = shear modulus; *v* = Poisson's ratio. FFM= Friction force microscopy

Table S2. Cross-technique validation summary.

Parameter	Graphene Nanostructure	Technique Employed	Cross-Validation Assessment	References
In-plane Young's modulus	MG, BLG, FLG	AFM nanoindentation, Raman strain mapping, Bulge test, In-situ SEM-PTP tensile test	Well validated across four independent methods	[1a, 3a, 13]
Fracture strength	MG, Suspended MG	AFM nanoindentation, SEM-PTP tensile	Strong agreement between AFM and SEM-based tensile methods	[2a]
Bending rigidity	Suspended MG, MLG membranes	Bulge/blister test, AFM membrane deflection, MD simulation	Limited validation	[3c]
Edge/width-dependent modulus	GNRs	DFT, MD simulation	Simulation only; no direct experimental confirmation	[4]
Functionalization / oxidation effect	GO, Hydrogenated graphene	AFM nanoindentation, MD Simulation, DFT	Partially validated; experiments confirm qualitative trend only	[8a, 8b]
Interfacial shear strength	Graphene-polymer composites	Raman mapping, MD Simulation	Moderate validation; consistent trend between Raman and MD simulation	[11a]

References

[1] a) J.U. Lee, D. Yoon, H. Cheong, Estimation of Young's Modulus of Graphene by Raman Spectroscopy *Nano Lett.* **2012**, 12, 4444; b) K. Cao, S. Feng, Y. Han, L. Gao, T. Hue Ly, Z. Xu, Y. Lu, Elastic straining of free-standing monolayer graphene *Nat. Commun.* **2020**, 11, 284; c) J. W. Suk, Y. Hao, K. M. Liechti, R. S. Ruoff, Impact of Grain Boundaries on the Elastic Behavior of Transferred Polycrystalline Graphene *Chem. Mater.* **2020**, 32, 6078; d) R. Dettori, E. Cadelano, L. Colombo, Elastic fields and moduli in defected graphene *J. Phys. Cond. Matt.* **2012**, 24, 104020.

[2] a) S.W. Weng, W.H. Lin, W.B. Su, E.T. Hwu, P. Chen, T.R. Tsai, C.S. Chang, Estimating Young's modulus of graphene with Raman scattering enhanced by micrometer tip *Nanotechnol.* **2014**, 25, 255703; b) N. Clark, A. Oikonomou, A. Vijayaraghavan, Ultrafast quantitative nanomechanical mapping of suspended graphene *Phys. Status Solidi B.* **2013**, 250, 2672.

[3] a)S. Scharfenberg, D. Z. Rocklin, C. Chialvo, R. L. Weaver, P. M. Goldbart, N. Mason, Probing the mechanical properties of graphene using a corrugated elastic substrate *Appl. Phys. Lett.* **2011**, 98, 091908; b) M. Mucientes, R. McNair, A. Peasey, S. Shao, J. Wengraf, K. Lulla, B. J. Robinson, O. Kolosov, Mapping nanoscale dynamic properties of suspended and supported multi-layer graphene membranes via contact resonance and ultrasonic scanning probe microscopies *Nanotechnol.* **2020**, 31, 415702; c) L. Ruiz, W. Xia, Z. Meng, S. Keten, A coarse-grained model for the mechanical behavior of multi-layer graphene *Carbon* **2015**, 82, 103.

[4] a) R. Faccio, P. A. Denis, H. Pardo, C. Goyenola, Á. W. Mombrú, Mechanical properties of graphene nanoribbons *J. Phys. Condens. Matter.* **2009**, 21, 285304; b) Y. Zheng, N. Wei, Z. Fan, L. Xu, Z. Huang, Mechanical properties of grafold: a demonstration of strengthened graphene *Nanotechnol.* **2011**, 22, 405701.

[5] F. Colangelo, P. Pingue, V. Mišeikis, C. Coletti, F. Beltram, S. Roddaro, Mapping the mechanical properties of a graphene drum at the nanoscale *2D Mater.* **2019**, 6, 025005.

[6] M. Q. Chen, S. S. Quek, Z. D. Sha, C. H. Chiu, Q. X. Pei, Y. W. Zhang, Effects of grain size, temperature and strain rate on the mechanical properties of polycrystalline graphene – A molecular dynamics study *Carbon* **2015**, 85, 135.

[7] a)L. Liu, L. Jiao, X. Huang, Mechanical properties of hydrogenated ψ-graphene *J. Mol. Model.* **2023**, 29, 185; b) S. Ajori, R. Ansari, M. Mirnezhad, Mechanical properties of defective γ-graphyne using molecular dynamics simulations *Mater. Sci. Eng. A* **2013**, 561, 34.

[8] a) Z. Meng, R. A. Soler-Crespo, W. Xia, W. Gao, L. Ruiz, H. D. Espinosa, S. Keten, A coarse-grained model for the mechanical behavior of graphene oxide *Carbon* **2017**, 117, 476; b) C. Cao, S. Mukherjee, J. Y. Howe, D. D. Perovic, Y. Sun, C. V. Singh, T. Filleter, Nonlinear fracture toughness measurement and crack propagation resistance of functionalized graphene multilayers *Sci. Adv.*, 4, eaao7202; c) I. Nikolaou, H. Hallil, V. Conédéra, B. Plano, O. Tamarin, J.-L. Lachaud, D. Talaga, S. Bonhommeau, C. Dejous, D. Rebière, Electro-mechanical properties of inkjet-printed graphene oxide nanosheets *Phys. Status Sol. A* **2017**, 214, 1600492.

[9] B. Liu, C. Pavlou, Z. Wang, Y. Cang, C. Galiotis, G. Fytas, Determination of the elastic moduli of CVD graphene by probing graphene/polymer Bragg stacks *2D Mater.* **2021**, 8, 035040.

[10] K. Davami, Y. Jiang, J. Cortes, C. Lin, M. Shaygan, K. T. Turner, I. Bargatin, Tuning the mechanical properties of vertical graphene sheets through atomic layer deposition *Nanotechnol.* **2016**, 27, 155701.

[11] a)J. Chu, R. J. Young, T. J. A. Slater, T. L. Burnett, B. Coburn, L. Chichignoud, A. Vuilleumier, Z. Li, Realizing the theoretical stiffness of graphene in composites through confinement between carbon fibers *Compos. A Appl. Sci. Manufac.* **2018**, 113, 311; b) S. Gupta, B. McDonald, S. B. Carrizosa, C. Price, Microstructure, residual stress, and intermolecular force distribution maps of graphene/polymer hybrid composites: Nanoscale morphology-promoted synergistic effects *Compos. B Eng.* **2016**, 92, 175.

[12] Z. Abooalizadeh, L. J. Sudak, P. Egberts, Nanoscale spatial mapping of mechanical properties through dynamic atomic force microscopy *Beilstein J. nanotechnol.* **2019**, 10, 1332.

[13] R. J. Young, L. Gong, I. A. Kinloch, I. Riaz, R. Jalil, K. S. Novoselov, Strain Mapping in a Graphene Monolayer Nanocomposite *ACS Nano* **2011**, 5, 3079.
